# *Trans*-anethole Ameliorates Intestinal Injury Through Activation of Nrf2 Signaling Pathway in Subclinical Necrotic Enteritis-Induced Broilers

**DOI:** 10.3389/fvets.2022.877066

**Published:** 2022-04-18

**Authors:** Caiyun Yu, Yichun Tong, Qiming Li, Tian Wang, Zaibin Yang

**Affiliations:** ^1^College of Animal Sciences and Technology, Nanjing Agricultural University, Nanjing, China; ^2^College of Animal Sciences and Technology, Shandong Agricultural University, Tai'an, China

**Keywords:** *Trans*-anethole, broiler, subclinical necrotic enteritis, intestinal oxidative stress, intestinal mucosal repair factor

## Abstract

This study was conducted to investigate the alleviative effects of *trans*-anethole (TA) on intestinal oxidative stress by enhancing the activities of intestinal antioxidant enzymes and activating the Nrf2 signaling pathway in subclinical necrotic enteritis (NE) infected broilers. A total of 192 1-day-old male Arbor Acres broilers were randomly allocated into three treatment groups: (1) control (CON); (2) subclinical NE challenge (NE); (3) NE challenge + 600 mg/kg TA (NE+TA600). Subclinical NE was induced by oral administration of live coccidiosis vaccine containing 2 × 10^4^ oocysts at 10 days of age and 2 ml of *Clostridium perfringens* type A solution (3 × 10^8^ CFU/ml) daily from days 14 to 19. The results showed that NE infection led to a severe decline (*p* < 0.05) in the final body weight (BW) and average daily gain (ADG), but an increase (*p* < 0.05) in feed/gain (F/G) of broilers at day 10–21 and day 1–21 compared with the control group. TA administration improved (*p* < 0.05) the growth performance of NE birds. The intestinal villus height (VH) and villus height/crypt depth (VH/CD) were reduced (*p* < 0.05) by NE challenge as compared with those of the control group, which was elevated by TA administration. Subclinical NE infection decreased (*p* < 0.05) serum activities of total superoxide dismutase (T-SOD), total antioxidant capacity (T-AOC), and jejunal and ileal glutathione peroxidase (GSH-PX), and T-SOD activity as well as T-AOC in the jejunum, while TA interventions positively elevated that (*p* < 0.05). Administration of TA protected the intestine against oxidative stress through up-regulation of intestinal nuclear factor erythroid 2-related factor 2 (Nrf2) signaling pathway as compared with the NE group (*p* < 0.05). In addition, dietary inclusion of TA elevated (*p* < 0.05) mRNA abundance of c-mesenchymal-epithelial transition factor (c-Met), jejunal epidermal growth factor receptor (EGFR), and transforming growth factor-beta 1 (TGF-β1) in the jejunum and ileum of birds after subclinical NE challenge. In conclusion, 600 mg/kg of TA may be a promising tool to prevent and control subclinical NE by increasing intestinal antioxidant status in broilers.

## Introduction

Necrotic enteritis (NE) is a worldwide high incidence of intestinal disease in poultry induced by *Clostridium perfringens* type A and C, or Net B (1, 2) and annually results in a global economic loss of 6 billion dollars (3). This infectious disease has been well-controlled for many years by the traditional use of antibiotics. Currently, the use of antibiotics has been banned due to the development of bacterial resistance and the production of antibiotic residues in poultry products (4). Therefore, there has been an increasing concern in exploring new strategies for prevention and treatment of NE other than the use of antibiotics.

Necrotic enteritis infection could result in intestinal lesions, intestinal inflammation, and intestinal oxidative stress (4, 5). Oxidative stress leads to the release of intracellular cytokines and further causes systemic and chronic inflammation (6). Oxidative stress is accompanied by increasing the production of reactive oxygen species (ROS) and is thought to be a trigger for intestinal injury (7, 8). Enhancement of endogenous antioxidant enzymes activities, such as superoxide dismutase (SOD), catalase (CAT), and glutathione peroxidase (GSH-PX), could eliminate the excessive production of ROS, and further ameliorate oxidative stress (9). Accordingly, fortifying the intestinal antioxidant status may be a promising strategy for preventing or treating NE in broilers.

*Trans*-anethole [TA, 1-methoxy-4-(prop-1-enyl) benzene], a major component (higher than 80%) of the essential oil extracted from the plant of anise and fennel, has been used as a flavoring agent in foods, cosmetics, alcoholic beverage, and perfumes and herbal medicine (10–12). It is a volatile terpenoid with anise flavor, poor water solubility, and easy to be deteriorated when exposed to light and high temperature. It is worth noting that TA is non-toxic with no genotoxic activity recognized by the United States Food and Drug Administration. Several recent studies have confirmed the antioxidant capacity of TA. It was demonstrated that TA sachets improved overall freshness and odor of organic ready-to-eat iceberg lettuce packages due to their antimicrobial and antioxidant activities (13). TA could prevent hydrogen peroxide-induced collagen metabolism alterations and apoptosis in human skin fibroblasts, proving that TA may be beneficial against oxidative stress (10). Moreover, our previous study found that star anise oil enhanced antioxidant status in laying hens (14). However, the mechanism by which the inclusion of TA enhanced antioxidant status of broilers has not been elucidated yet. Nuclear factor E2-related factor 2 (Nrf2), which is a member of the NF-E2 family of the basic leucine zipper of redox-sensitive transcription factors, is well known for scavenging free radicals and preventing oxidative stress (15, 16). It is a classic antioxidant signaling pathway that regulates the expression of phase II antioxidant enzyme genes against oxidative stress. Therefore, the present study aimed to investigate whether TA had alleviative potential on intestinal oxidative stress of subclinical NE challenged broilers through activating the Nrf2 signaling pathway.

## Materials and Methods

### Ethics Approval

This study was reviewed and approved by the Institutional Animal Care and Use Committee of Nanjing Agricultural University (Permit No. SYXK-2017-0027).

### Preparation of *Trans*-anethole

*Trans*-anethole was purchased from Nanjing Dilger Medical Technology Co., Ltd (D105737, Nanjing, China). The analyzed purity of TA was 98.35%. The TA was stored in the dark and at 4°C until use.

### Preparation of Bacterial Strain

Freeze-dried bacteria powder of *C. perfringens* type A strain (125404) was obtained from BeNa Culture Collection (Xinyang, China). It was cultured anaerobically with fluid thioglycollate (FT) medium (HB5190; Hopebio Biotechnology Co., Ltd, Qingdao, China) in panel for 24 h at 37°C, then aseptically picked single colony into 2 L conical flask with FT medium and anaerobically incubated by shaker for 13 h at 37°C.

### Animals, Diets, and Experimental Design

A total of 192 1-day-old male Arbor Acres broilers (42.43 ± 0.88 g) were purchased from Yantai Land Animal Husbandry (Shandong, China). On arrival, all birds were weighed and randomly allocated into three groups, with 8 replicates of 8 birds in each group. The basal diets were formulated to meet the bird's nutritional requirements according to NY/T 33-2004 [(17); Supplementary Table 1]. TA was blended with soybean oil and then mixed with other ingredients. All of the diets were pelleted and crumbled. All birds were reared in wire cages and had free access to diet and water throughout the entire experimental period. Room temperature was maintained at 33°C during the first 5 days and then gradually decreased by 0.5°C daily until 22°C. The treatment groups were as follows: (1) CON group (basal diet); (2) NE group (subclinical NE challenge); and (3) NE + TA600 group (subclinical NE challenge + 600 mg/kg of TA). The procedure of subclinical NE challenge was performed with minor modifications by Liu et al. (18) and Zhang et al. (19). Briefly, all chicks in the subclinical NE infected groups were each orally gavaged with live coccidiosis vaccine (Foshan Standard Biotechnology Co., Ltd, Guangdong, China) containing 2 × 10^4^ oocysts suspended in 500 μl of normal saline with coccidia suspension agent at 10 days of age and then with 2 ml of *C. perfringens* type A solution (3 × 10^8^ CFU/mL) daily from days 14 to 19. Birds in the CON group were orally gavaged with the same amount of normal saline at 10 days of age and sterile FT medium solution during days 14–19. TA was supplemented throughout the whole experimental period.

### Sample Collection

On the morning of day 22, 8 birds per group with average body weight (BW) of its replicate were selected for sampling. The serum samples were collected from peripheral blood, which was centrifuged at 3,500 × g for 10 min at 4°C, then were stored at −80°C until analysis. After blood sampling, the birds were stunned and sacrificed by cervical dislocation. Approximately 1 cm of middle segments of jejunum and ileum were cut off carefully and fixed in 4% paraformaldehyde solution for histomorphology analysis. Approximately 3 cm of middle jejunum and ileum segments of each bird were dissected and washed with ice-cold sterile saline, then frozen in liquid nitrogen and stored at −80°C for subsequent analysis.

### Growth Performance

The BW and feed intake of birds of each replicate were recorded weekly to calculate the average daily feed intake (ADFI), average daily gain (ADG), and feed/gain (F/G).

### Intestinal Morphology

The fixed jejunum and ileum segments were dehydrated, transparentized, and embedded in paraffin. Each sample was sliced into 5–μm cross-sections, deparaffinized in xylene, graded rehydrated, and finally stained with hematoxylin-eosin. With reference to Ekim et al. (20), 10 well-oriented villi and crypts per sample were selected for measuring the villus height (VH) and crypt depth (CD) using light microscope (Olympus CX31, Tokyo, Japan) and Image-Pro Plus 6.0 software (Media Cybernetics, Inc., Rockville, MD, USA).

### Determination of Antioxidant Capacity

Frozen jejunum and ileum were weighed, and homogenized (3 min) with ice-cold physiologic saline in the ratio of 1:4 (wt/vol). The homogenates were then centrifuged at 4,000 × g for 10 min at 4°C. The supernatants were then diluted into the optimal content for examining the activities of total SOD (A001-1-1), total antioxidant capacity (T-AOC; A015-1-2), and GSH-PX (A005-1-2), and the concentration of malondialdehyde (MDA; A003-1-2) using the assay kits (Nanjing Jiancheng Bioengineering Institute, Nanjing, China). The total protein concentration of supernatants was detected by bicinchoninic acid (BCA) protein assay kit (P0010; Beyotime Institute of Biotechnology, Nanjing, China). The results were expressed as activities of antioxidant enzyme and concentration of MDA in per mg of protein in the intestinal tissues of broilers. Additionally, the serum activities of T-SOD, T-AOC, GSH-PX, and concentration of MDA were also detected using the same assay kits and presented as that in per ml of serum.

### Quantitative Real-Time PCR Assay

Extraction of total RNA in the jejunum and ileum was performed using Trizol reagent (9108; TaKaRa Biotechnology, Dalian, Liaoning, China). The quality and concentration of total RNA were detected using a NanoDrop-1000 microspectrophotometer (Thermo Fisher Scientific, Waltham, MA, USA), and the integrity of extracted RNA was evaluated with 2.0% agarose gel electrophoresis. Subsequently, the reverse transcription polymerase chain reaction (PCR) was conducted to produce the complementary DNA using the PrimeScriptTMRT reagent Kit (RR036A; TaKaRa Biotechnology Co., Ltd, Dalian, China) by two steps: 37°C for 15 min and 85°C for 5 s. qRT-PCR reactions were conducted to determine the relative mRNA abundance of Nrf2, NAD(P)H quinone dehydrogenase 1 (NQO1), heme oxygenase 1 (HO1), superoxide dismutase 1 (SOD1), glutathione peroxidase (GSH-PX), epidermal growth factor receptor (EGFR), c-mesenchymal epithelial transition factor (c-Met), transforming growth factor-alpha (TGF-α), transforming growth factor-beta 1 (TGF-β1), and beta-actin (β-actin) ChamQ SYBR^®^ qPCR Master Mix Kit (Q311-02; Vazyme Biotechnology, Nanjing, China) based on Applied Biosystems 7500 Real–time PCR System (Life Technologies, CA, USA). The primers were commercially synthesized by Sangon Biotechnology Co., Ltd (Shanghai, China), which are shown in Supplementary Table 2. The amplification program consists of an initial denaturation step at 95°C for 30 s, followed by 40 cycles of 95°C for 10 s and 60°C for 30 s, then 15 s at 95°C, and 60 s at 60°C, with a final step at 95°C for 15 s. The relative mRNA abundance of target genes were analyzed using the 2-^ΔΔ*Ct*^ method and normalized against the reference gene (β-actin) expression level.

### Western Blot Assay

Total protein extracted from the jejunum and ileum tissues was performed by radioimmunoprecipitation assay lysis buffer and protease inhibitor (P0013B and ST506; Beyotime Institute of Biotechnology, Nanjing, China). Nuclear protein was isolated by Nuclear and Cytoplasmic Protein Extraction Kit (P0027; Beyotime Institute of Biotechnology, Nanjing, China) for detection of Nrf2 protein expression. The concentrations of total cellular protein and nuclear protein were detected by the BCA assay kit (P0010; Beyotime Institute of Biotechnology, Nanjing, China). Equal amounts of protein were separated through sodium dodecylsulfate polyacrylamide gel electrophoresis (SDS-PAGE), transferred onto polyvinylidene difluoride (PVDF) membranes. Subsequently, the membranes were blocked with 5% skimmed milk (w/v) in tris-buffered saline with 0.1% tween (TBST) buffer for 2 h at room temperature, and then incubated with primary antibodies against Nrf2 (16396-1-AP; Proteintech Group, Inc., Wuhan, China), HO1 (10701-1-AP; Proteintech), SOD1 (10269-1-AP; Proteintech), and β-actin (20536-1-AP; Proteintech) overnight at 4°C, and then incubated with secondary goat anti-rabbit IgG horseradish peroxidase-conjugated antibody for 1.5 h at room temperature. The expression of target proteins was determined using ECL chemiluminescence reagents (E412-01; Vazyme Biotechnology) and images were captured by Imager-Bio-Rad (Bio-Rad Laboratories, Inc., Hercules, CA, USA). The band intensities were quantified using Image J software.

### Statistical Analysis

All data were presented as mean ± standard error of mean. The Shapiro–Wilk test was used to determine the dataset normality and homogeneity of variances. Data sets were analyzed using one-way analysis of variance (ANOVA), followed by Tukey's HSD test (SAS Institute, 2001). Results were regarded statistically significant with a *p* < 0.05.

## Results

### Growth Performance

No mortality was observed in the prevention groups during the experimental period. NE infection led to a severe decline (*p* < 0.05) in the final BW and ADG, but an increase (*p* < 0.05) in F/G of broilers at day 10–21 and 1–21 compared with control groups (Table 1), but TA administration reversely altered (*P* < 0.05) those of NE birds. No remarkable difference was found with regards to initial BW, and ADFI at day 0–9 and 1–21 among groups (*p* > 0.05), but the ADFI at day 10–21 tended to be decreased (*p* = 0.074) by NE infection. Moreover, the ADG and F/G were not altered (*p* > 0.05) by TA supplementation before NE infection.

**Table 1:** Effect of dietary TA supplementation on the growth performance of broilers challenged with subclinical necrotic enteritis^1^.

**Items^**2**^**	**CON**	**NE**	**NE + TA600**	***p-*value**
Initial BW, kg	42.03 ± 0.22	42.00 ± 0.54	42.07 ± 0.81	0.232
Final BW, kg	873.18 ± 24.42^a^	826.13 ± 24.34^b^	835.71 ± 38.99^ab^	0.004
**Pre-Challenge (0–9 d)**				
ADFI, g/d	27.79 ± 1.30	27.89 ± 0.80	27.81 ± 1.31	0.984
ADG, g/d	23.07 ± 0.71	23.32 ± 0.62	23.50 ± 1.31	0.689
F/G, g/g	1.20 ± 0.03	1.20 ± 0.04	1.19 ± 0.07	0.773
**Post-Challenge (10–21 d)**				
ADFI, g/d	68.19 ± 1.32	65.40 ± 2.42	67.65 ± 2.77	0.074
ADG, g/d	56.79 ± 0.47^a^	48.78 ± 1.86^c^	53.86 ± 2.22^b^	<0.001
F/G, g/g	1.20 ± 0.02^b^	1.34 ± 0.09^a^	1.26 ± 0.05^ab^	0.002
**Overall (1–21 d)**				
ADFI, g/d	50.79 ± 2.95	49.27 ± 2.92	50.36 ± 2.37	0.579
ADG, g/d	41.56 ± 0.42^a^	37.36 ± 1.09^c^	39.68 ± 1.51^b^	<0.001
F/G, g/g	1.22 ± 0.06^b^	1.32 ± 0.10^a^	1.27 ± 0.06^ab^	0.024

a−c *Means within a row with different letters differ significantly (p < 0.05)*.

1 *Data are means for 8 replicates of 6 birds per replicate. No birds died during the experimental period. The data in each group was expressed as mean with their standard errors (n = 8)*.

2 *ADFI, average daily feed intake; ADG, average daily gain; F/G, feed/gain; BW, body weight*.

### Intestinal Morphology

**Figure 1:**
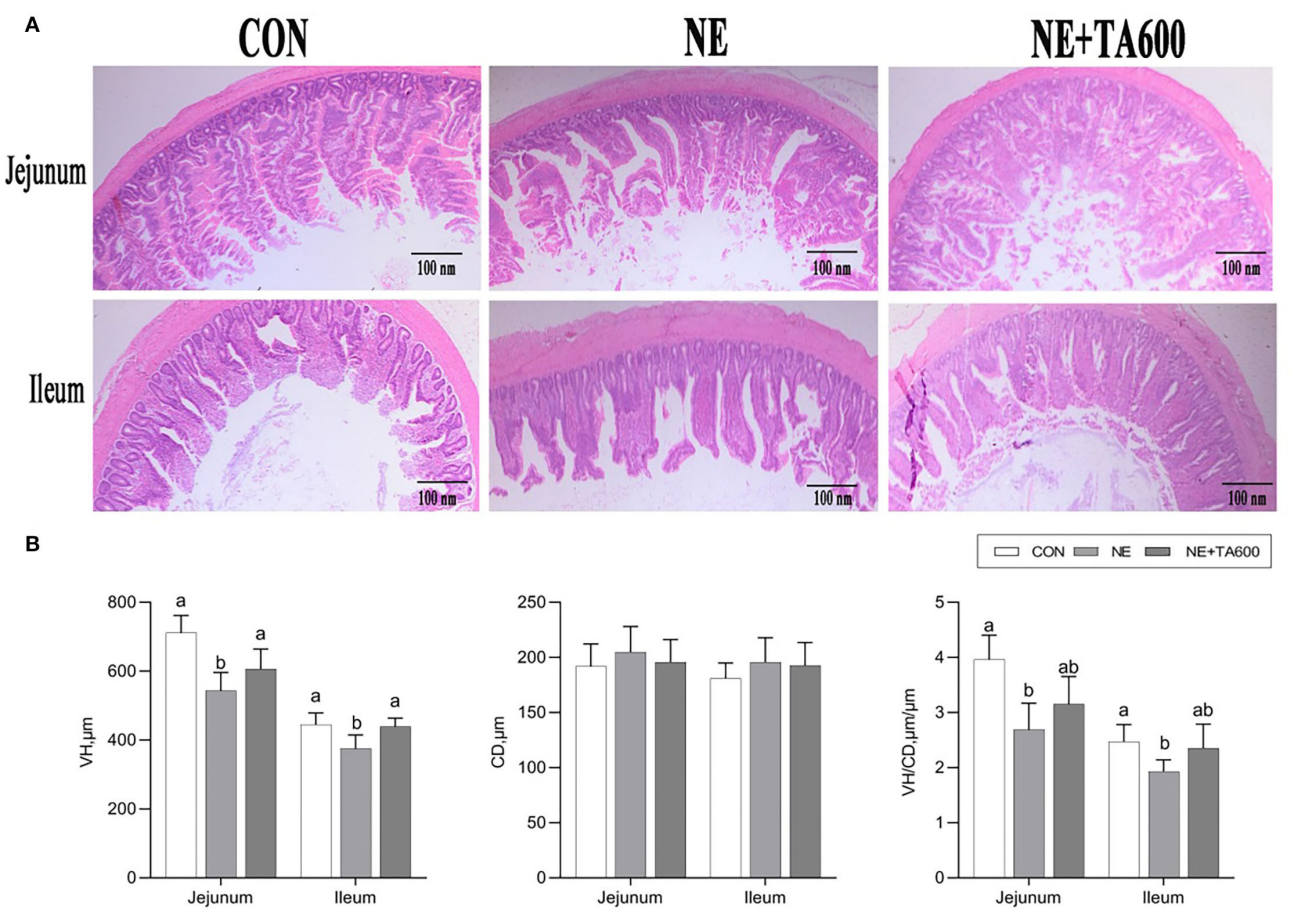
Effects of TA on intestinal morphology of subclinical NE broilers. **(A)** Hematoxylin and eosin staining, **(B)** VH, CD, and VH/CD of the intestine. Values were expressed as mean with standard error represented by vertical bars. ^a, b^Means with different letters differ significantly among the groups (*p* < 0.05). VH, villus height; CD, crypt depth.

Figure 1A revealed that there was some damage to jejunal and ileal villi development after NE infection, as found by broken and shortened villi. Consistent with the histological observations of tissue sections, NE infection significantly reduced the VH and VH/CD in jejunal and ileal tissues as compared with those of control (Figure 1B), which was elevated by TA supplementation (*p* < 0.05). In addition, the intestinal CD was not affected by NE challenge (*p* > 0.05).

### Activities of Antioxidant Enzymes

**Figure 2:**
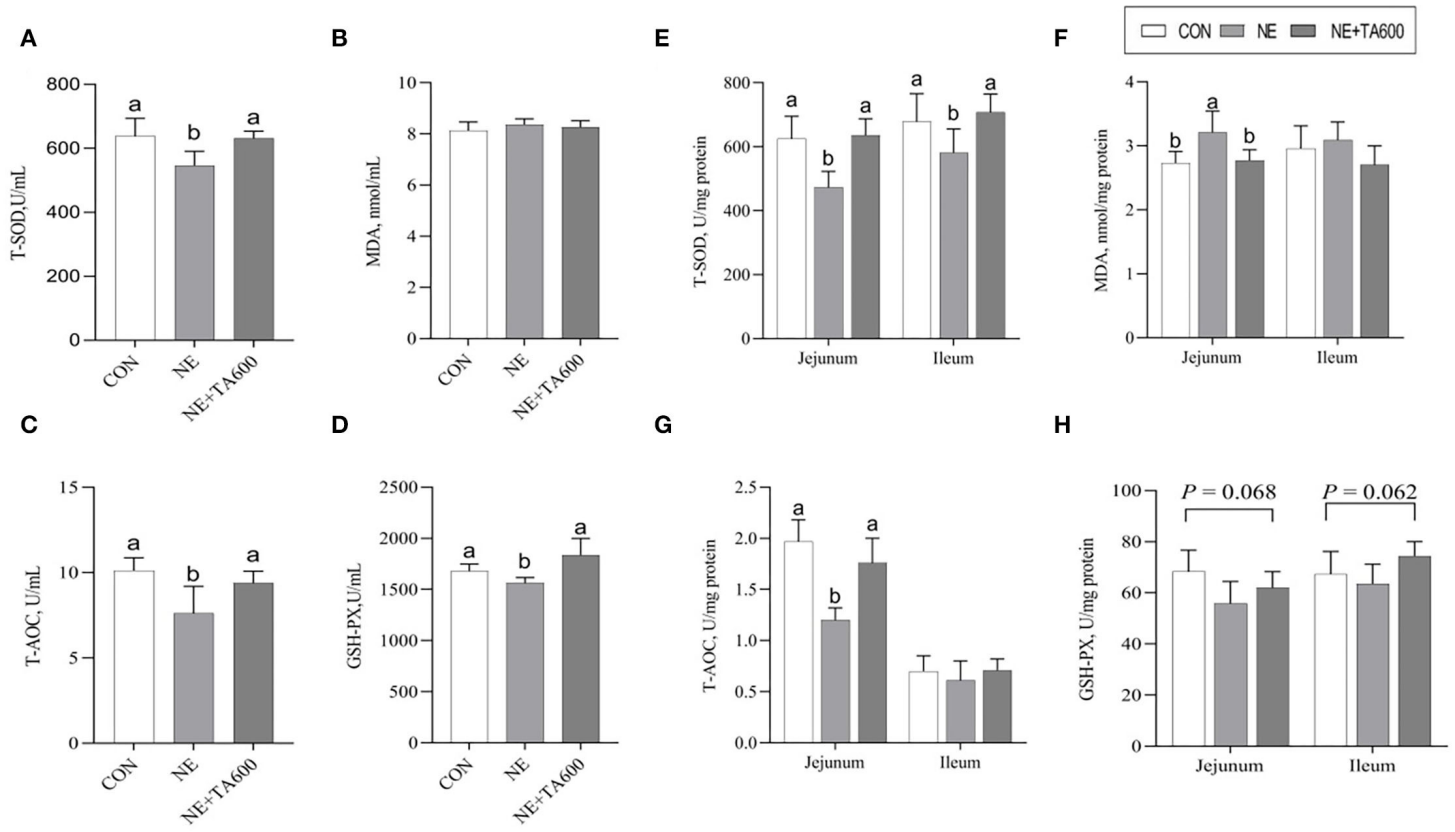
Effects of TA on the activities of antioxidant enzymes in the serum and intestine of subclinical NE broilers. **(A–D)** The activities of T-SOD, MDA, T-AOC, and GSH-PX of serum; **(E–H)** The activities of T-SOD, MDA, T-AOC, and GSH-PX in the jejunum and ileum. Values are means with their standard errors represented by vertical bars. ^a, b^Means within a row with different letters differ significantly (*p* < 0.05). T-SOD, total superoxide dismutase; MDA, malondialdehyde; T-AOC, total antioxidant capacity; GSH-PX, glutathione peroxidase.

As revealed by Figure 2, these data indicated that the serum activities of T-SOD, T-AOC, and GSH-PX were decreased (*p* < 0.05) by subclinical NE infection, but TA interventions elevated that (Figures 2A–D). Although no significant difference was shown with regards to ileal MDA concentration and T-AOC activity among the four groups, the inclusion of TA increased (*p* < 0.05) the T-SOD activity in the jejunum and ileum as well as T-AOC activity in the jejunum in comparison with subclinical NE group (Figures 2E–G). In addition, the GSH-PX activity in the jejunum (*p* = 0.068) and ileum (*p* = 0.062) was tended to be reduced after subclinical NE infection, but elevated by TA administration.

### Expression of Nrf2 Signaling Pathway

**Figure 3:**
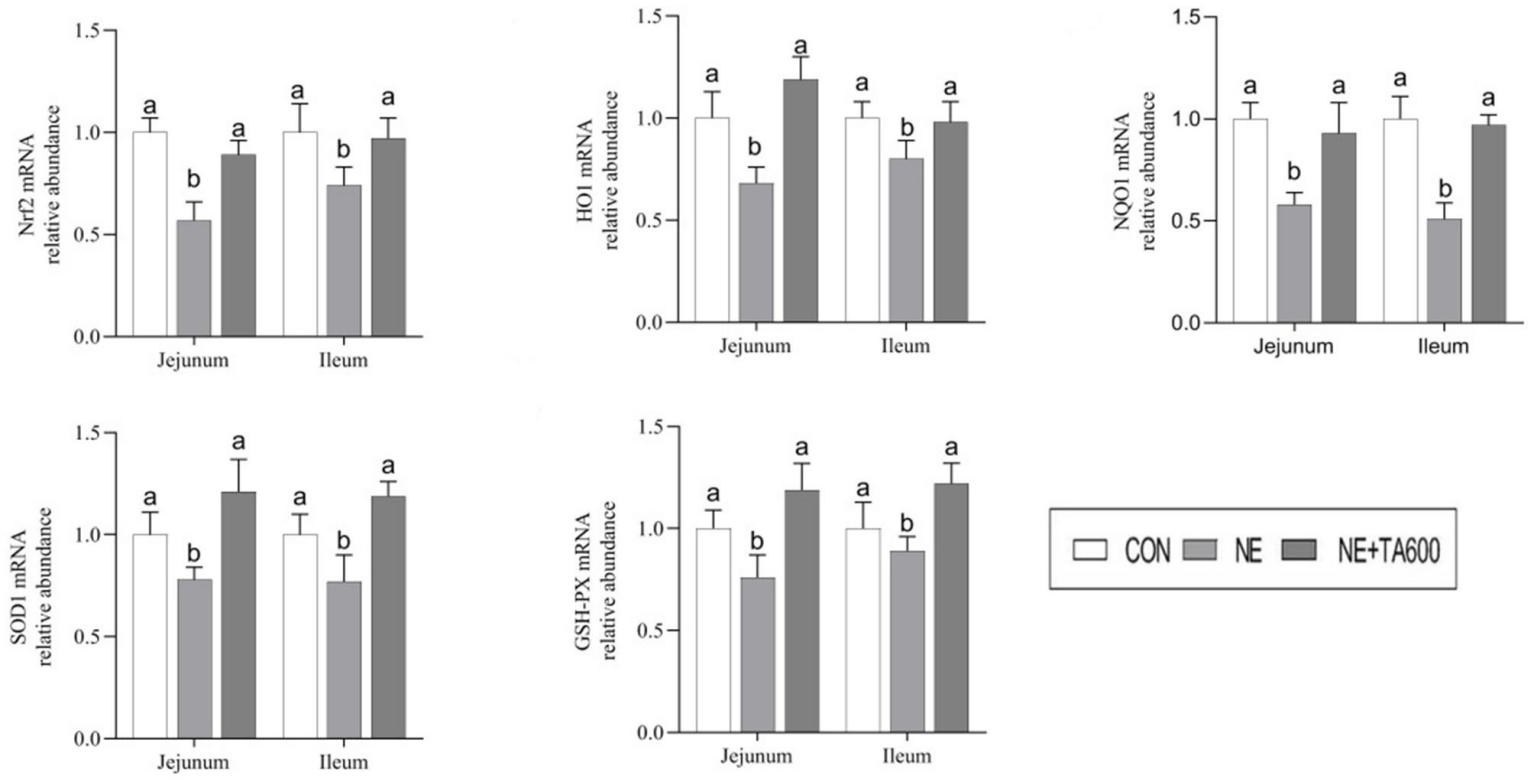
Effects of TA on the mRNA abundance of Nrf2 pathway and antioxidant enzymes in the jejunum and ileum of subclinical NE broilers. Values are means with their standard errors represented by vertical bars. ^a, b^Means within a row with different letters differ significantly (*p* < 0.05). Nrf2, nuclear factor erythroid 2-related factor 2; NQO1, NAD(P)H quinone dehydrogenase 1; HO1, heme oxygenase 1; SOD1, superoxide dismutase 1; GSH-PX, glutathione peroxidase.

As exhibited in Figure 3, the results showed that subclinical NE challenge downregulated the jejunal and ileal mRNA expression of Nrf2, HO1, NQO1, SOD1, and GSH-PX as compared with control group, while the inclusion of TA upregulated (*p* < 0.05) the mRNA abundance of these genes.

**Figure 4:**
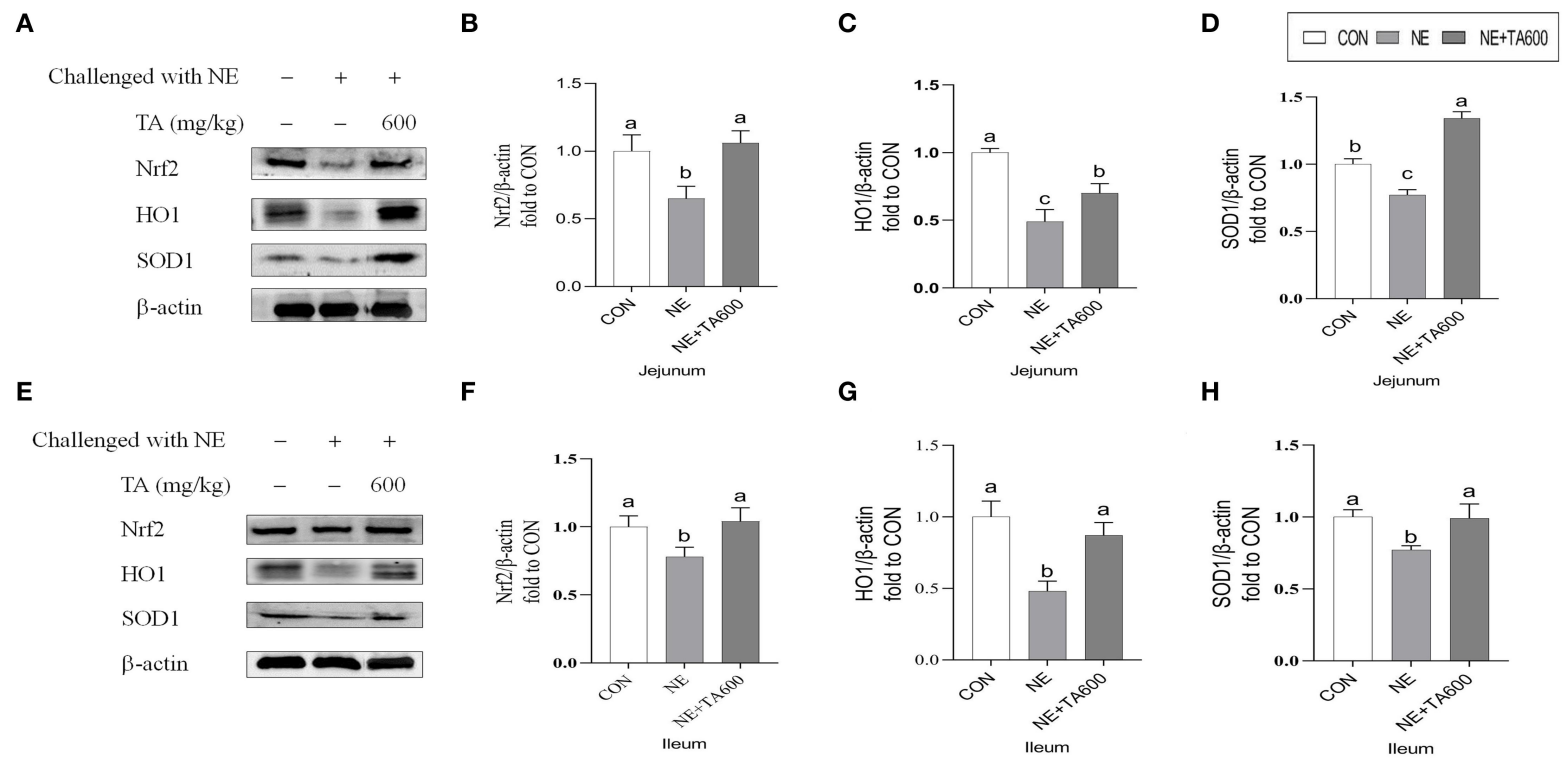
Effects of TA on the relative protein abundance of Nrf2 and SOD1 in the jejunum and ileum of subclinical NE broilers. **(A–D)** The protein abundance of Nrf2, HO1, and SOD1 in the jejunum; **(E–H)** The protein abundance of Nrf2, HO1, and SOD1 in the ileum. Values are means with their standard errors represented by vertical bars. ^a−*c*^Means within a row with different letters differ significantly (*p* < 0.05). Nrf2, nuclear factor erythroid 2-related factor 2; HO1, hemeoxygenase 1; SOD1, superoxide dismutase 1.

Western blot results revealed that the jejunal and ileal nuclear translocation level of Nrf2, and protein abundance of HO1, and SOD1 of subclinical NE infected birds fed with TA was higher (*p* < 0.05) than those in the NE group (Figure 4).

### Expression of Genes Related to Mucosal Repair Factors

**Figure 5:**
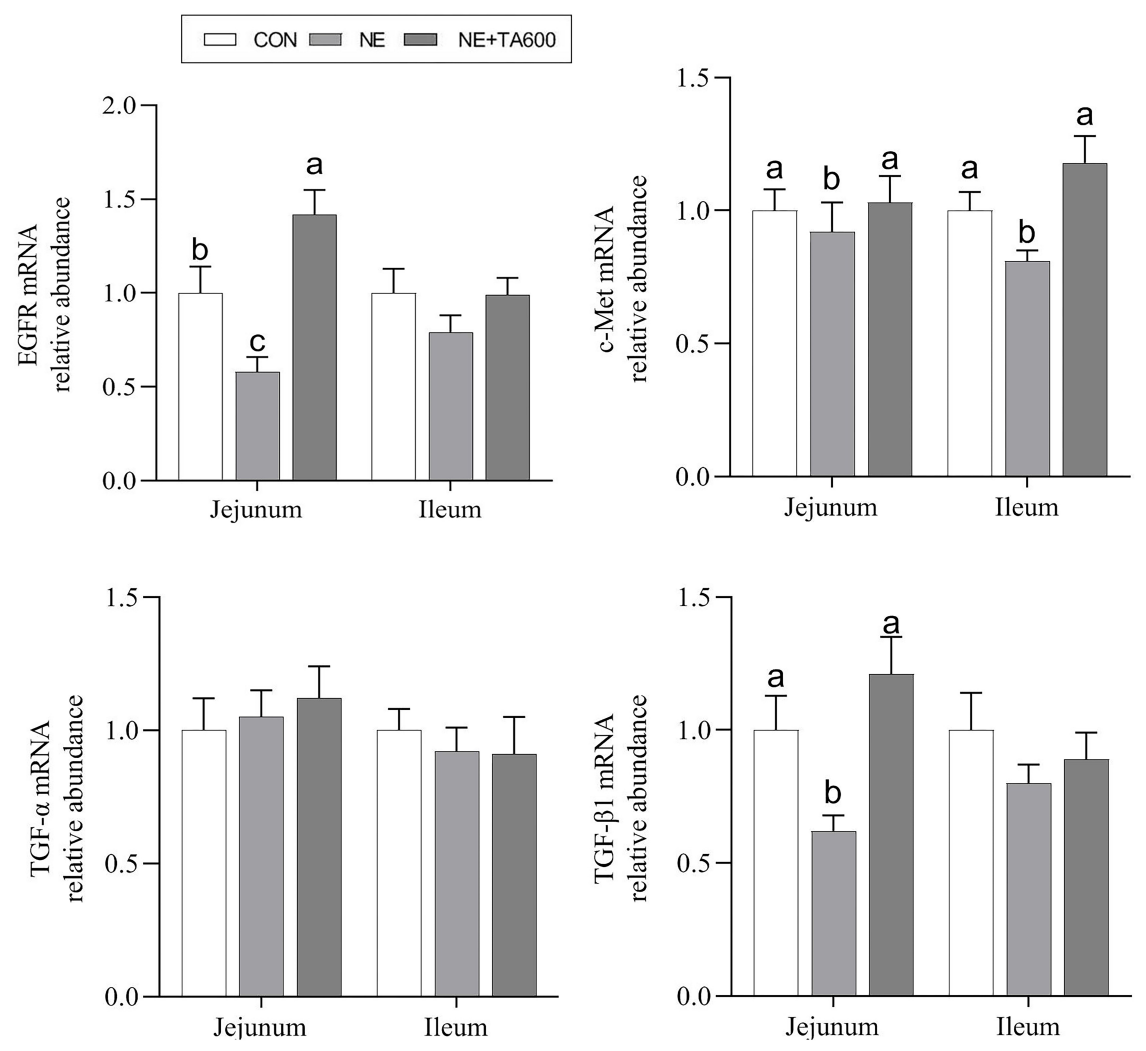
Effects of TA on the mRNA abundance of intestinal mucosal repair factor of subclinical NE broilers. Values are means with their standard errors represented by vertical bars. ^a−*c*^Means within a row with different letters differ significantly (*p* < 0.05). EGFR, epidermal growth factor receptor; c-Met, c-mesenchymal epithelial transition factor; TGF-α, transforming growth factor-alpha; TGF-β1, transforming growth factor-beta 1.

Figure 5 presents the results of jejunal and ileal mucosal repair factors gene expression. Dietary inclusion of TA elevated downregulated (*p* < 0.05) mRNA abundance of jejunal and ileal c-Met, and jejunal EGFR and TGF-β1 in subclinical NE infected birds. The ileal EGFR and TGF-β1, and jejunal and ileal TGF-α did not differ (*p* > 0.05) among the groups.

## Discussion

Necrotic enteritis caused by *C. perfringens* destroys the intestinal barrier integrity and leads to intestinal dysfunction in chickens, resulting in a decrease in growth performance (21). NE annually costs up to six billion dollars in the poultry industry (3). Previous studies reported that subclinical NE infection could cause more serious economic loss than clinical NE infection due to mild intestinal damage, poor digestion, and further poor growth performance (22, 23). The results of our study revealed that NE infection led to a severe decline in the final BW and ADG, but an increase in F/G of broilers compared with controls, while TA administration at 600 mg/kg reversely improved the growth performance of NE birds. Accordingly, the VH and VH/CD were improved by TA during the NE challenge. This may be attributed to the previously reported beneficial effects of TA in increasing nutrient digestibility (24), gastroprotector activity (25), antimicrobial (13, 26), and anti-inflammatory (27–29) activities. However, the results of this study revealed that ADG and F/G were not altered by TA supplementation before NE infection. Similar with that, a recent study investigated the effects of different concentrations of TA on the growth performance of broilers and observed that TA inclusion had no distinct effect on ADG, BW, and F/G (17). It was reported that the growth performance of broilers was not affected by the inclusion of essential oils consisting of menthol and anethole (30). Additionally, the experimental conditions, hygiene, animal age, diet type, and altered microbiota may also affect the performance response of broilers to TA (31, 32). Taken together, the effects of TA on growth performance of broilers under normal feeding conditions and subclinical NE challenge require to be further characterized.

Necrotic enteritis infection is usually accompanied by intestinal lesions, intestinal inflammation, and intestinal oxidative stress (4, 5). Rochat et al. (33) also indicated that bacterial infection could cause gastrointestinal inflammation and further lead to oxidative stress. Therefore, we conducted the current study to investigate whether TA could ameliorate subclinical NE in broilers by enhancing the intestinal antioxidant status and intestinal mucous repair factor expression.

The anti-inflammatory activity of *Foeniculum vulgare* essential oil (81.08% TA) against acetic acid-induced colitis in rats has been reported (27). TA, one of the main active constituents present in essential oils of plants, such as *Syzygium anisatum, F. vulgare, Coriandrum sativum*, and star anise, has been shown the anti-inflammatory activity by regulation of Th17/Treg function in the mouse model of lipopolysaccharide-induced acute lung injury (28). In another study, TA showed a hepatoprotective effect against hepatic ischemia/reperfusion injury *via* inhibition of toll-like receptor activation (34). Additionally, treatment with TA exerted a protective effect on the hepatotoxicity induced by acetaminophen *via* down-regulating the pro-inflammatory mediators (35). These data indicated that TA may ameliorate intestinal injury due to its anti-inflammatory activity. The present study shows that TA inclusion reduces intestinal damage induced by *C. perfringens* in the experimental model of subclinical NE through the increase of serum and intestinal antioxidant enzymes activities. Previously, TA was reported to play an crucial role in the maintenance of the redox balance through either decreasing ROS levels (36) or enhancing the activities of cellular antioxidant enzymes, such as SOD and GSH-PX (37). Oxidative stress leads to the release of intracellular cytokines and further causes systemic and chronic inflammation (6, 38). Changes in antioxidant enzyme activities can result in oxidative stress. It is well-known that the levels of antioxidant defense can be reflected by the determination of antioxidant activities of T-SOD, T-AOC GSH-PX, and MDA concentration. SOD and GSH-PX are vital intracellular antioxidant enzymes responsible for the antioxidant defense system *via* converting oxygen radicals to hydrogen peroxide (39). Overall antioxidant defense capacity can be determined by T-AOC. In this study, TA inclusion enhanced serum and intestinal SOD activities as well as the jejunal mRNA and protein levels of SOD1 in subclinical NE-infected birds. Furthermore, TA interventions reversely elevated the serum and jejunal activities of T-AOC and GSH-PX, and mRNA abundance of GSH-PX in the jejunum and ileum of birds infected with subclinical NE. These results indicated that the increased expression and activities of antioxidant enzymes may be the mechanism of action by which TA alleviates the intestinal damage caused by subclinical NE. Similarly, Chaudhari et al. (40) observed that TA exhibited the *in vitro* free radicals scavenging activity and the inhibiting capacity of lipid oxidation in stored maize samples, confirming the antioxidant activity of TA in preserving maize samples. TA reduced oxidative stress of *in vitro* primordial follicles and bovine embryos development *by* decreasing the production of ROS and regulating the redox balance (8, 41). TA also prevented hydrogen peroxide-induced collagen metabolism alterations and apoptosis in human skin fibroblasts, proving that TA may be an effective therapeutic agent for oxidative stress-related skin diseases (10). Besides that, the antioxidant potential of TA has been widely reported (25, 42, 43). The antioxidant activity of TA may be attributed to the conjugated double bonds and phenol group in its chemical structure, which has high reactivity with peroxyl radicals (10, 25, 44, 45). On the other hand, TA increased jejunal and ileal mRNA and protein expression of Nrf2, and downstream target molecules. Nrf2 is a principal transcription factor exerting an antioxidant role and maintaining cellular redox balance (46). Upon activation, Nrf2 moves into cell nucleus after releasing from Keap 1, where it is combined to the antioxidant response element to activate transcription of antioxidant genes. Similarly, it has been demonstrated that star anise oil could reduce the oxidative stress of birds during subclinical *Escherichia coli* challenges through upregulation of the Nrf2 signaling pathway (47). In addition, we found that NE infection resulted in vacuolization and swelling in the mitochondria, while TA administration improved that. Many lines of evidence indicate that mitochondria play a key role in preventing oxidative damage (48). Therefore, further research on the protective effects of TA on the subclinical NE-induced mitochondria dysfunction requires to be characterized.

Previous studies have widely reported that growth factors, including EGFR, TGF-β, and TGF-α, had positive impacts on epidermal repair and regeneration, inflammation, and proliferation (49–51). Alterations in the endogenous growth factors status are also correlated with poor clinical prognosis (52). In addition, the hepatocyte growth factor (HGF)/c-MeT signaling system may contribute to cell mobilization, tissue regeneration, and repair (53). Our results showed that dietary inclusion of TA elevated down-regulated mRNA abundance of jejunal and ileal c-Met, and jejunal EGFR and TGF-β1 in subclinical NE-infected birds. These data indicated that TA may have substantial repair effects on the damaged intestinal mucosa induced by subclinical NE infection. The specific mechanism requires to be further characterized.

This study indicated that TA has promising potential treatment in subclinical NE in broilers. Prior to that, TA has been proven effective in animal and cell experimental models of a variety of diseases. Therefore, it may be worthwhile to further explore the pharmacological effects of TA in intestinal diseases of humans and animals.

## Conclusion

The inclusion of 600 mg/kg of TA may be a promising tool to prevent and control subclinical NE by increasing intestinal antioxidant status and intestinal mucosal repair factor expression in broilers. The mechanisms by which TA exerts its antioxidant activity may be attributed to the activation of Nrf2 signaling pathway. Taken together, TA may be an effective agent to prevent and treat NE in poultry industry.

## Data Availability Statement

The original contributions presented in the study are included in the article/Supplementary Material, further inquiries can be directed to the corresponding author/s.

## Author Contributions

CY contributed to conceptualization, methodology, and writing—original draft. YT and QL contributed to investigation and supervision. TW contributed to supervision, project administration, and funding acquisition. ZY contributed to visualization, funding acquisition, writing, reviewing, and editing the manuscript. All authors contributed to the article and approved the submitted version.

## Funding

This work was supported by the National Key Research and Development Program of China (Grant No. 2018YFD0501101).

## Conflict of Interest

The authors declare that the research was conducted in the absence of any commercial or financial relationships that could be construed as a potential conflict of interest.

## Publisher's Note

All claims expressed in this article are solely those of the authors and do not necessarily represent those of their affiliated organizations, or those of the publisher, the editors and the reviewers. Any product that may be evaluated in this article, or claim that may be made by its manufacturer, is not guaranteed or endorsed by the publisher.
