# Supplementary material for: Trans-anethole Ameliorates Intestinal Injury Through Activation of Nrf2 Signaling Pathway in Subclinical Necrotic Enteritis-Induced Broilers
Source: Front Vet Sci. 2022 Apr 18;9:877066. doi: 10.3389/fvets.2022.877066 (PMC9062583; doi:10.3389/fvets.2022.877066)
Supplement: Supplementary file 2 [file Table_2.DOCX]

**Supplementary Table 2**. Gene-specific primers sequences for quantitative real-time PCR

| Gene name^1^ | GenBank^2^ | Primer sequence^3^ (5'→3') | Length |
| --- | --- | --- | --- |
| Nrf2 | NM_205117.1 | GGGCAAGGCGTGAAGTTTTT | 116 |
|  |  | GGCTTTCTCCCGCTCTTTCT |  |
| NQO1 | NM_001277619.1 | CGCACCCTGAGAAAACCTCT | 166 |
|  |  | AAGCACTCGGGGTTCTTGAG |  |
| HO-1 | NM_205344.1 | AGCTTCGCACAAGGAGTGTT | 106 |
|  |  | GGAGAGGTGGTCAGCATGTC |  |
| SOD1 | NM_205064.1 | GGCAATGTGACTGCAAAGGG | 133 |
|  |  | CCCCTCTACCCAGGTCATCA |  |
| GSH-PX | NM_001277853.2 | GTTCGAGAAGTGCGAGGTGA | 116 |
|  |  | TGTACTGCGGGTTGGTCATC |  |
| EGFR | NM_205497.2 | CCCCGCAAGTGAAATCTCCT | 121 |
|  |  | ACGGCTGTCTGCATCAATCA |  |
| c-Met | NM_205212.2 | AGCGAGAGCGGTTGCC | 99 |
|  |  | TTGTCAGGTTTAATCGCGGC |  |
| TGF-α | AY605246.1 | CCCTGGAGAACACAACGTCA | 110 |
|  |  | CGTCCCATGGAAGCAGAACT |  |
| TGF-β1 | NM_001318456.1 | TGGATCCACGAACCCAAAGG | 189 |
|  |  | CCGGCCCACGTAGTAAATGA |  |

^1^Nrf2, nuclear factor erythroid 2-related factor 2; NQO1, NAD(P)H quinone dehydrogenase 1; HO-1, heme oxygenase 1; SOD1, superoxide dismutase 1; GSH-PX, glutathione peroxidase; EGFR, epidermal growth factor receptor; c-Met, c-mesenchymal epithelial transition factor; TGF-α, transforming growth factor alpha; TGF-β1, transforming growth factor beta 1.

^2^GenBank Accession Number.

^3^Shown as the forward primer then the reverse primer.
