# Supplementary figures and images for: Trans-anethole Ameliorates Intestinal Injury Through Activation of Nrf2 Signaling Pathway in Subclinical Necrotic Enteritis-Induced Broilers
Source: Front Vet Sci. 2022 Apr 18;9:877066. doi: 10.3389/fvets.2022.877066 (PMC9062583; doi:10.3389/fvets.2022.877066)

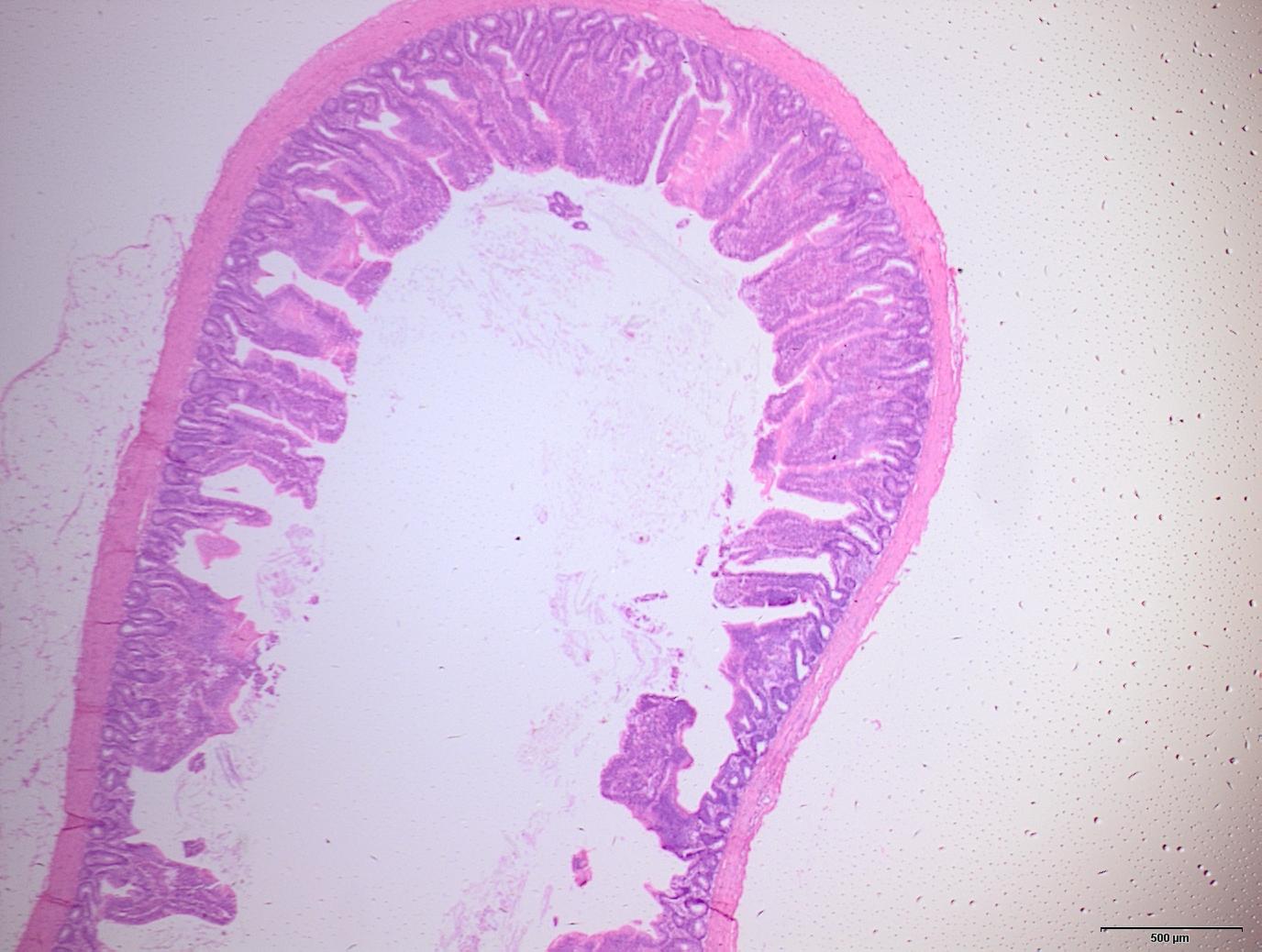

Supplement: Supplementary file 3 [file Data_Sheet_1.ZIP › Data sheet/Hematoxylin-eosin Staining/Ileum/CON group/1.jpg]

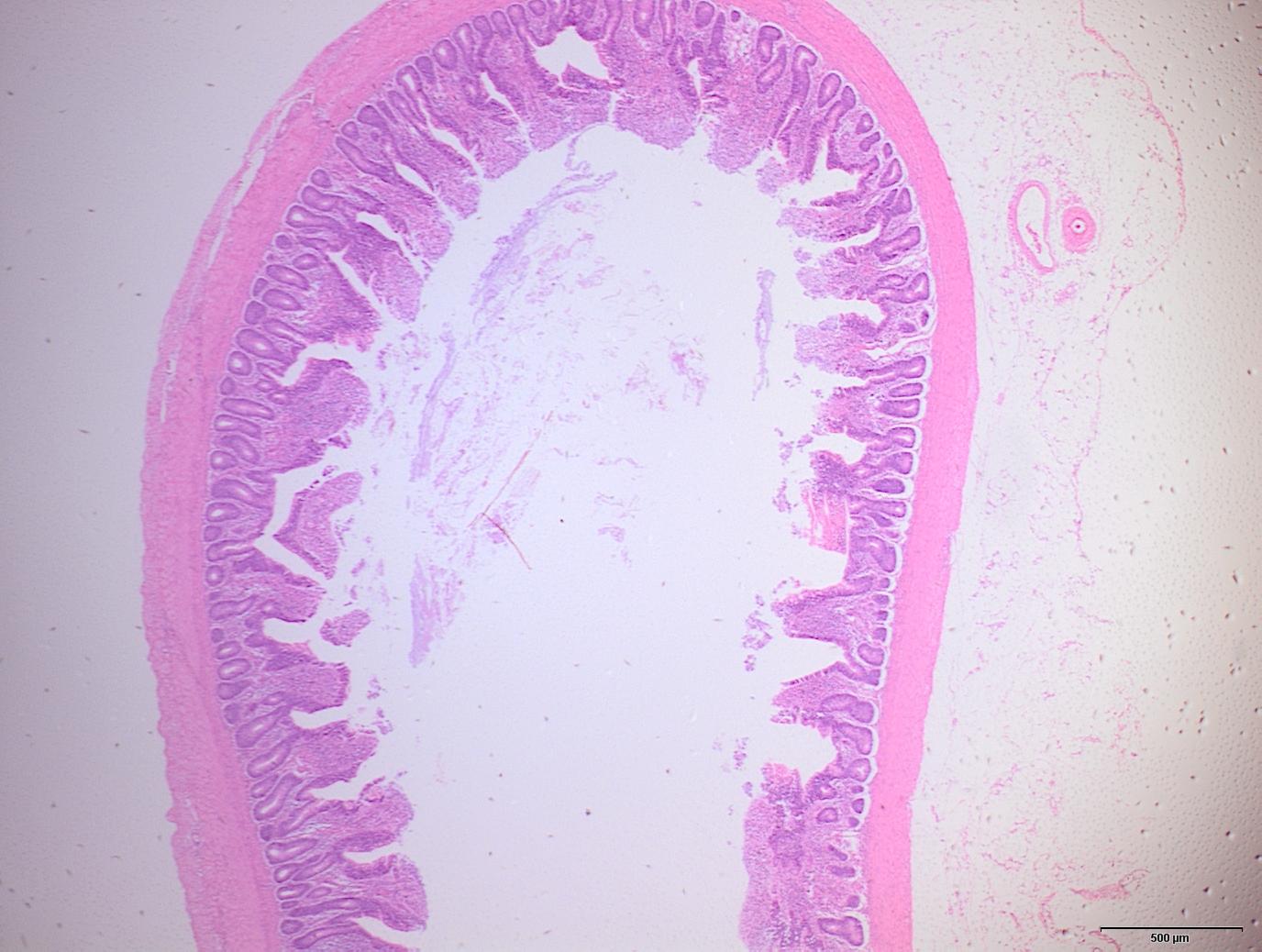

Supplement: Supplementary file 3 [file Data_Sheet_1.ZIP › Data sheet/Hematoxylin-eosin Staining/Ileum/CON group/2.jpg]

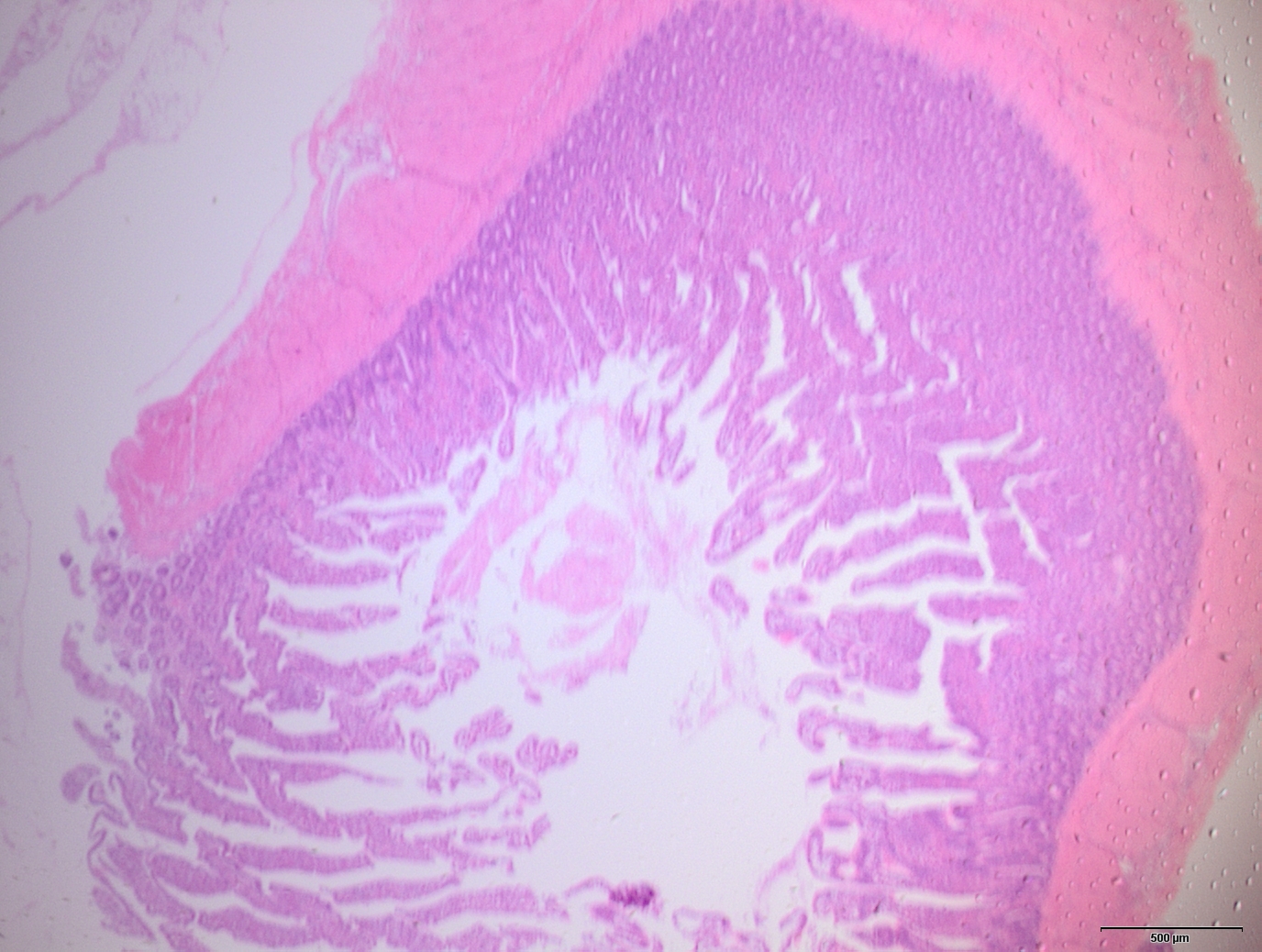

Supplement: Supplementary file 3 [file Data_Sheet_1.ZIP › Data sheet/Hematoxylin-eosin Staining/Ileum/CON group/3.jpg]

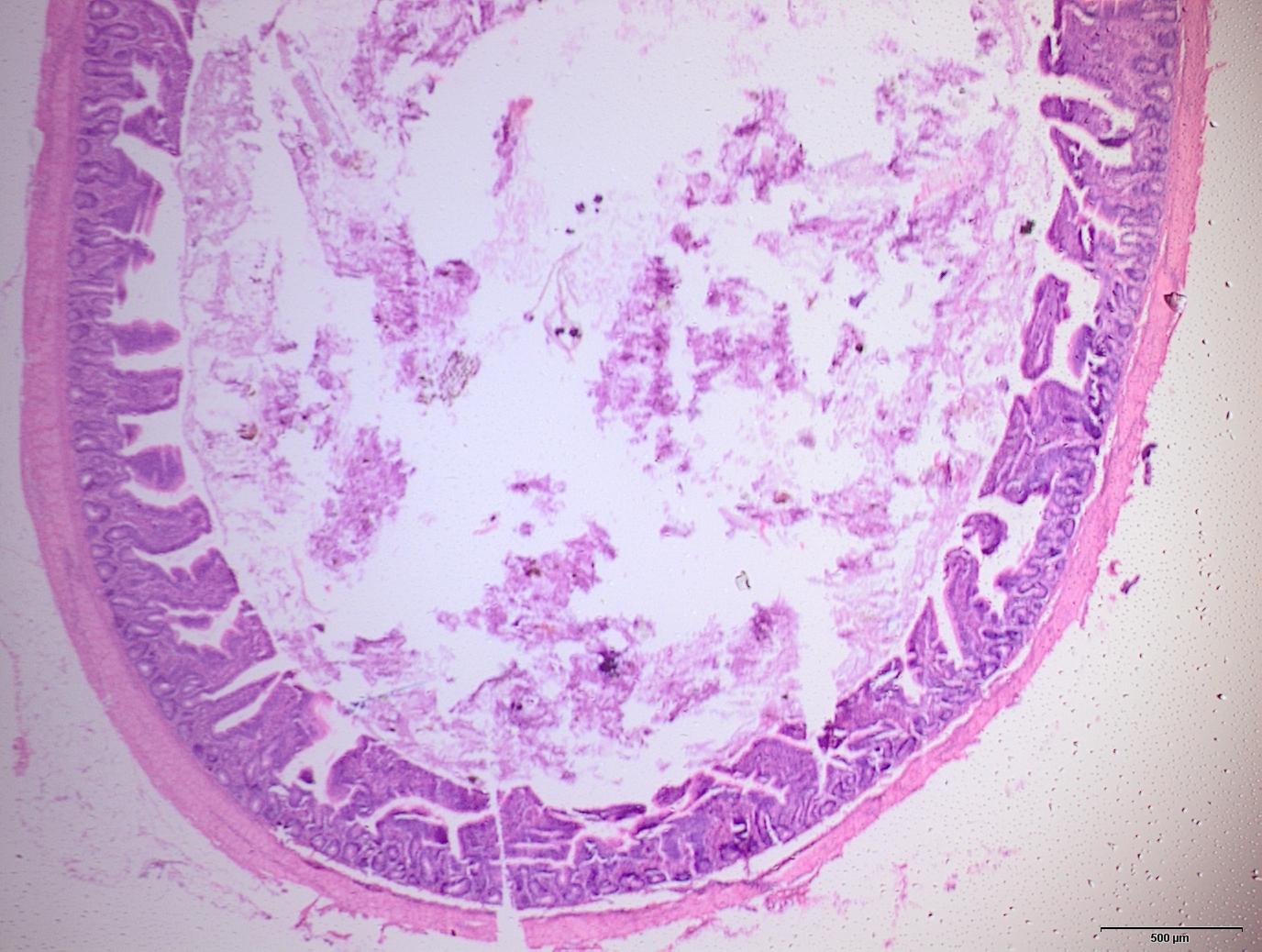

Supplement: Supplementary file 3 [file Data_Sheet_1.ZIP › Data sheet/Hematoxylin-eosin Staining/Ileum/CON group/4.jpg]

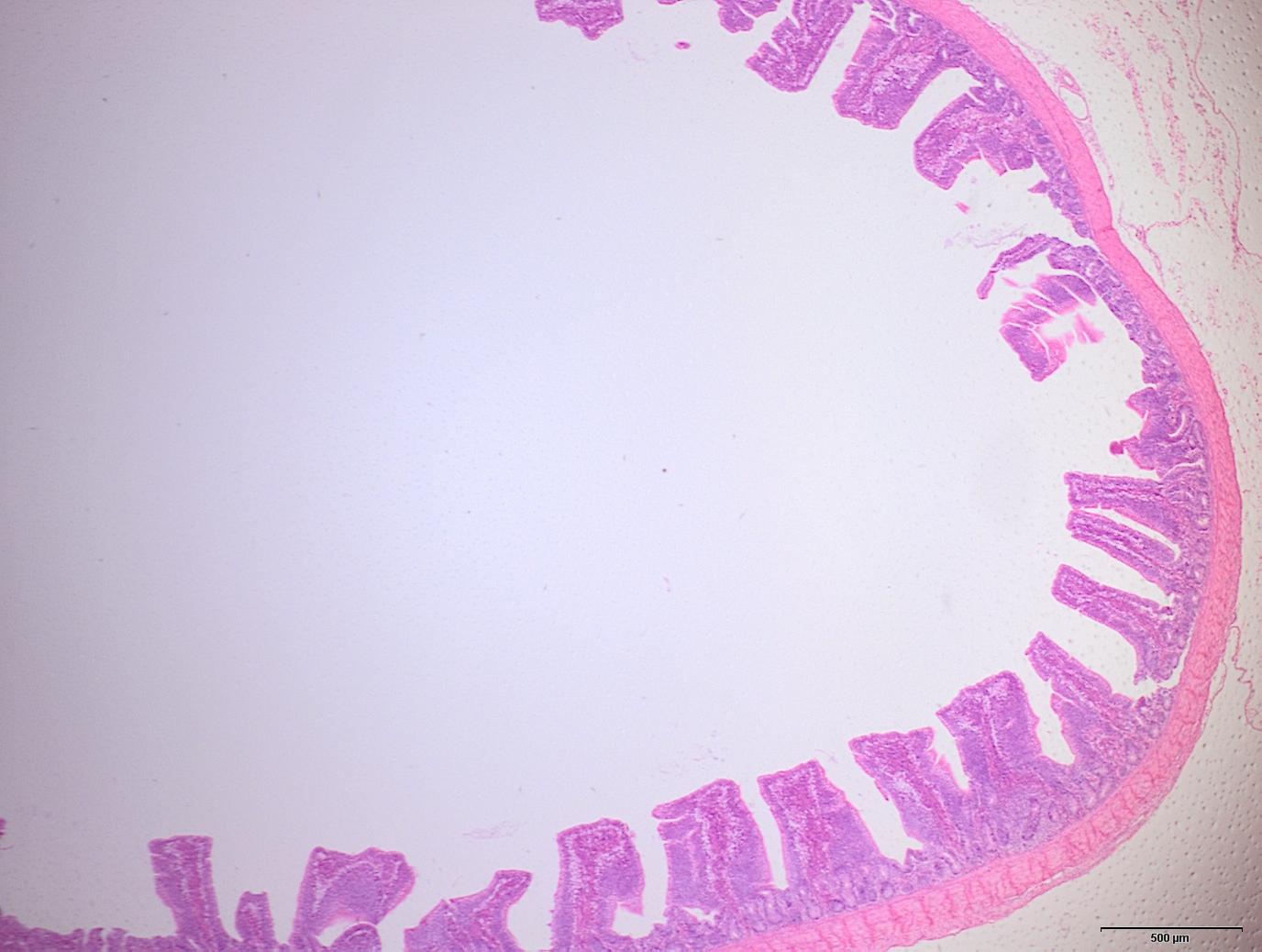

Supplement: Supplementary file 3 [file Data_Sheet_1.ZIP › Data sheet/Hematoxylin-eosin Staining/Ileum/CON group/5.jpg]

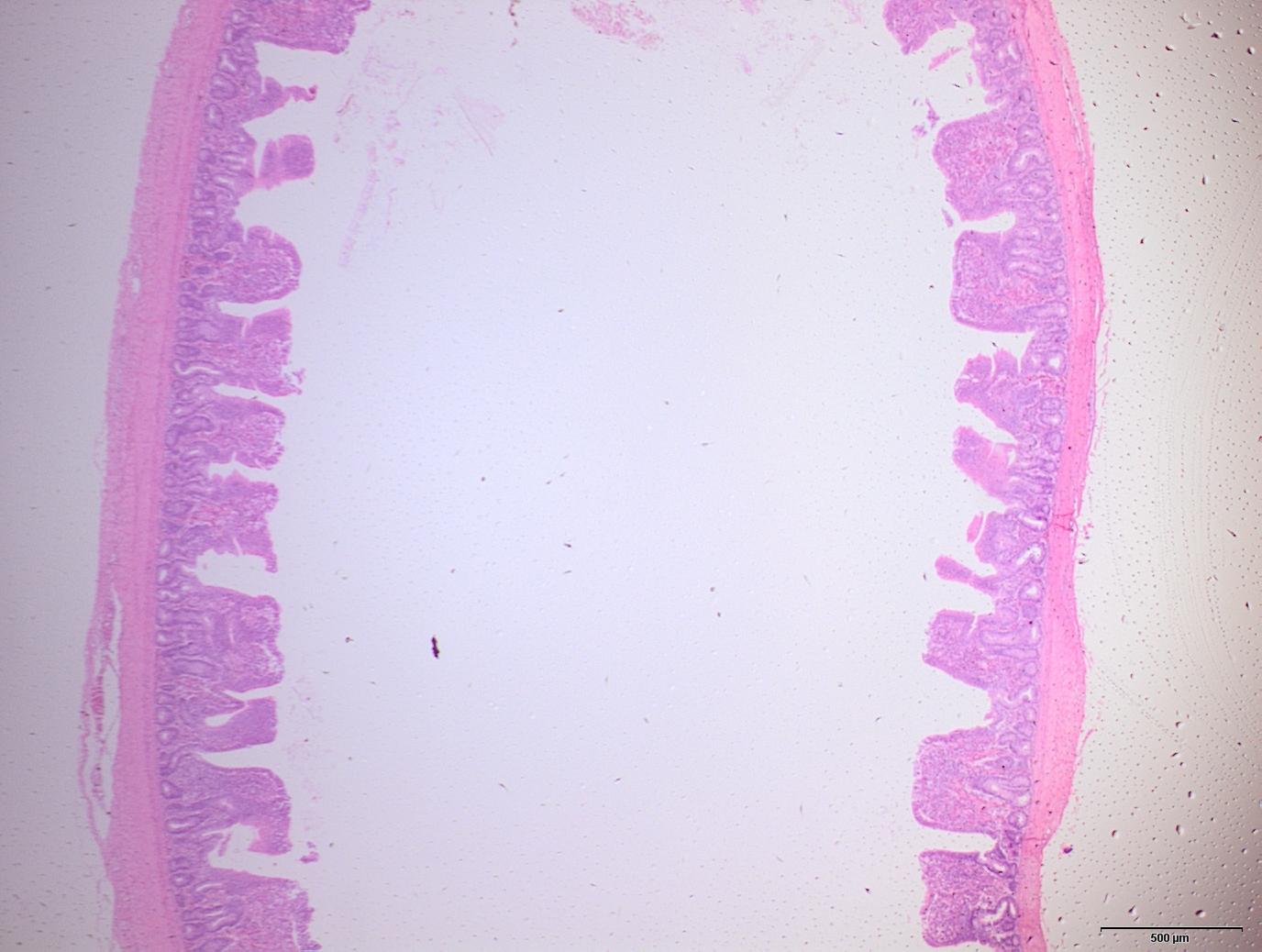

Supplement: Supplementary file 3 [file Data_Sheet_1.ZIP › Data sheet/Hematoxylin-eosin Staining/Ileum/CON group/6.jpg]

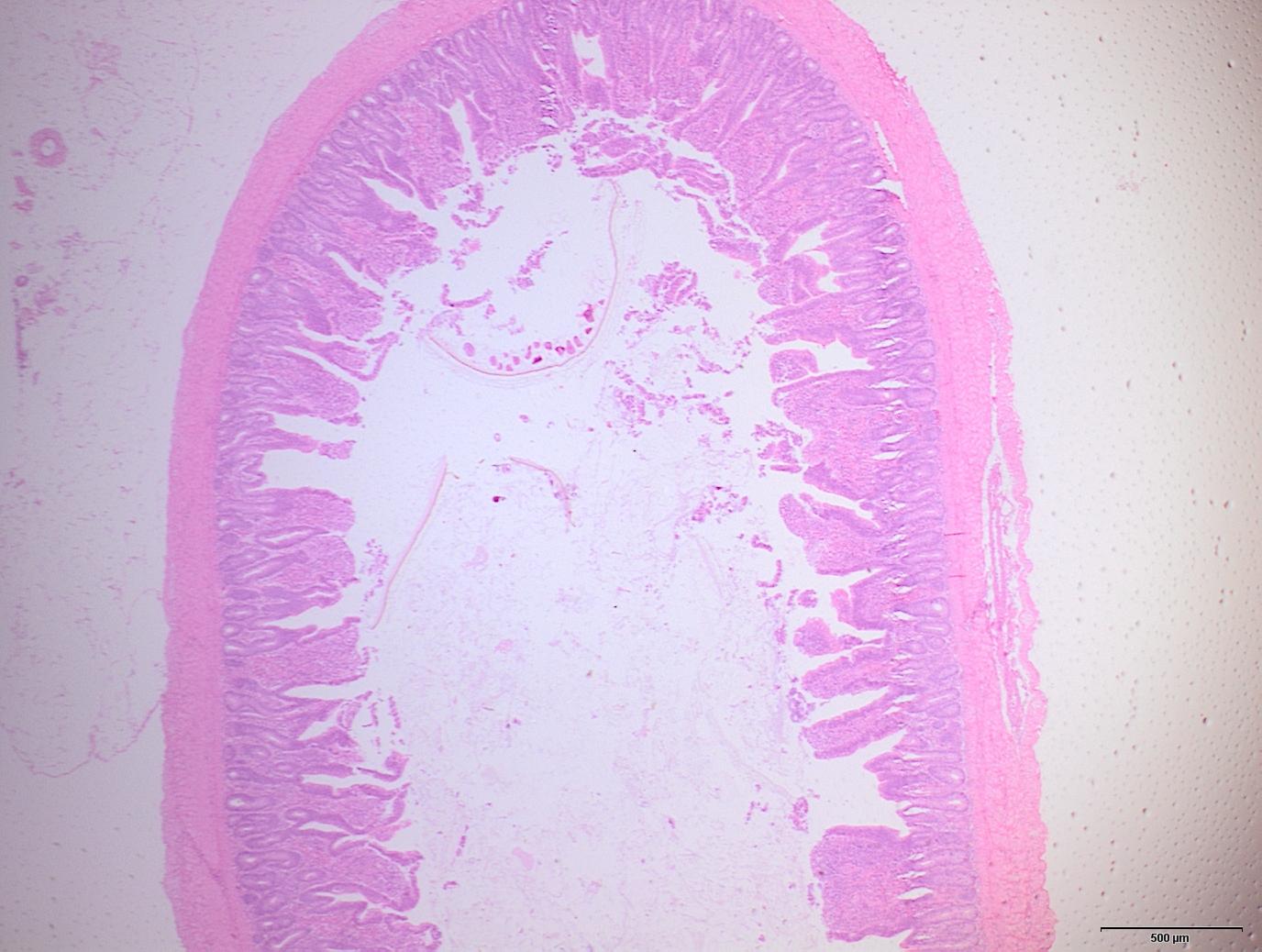

Supplement: Supplementary file 3 [file Data_Sheet_1.ZIP › Data sheet/Hematoxylin-eosin Staining/Ileum/CON group/7.jpg]

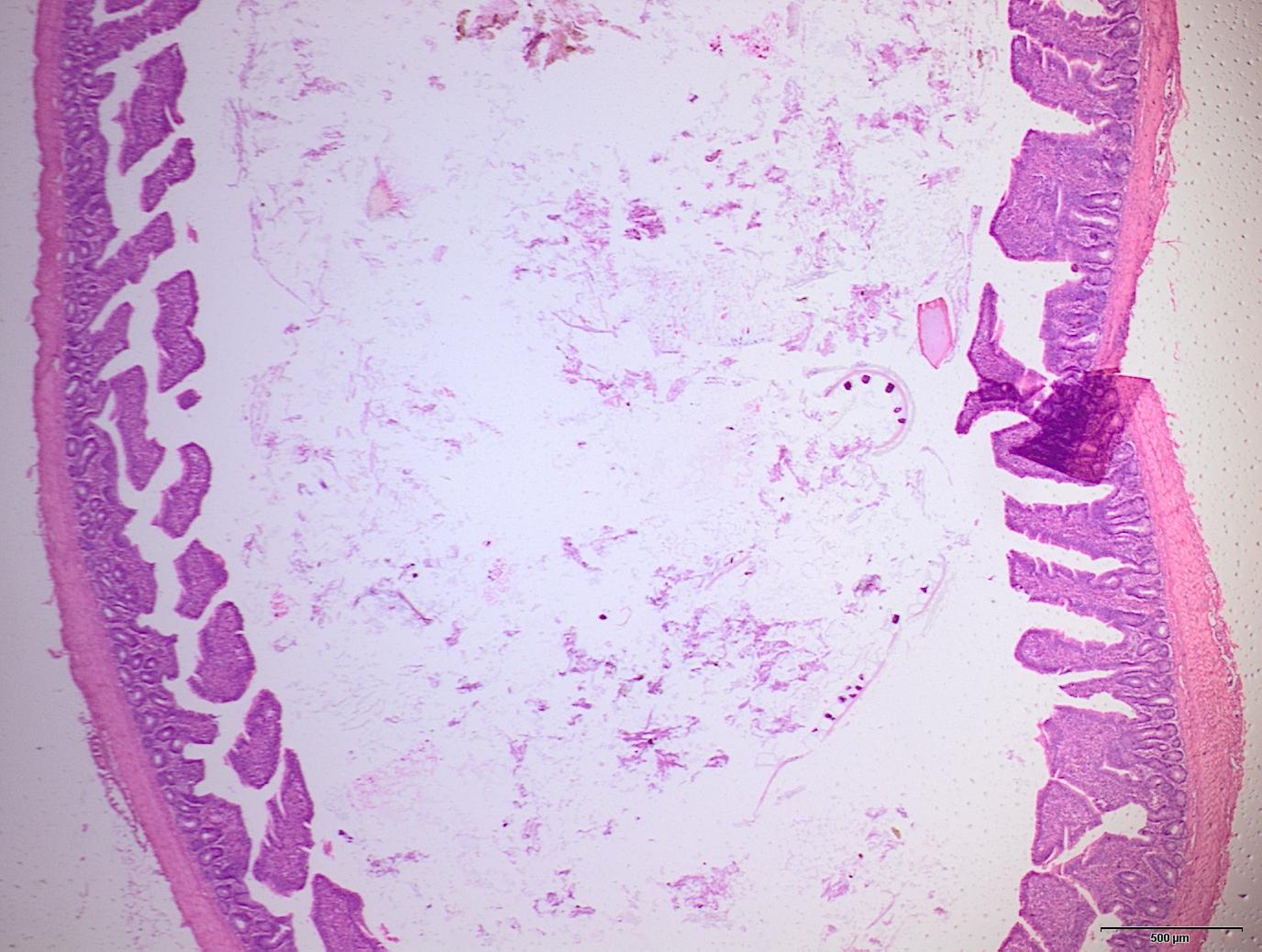

Supplement: Supplementary file 3 [file Data_Sheet_1.ZIP › Data sheet/Hematoxylin-eosin Staining/Ileum/CON group/8.jpg]

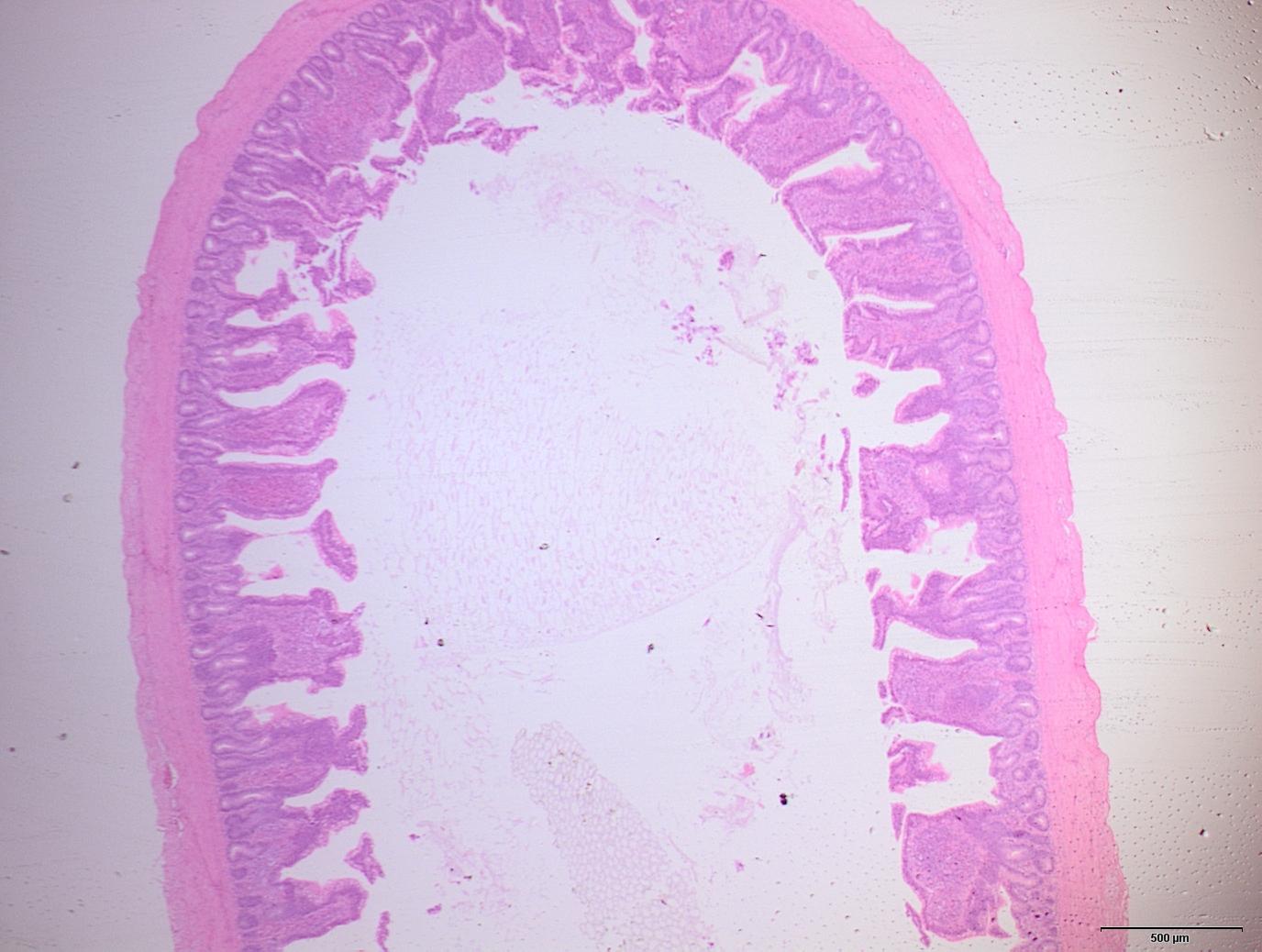

Supplement: Supplementary file 3 [file Data_Sheet_1.ZIP › Data sheet/Hematoxylin-eosin Staining/Ileum/NE group/1.jpg]

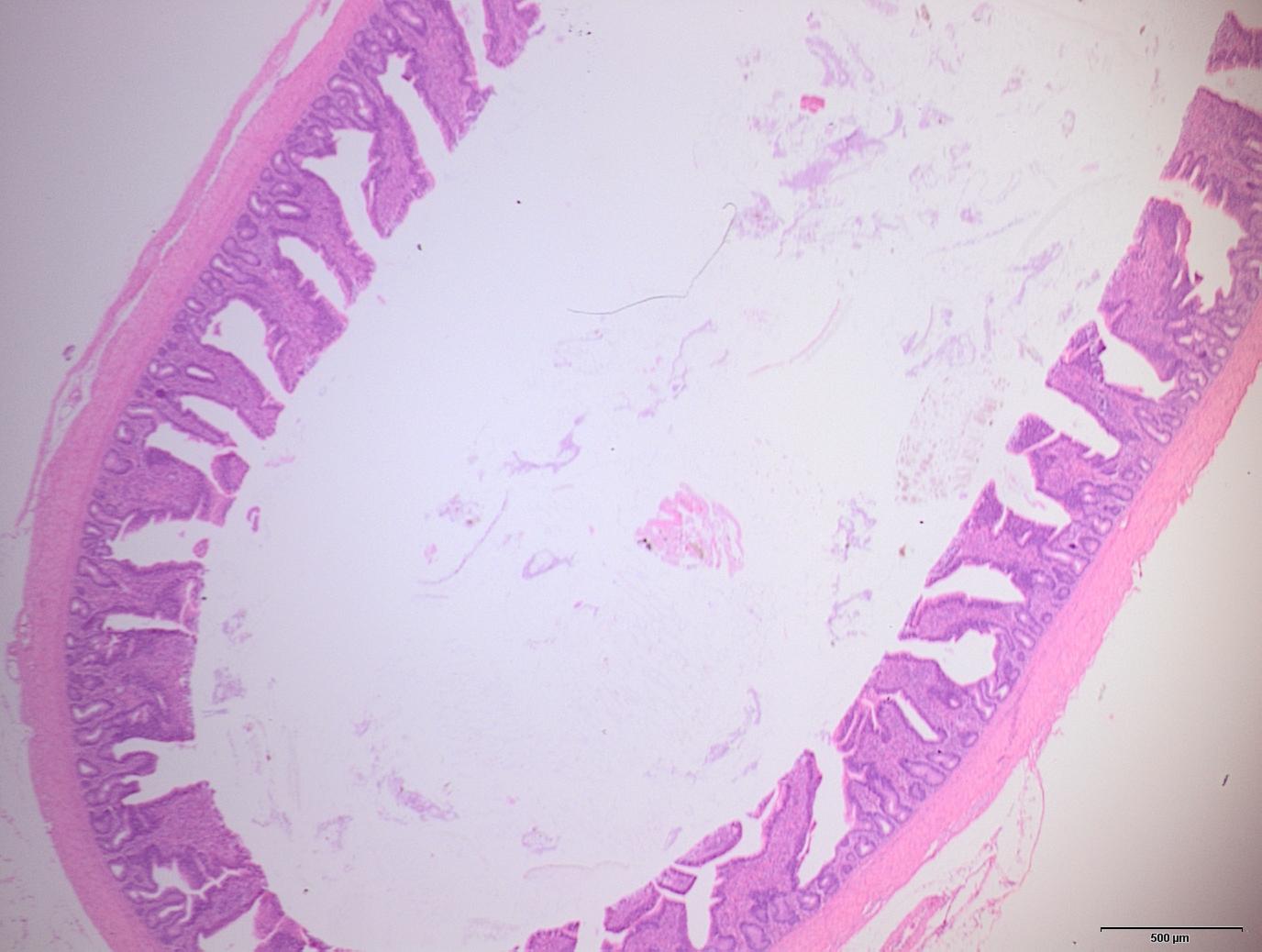

Supplement: Supplementary file 3 [file Data_Sheet_1.ZIP › Data sheet/Hematoxylin-eosin Staining/Ileum/NE group/2.jpg]

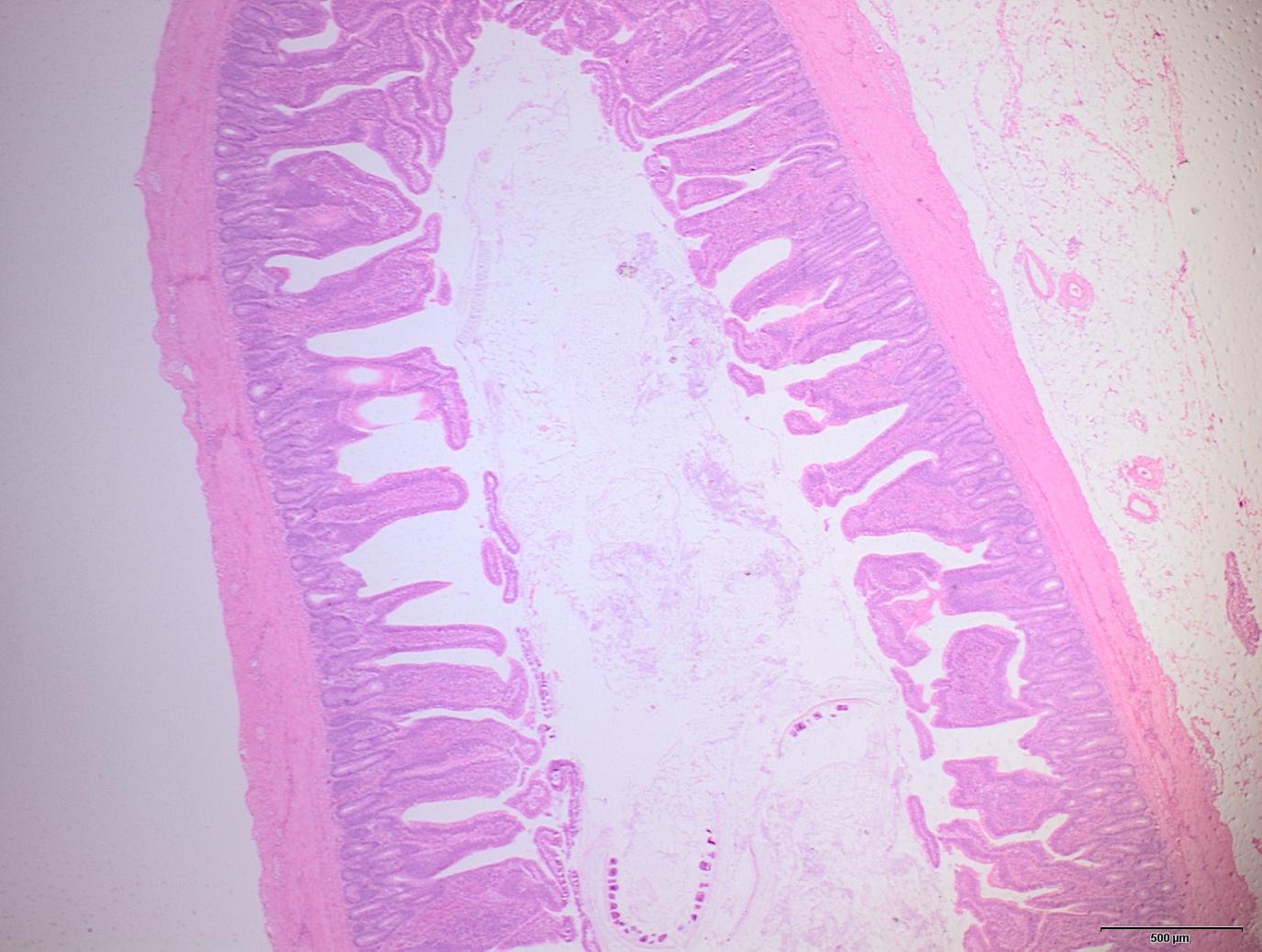

Supplement: Supplementary file 3 [file Data_Sheet_1.ZIP › Data sheet/Hematoxylin-eosin Staining/Ileum/NE group/3.jpg]

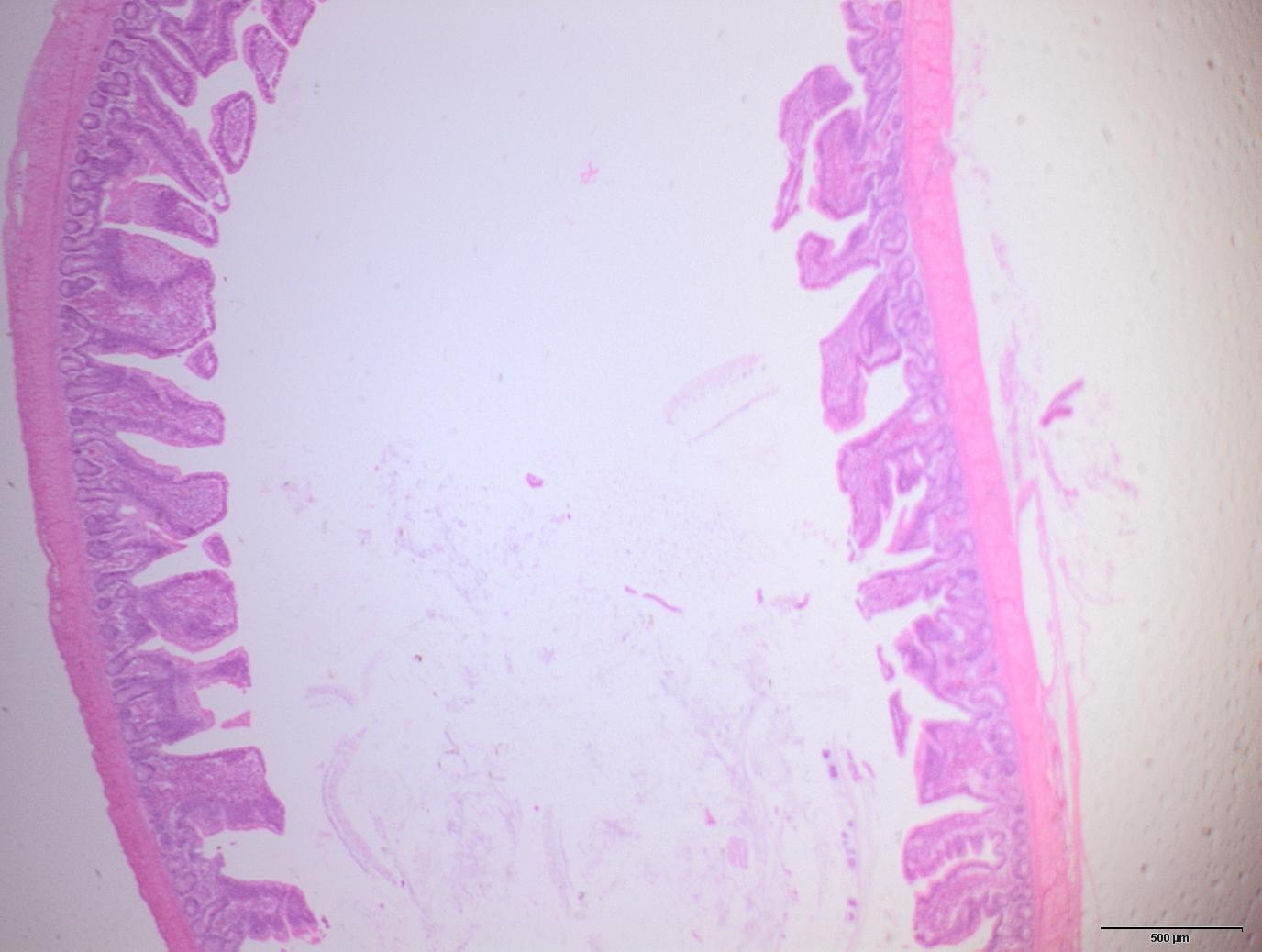

Supplement: Supplementary file 3 [file Data_Sheet_1.ZIP › Data sheet/Hematoxylin-eosin Staining/Ileum/NE group/4.jpg]

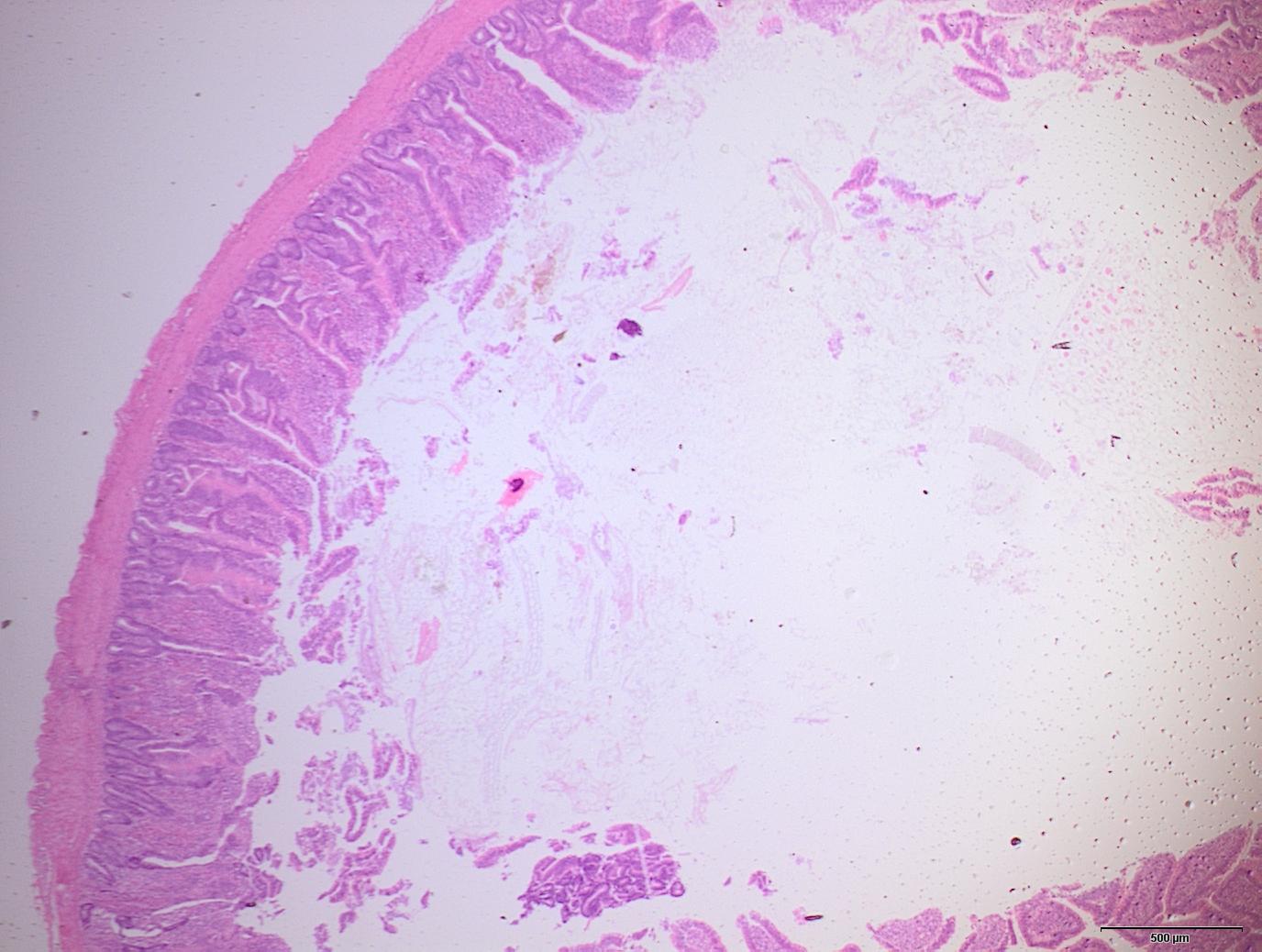

Supplement: Supplementary file 3 [file Data_Sheet_1.ZIP › Data sheet/Hematoxylin-eosin Staining/Ileum/NE group/5.jpg]

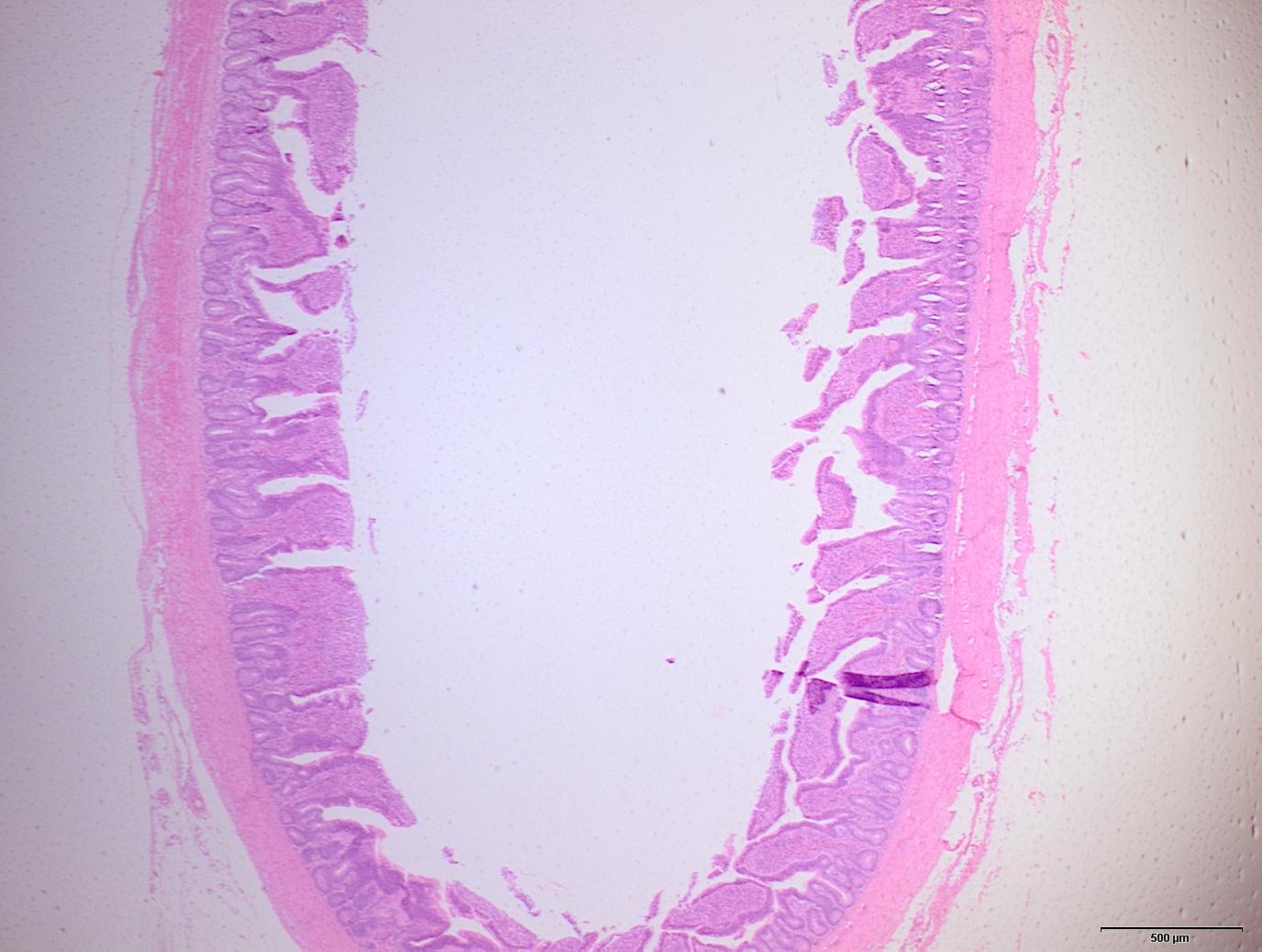

Supplement: Supplementary file 3 [file Data_Sheet_1.ZIP › Data sheet/Hematoxylin-eosin Staining/Ileum/NE group/6.jpg]

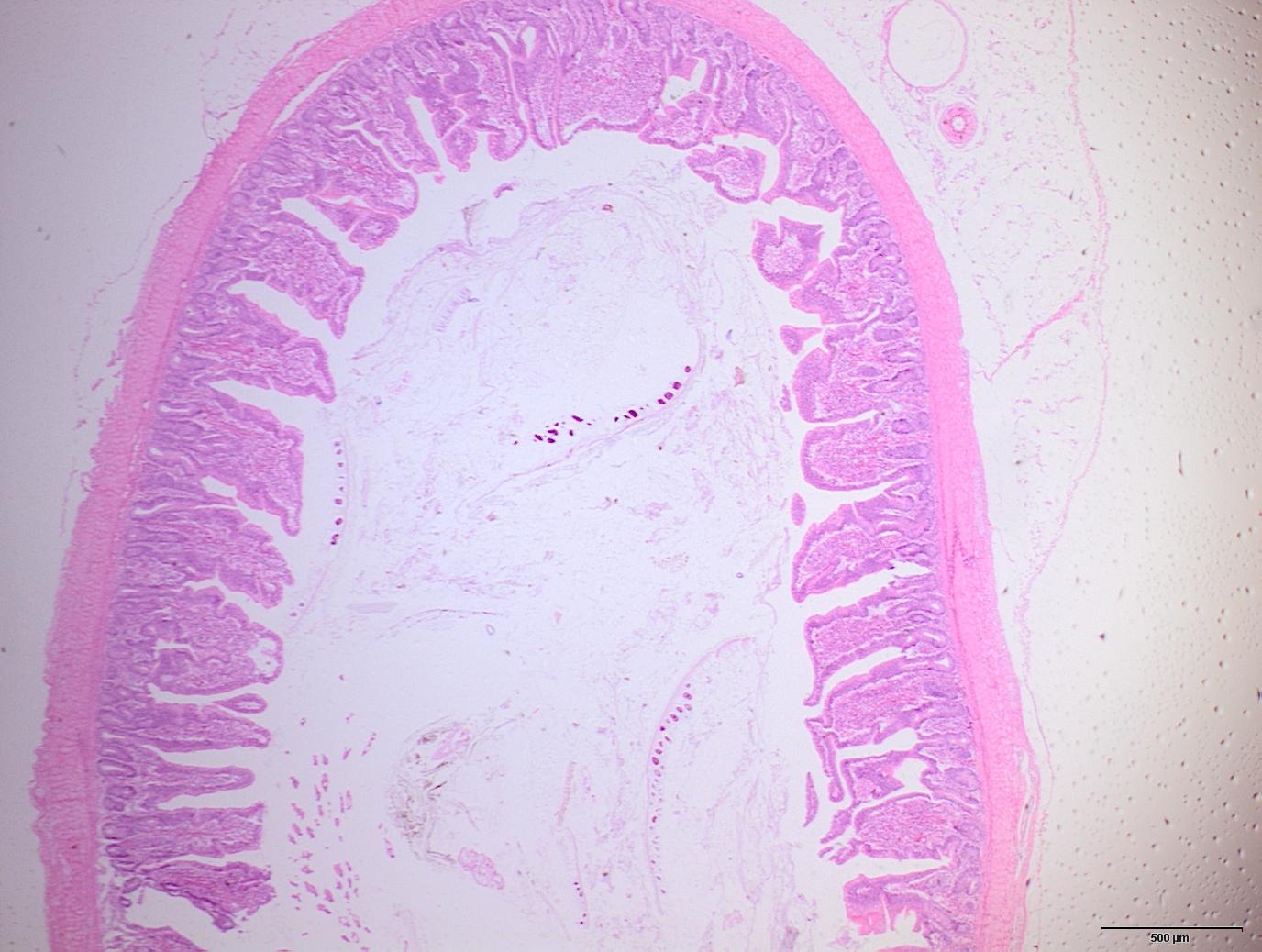

Supplement: Supplementary file 3 [file Data_Sheet_1.ZIP › Data sheet/Hematoxylin-eosin Staining/Ileum/NE group/7.jpg]

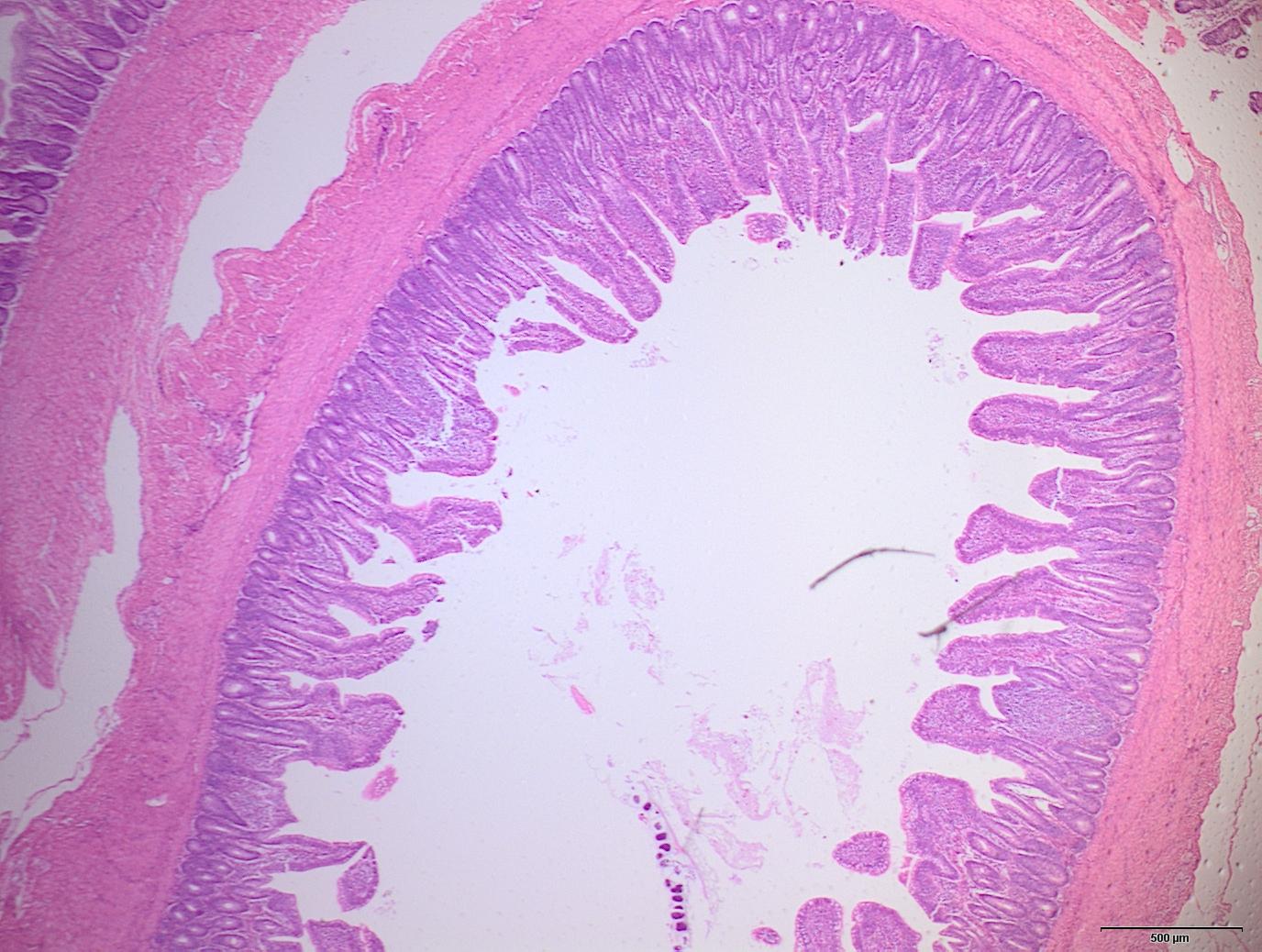

Supplement: Supplementary file 3 [file Data_Sheet_1.ZIP › Data sheet/Hematoxylin-eosin Staining/Ileum/NE group/8.jpg]

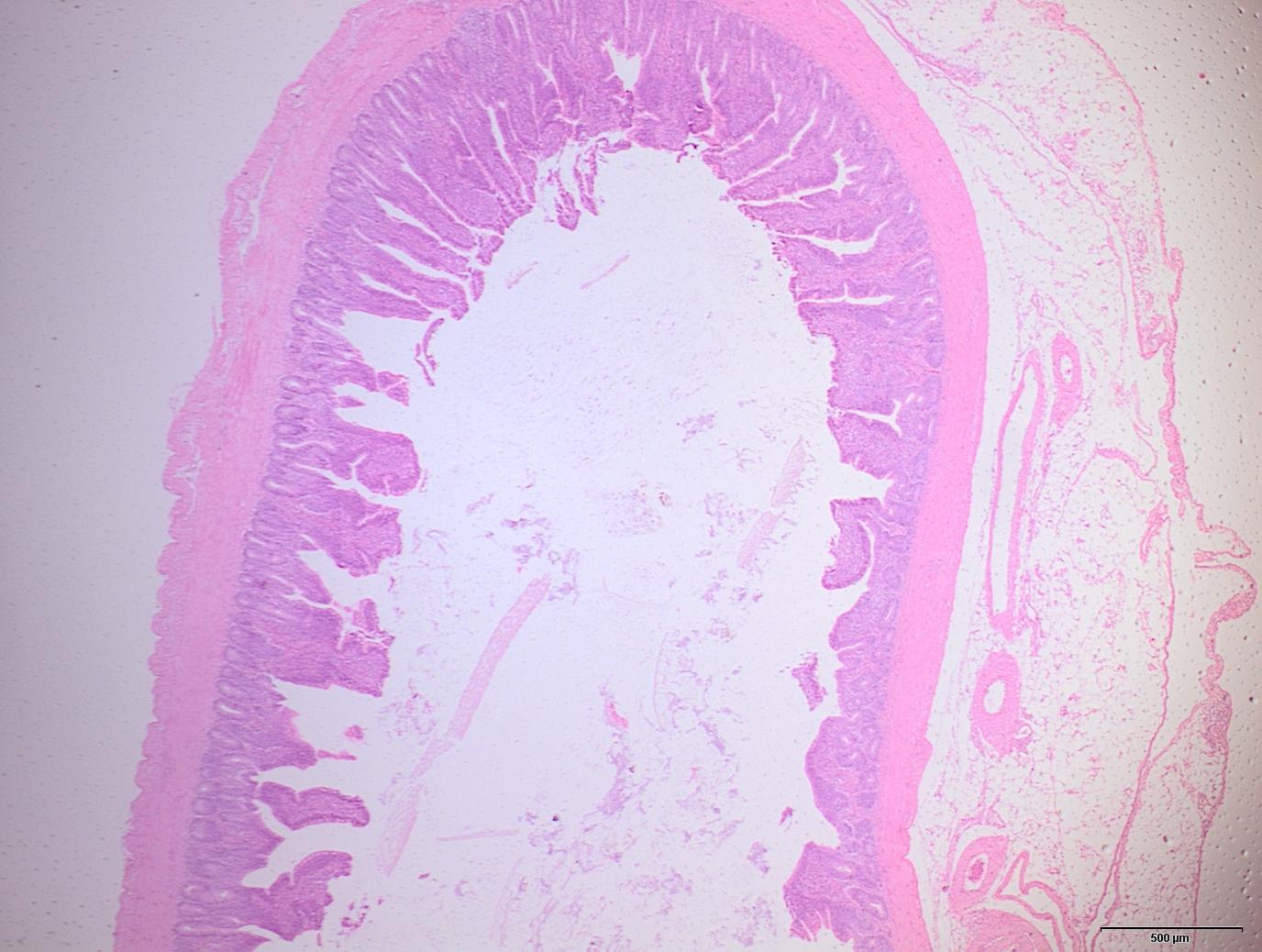

Supplement: Supplementary file 3 [file Data_Sheet_1.ZIP › Data sheet/Hematoxylin-eosin Staining/Ileum/NE+TA600 group/1.jpg]

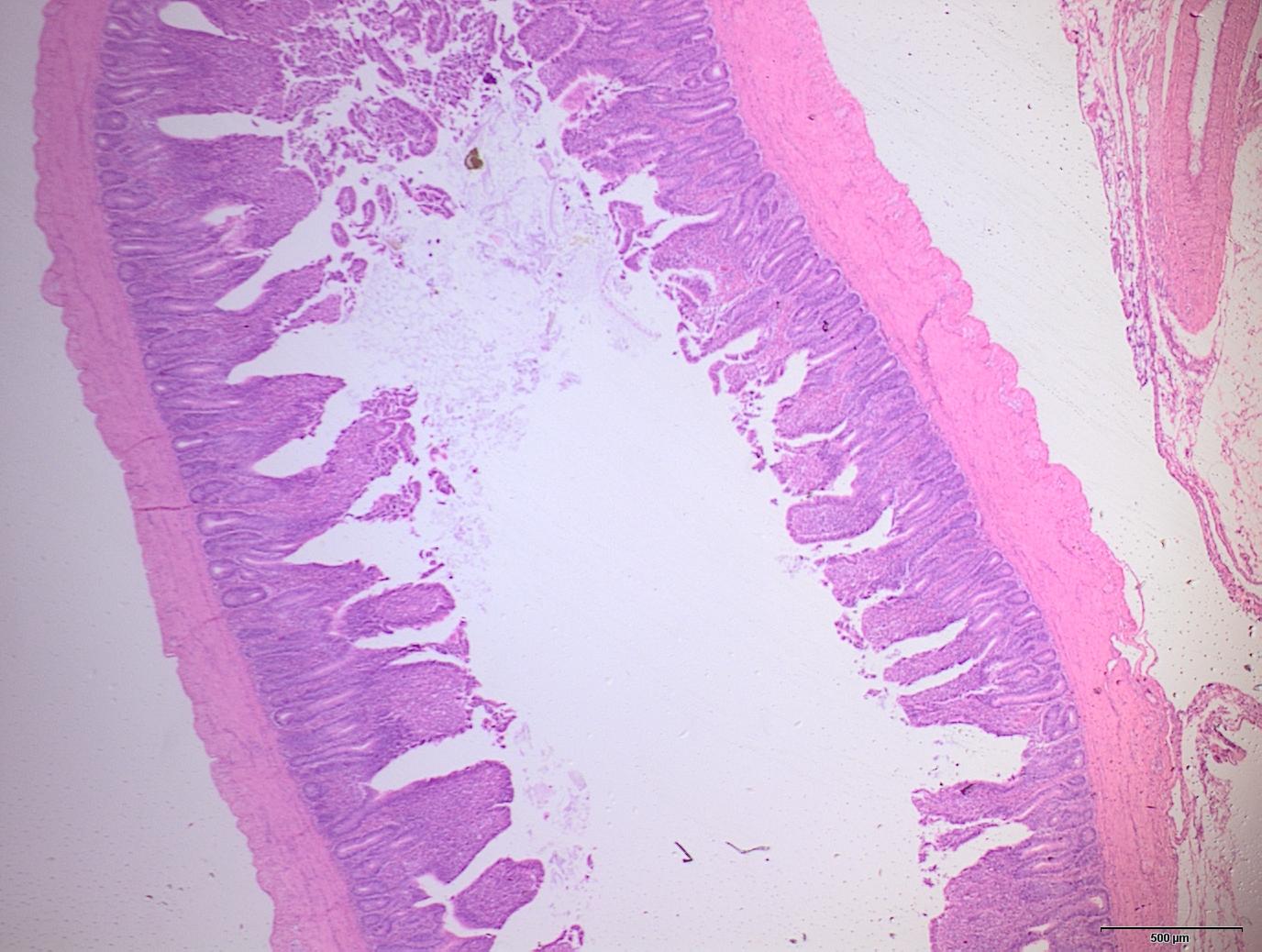

Supplement: Supplementary file 3 [file Data_Sheet_1.ZIP › Data sheet/Hematoxylin-eosin Staining/Ileum/NE+TA600 group/2.jpg]

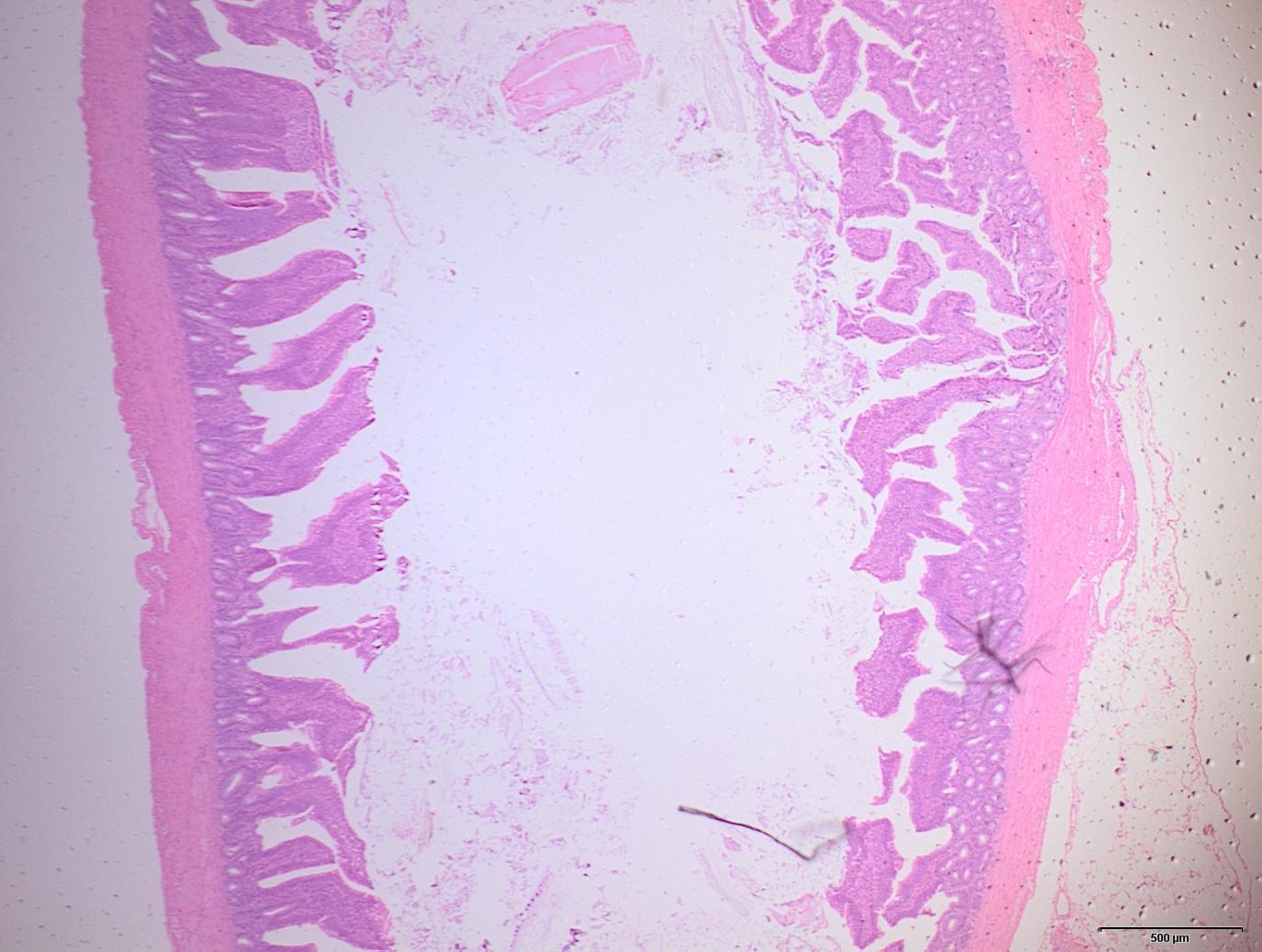

Supplement: Supplementary file 3 [file Data_Sheet_1.ZIP › Data sheet/Hematoxylin-eosin Staining/Ileum/NE+TA600 group/3.jpg]

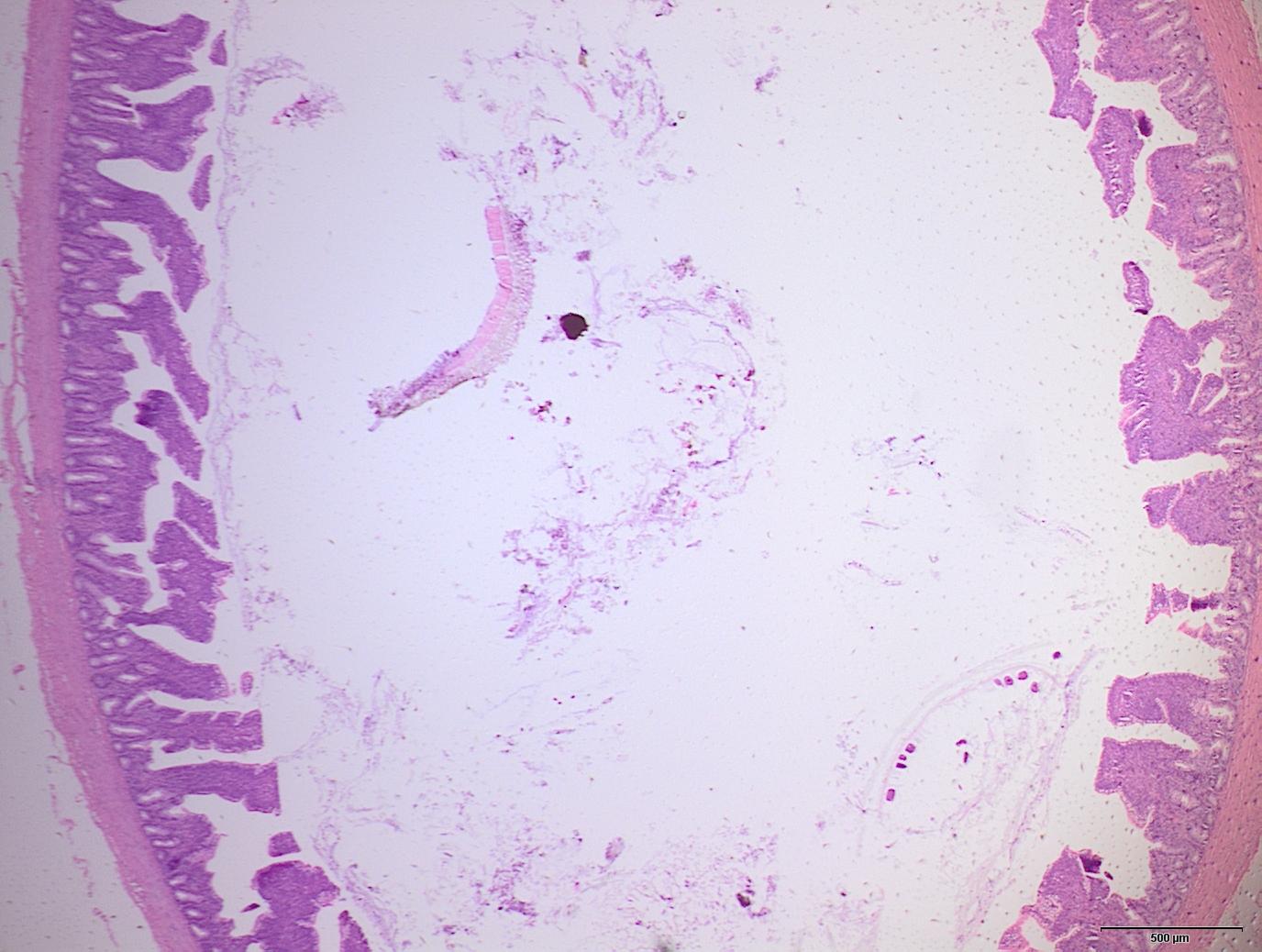

Supplement: Supplementary file 3 [file Data_Sheet_1.ZIP › Data sheet/Hematoxylin-eosin Staining/Ileum/NE+TA600 group/4.jpg]

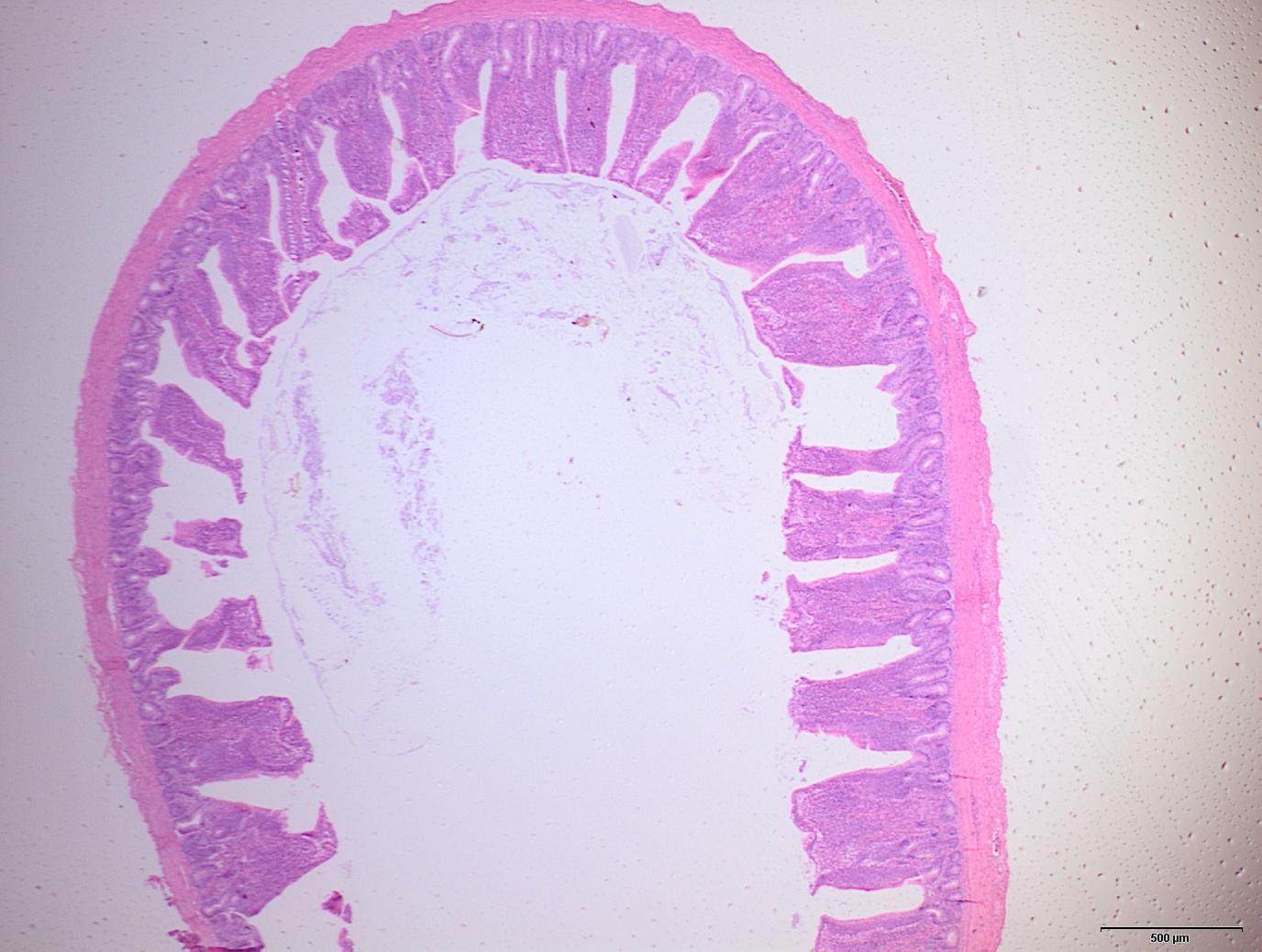

Supplement: Supplementary file 3 [file Data_Sheet_1.ZIP › Data sheet/Hematoxylin-eosin Staining/Ileum/NE+TA600 group/5.jpg]

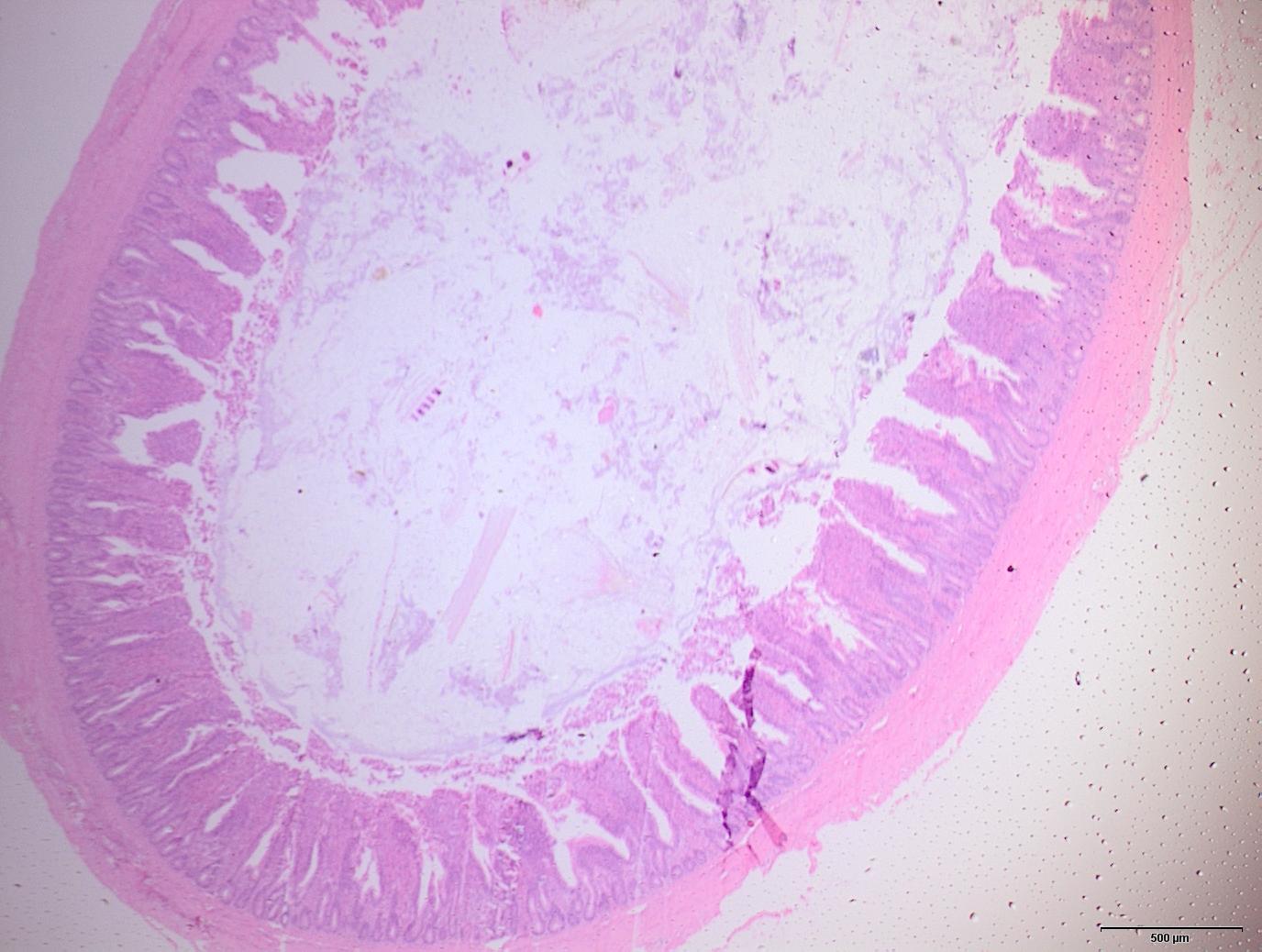

Supplement: Supplementary file 3 [file Data_Sheet_1.ZIP › Data sheet/Hematoxylin-eosin Staining/Ileum/NE+TA600 group/6.jpg]

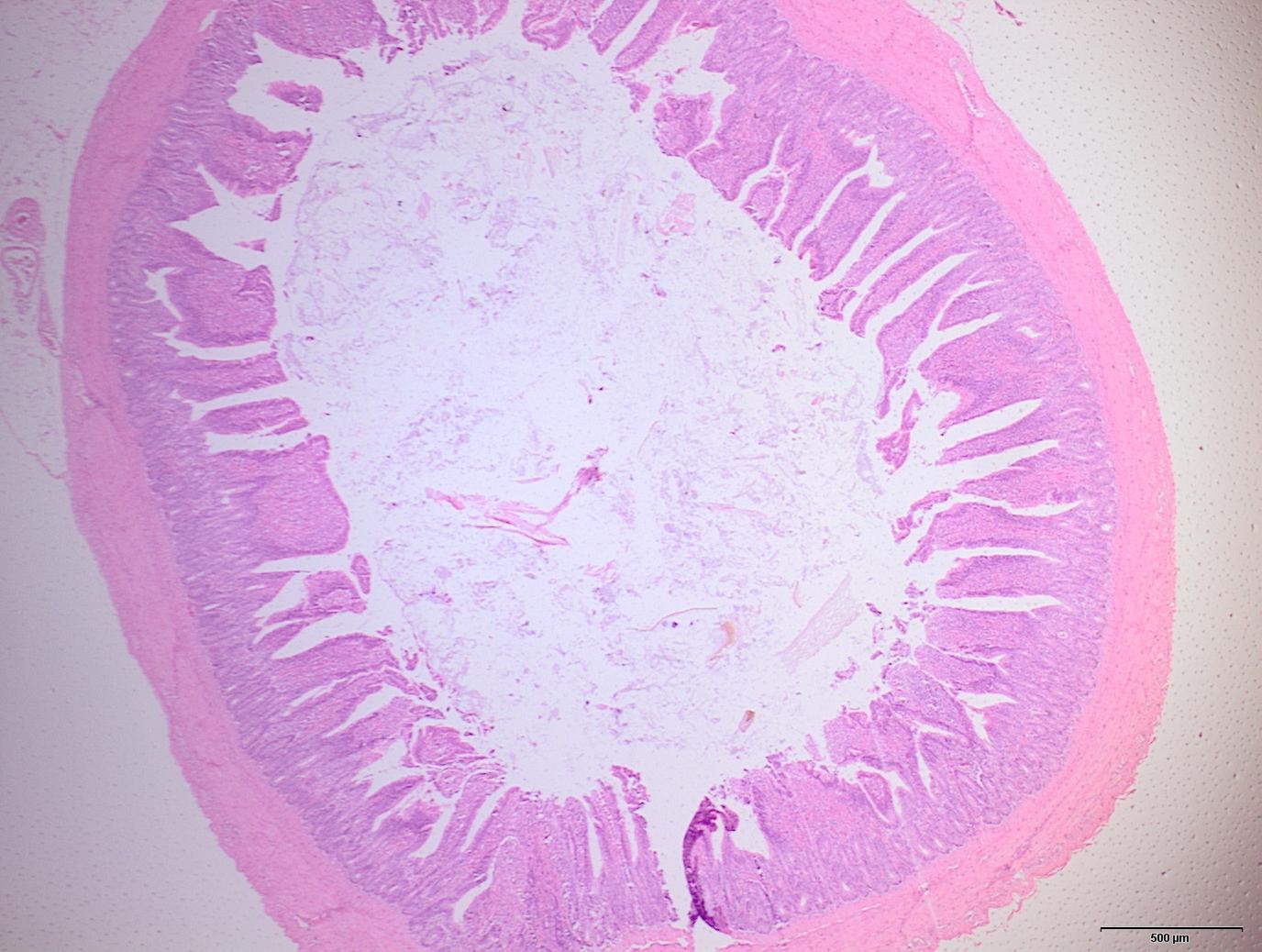

Supplement: Supplementary file 3 [file Data_Sheet_1.ZIP › Data sheet/Hematoxylin-eosin Staining/Ileum/NE+TA600 group/7.jpg]

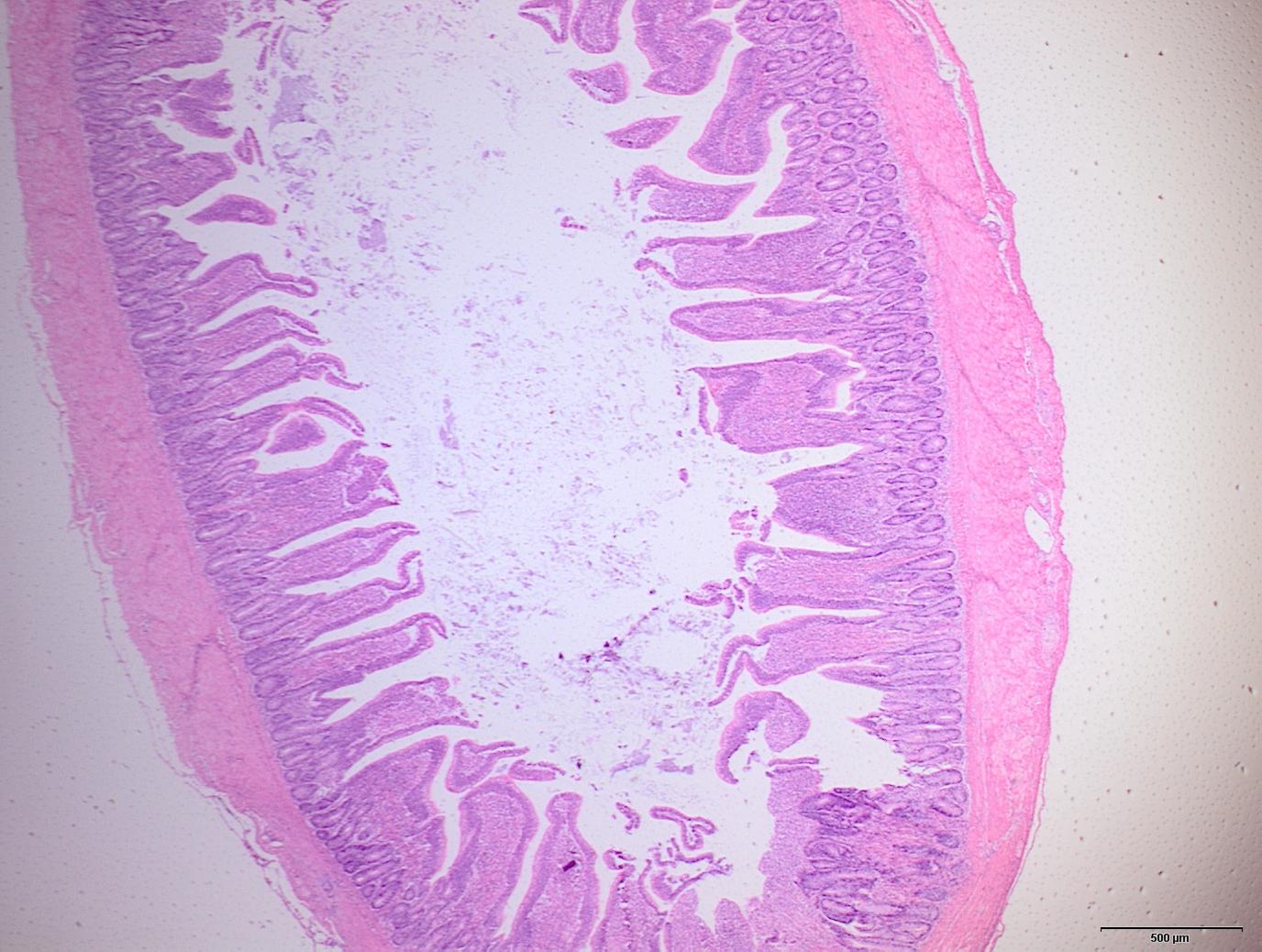

Supplement: Supplementary file 3 [file Data_Sheet_1.ZIP › Data sheet/Hematoxylin-eosin Staining/Ileum/NE+TA600 group/8.jpg]

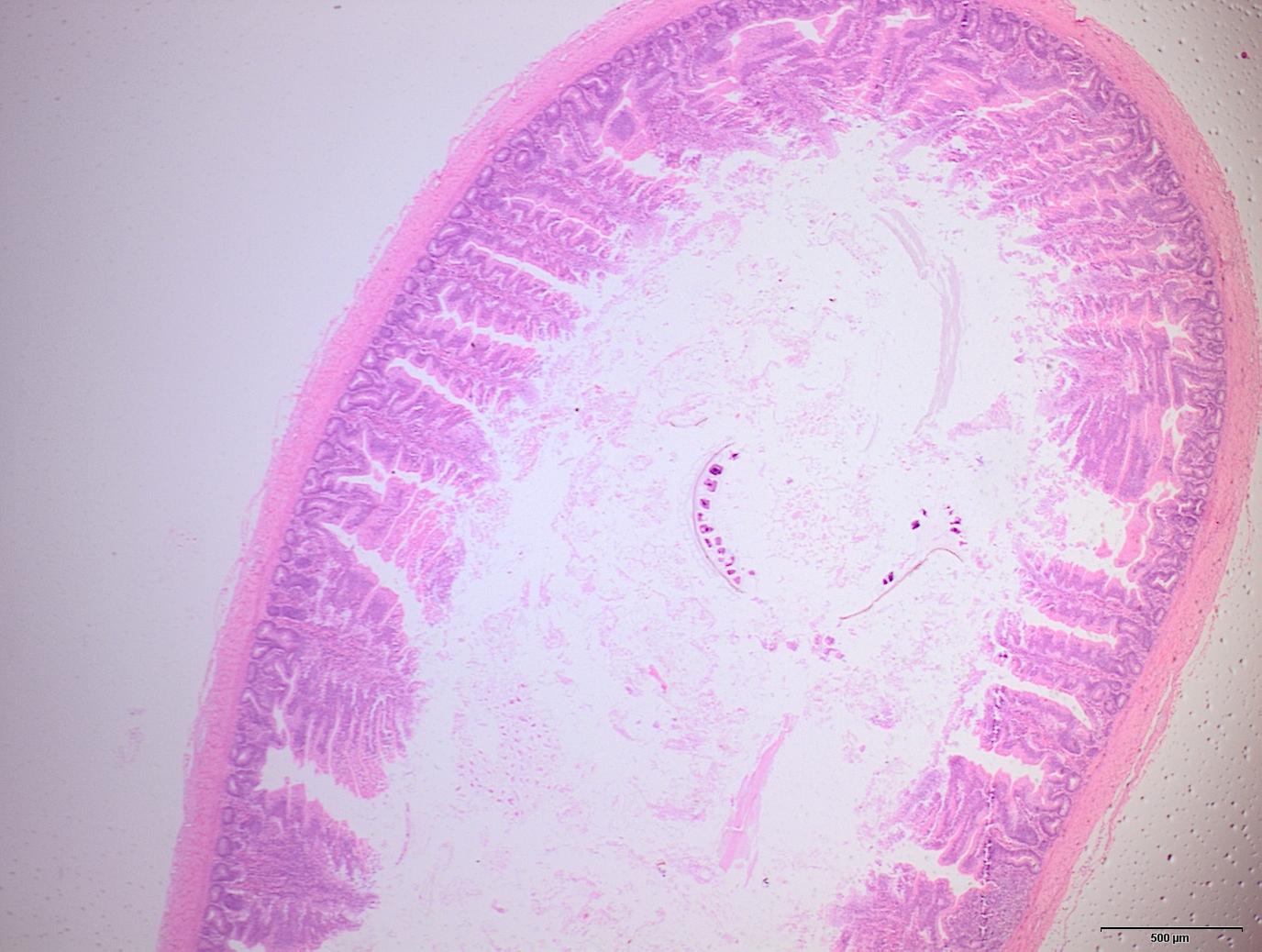

Supplement: Supplementary file 3 [file Data_Sheet_1.ZIP › Data sheet/Hematoxylin-eosin Staining/Jejunum/CON group/1.jpg]

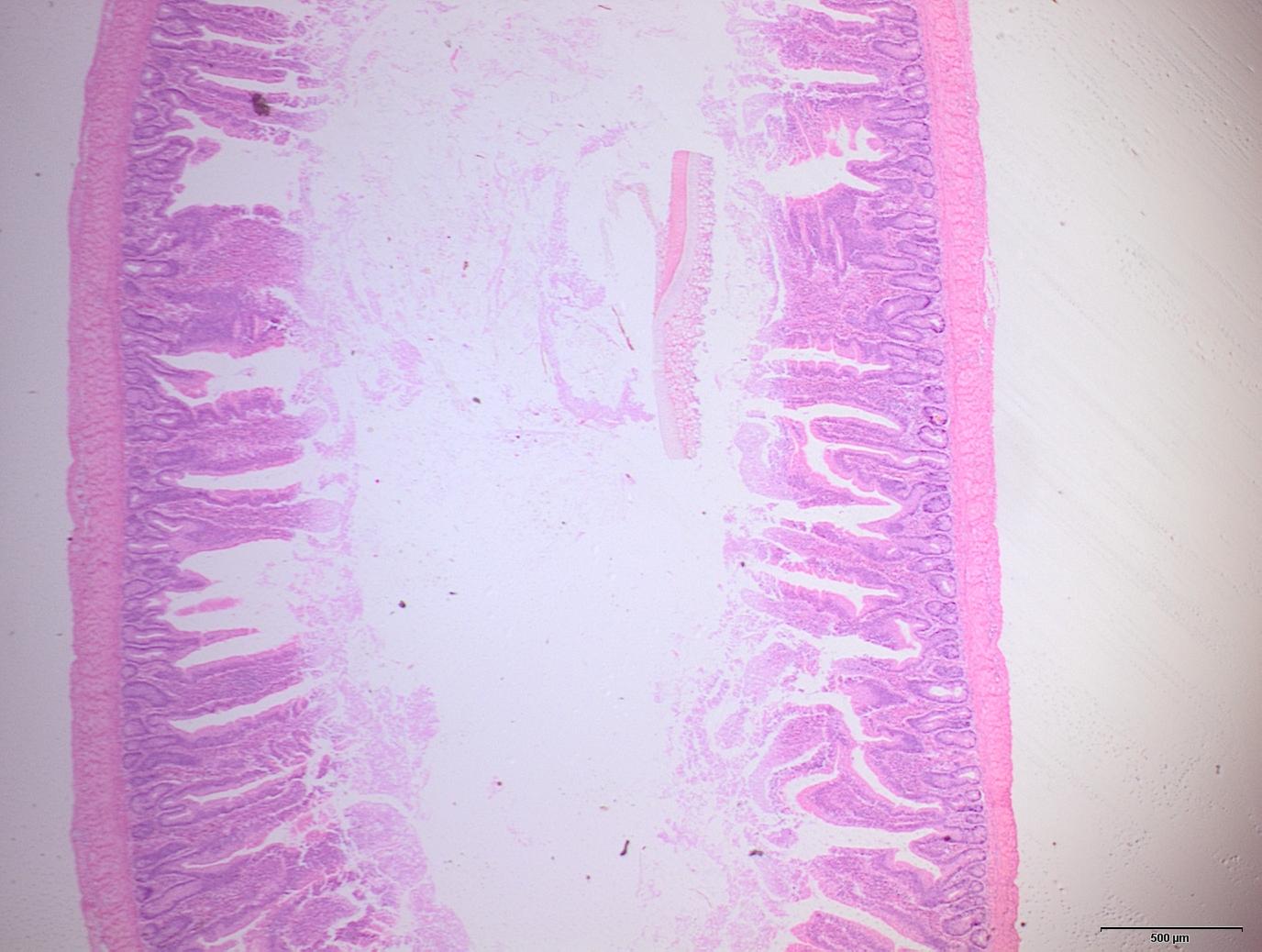

Supplement: Supplementary file 3 [file Data_Sheet_1.ZIP › Data sheet/Hematoxylin-eosin Staining/Jejunum/CON group/2.jpg]

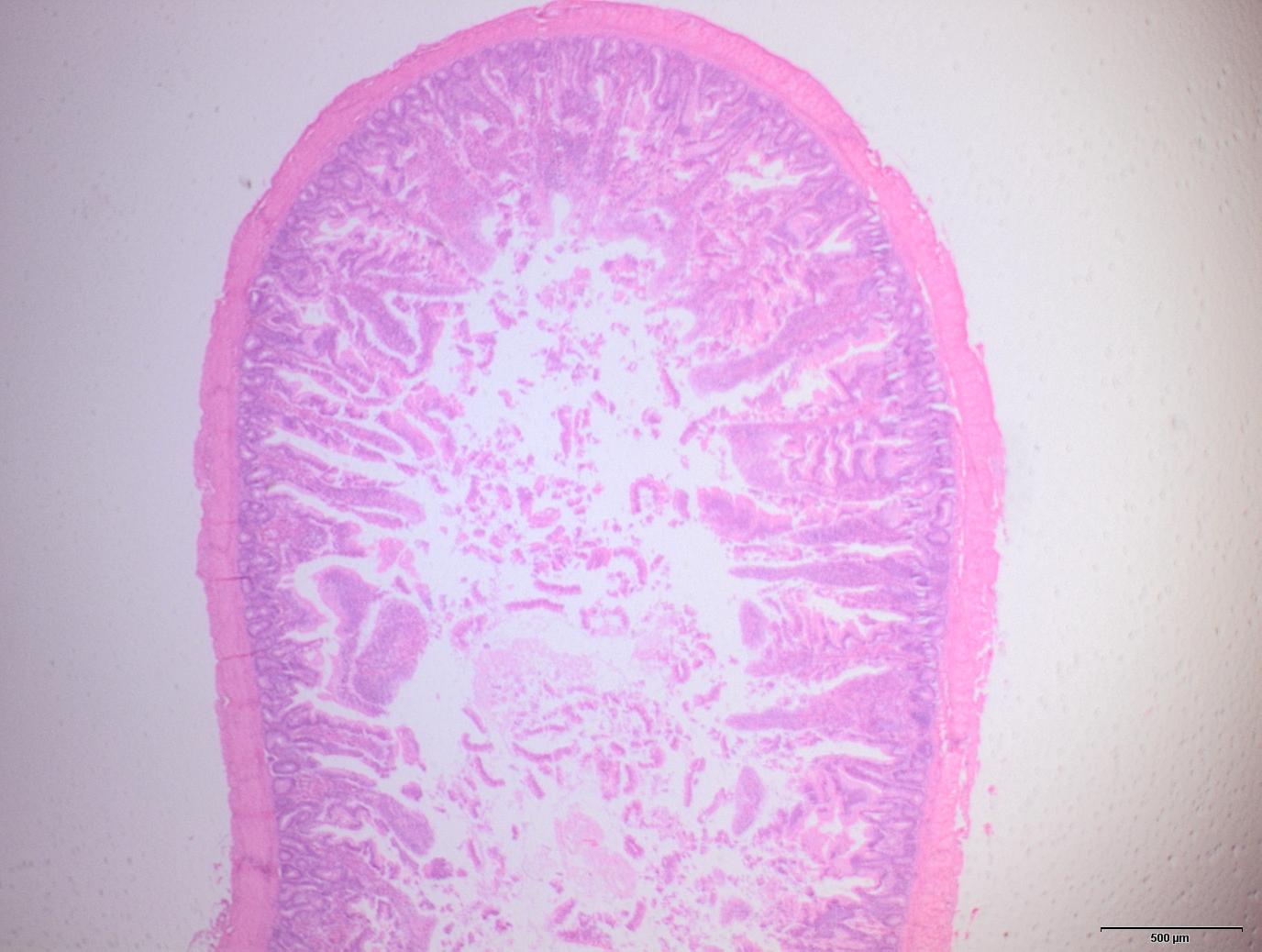

Supplement: Supplementary file 3 [file Data_Sheet_1.ZIP › Data sheet/Hematoxylin-eosin Staining/Jejunum/CON group/3.jpg]

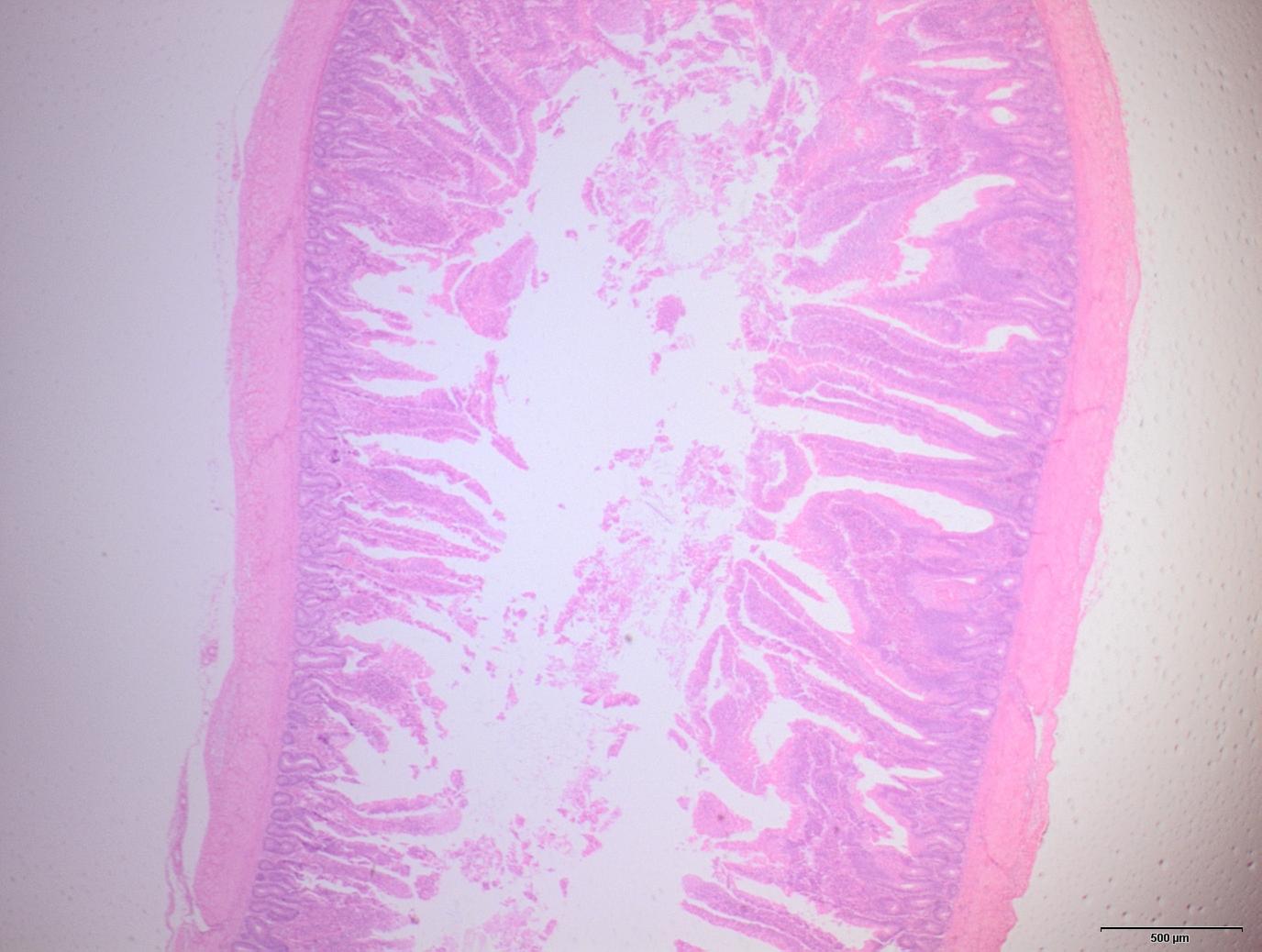

Supplement: Supplementary file 3 [file Data_Sheet_1.ZIP › Data sheet/Hematoxylin-eosin Staining/Jejunum/CON group/4.jpg]

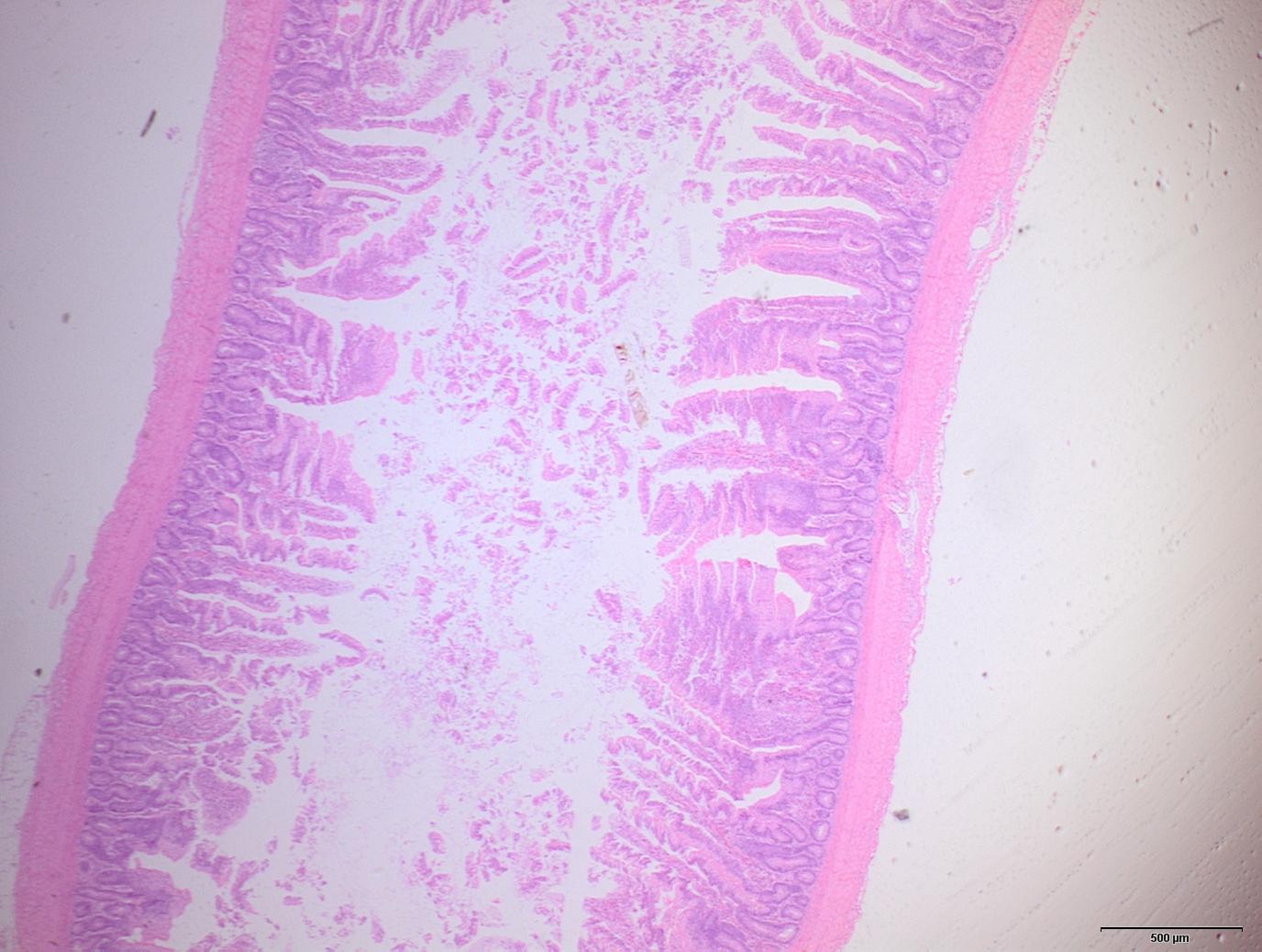

Supplement: Supplementary file 3 [file Data_Sheet_1.ZIP › Data sheet/Hematoxylin-eosin Staining/Jejunum/CON group/5.jpg]

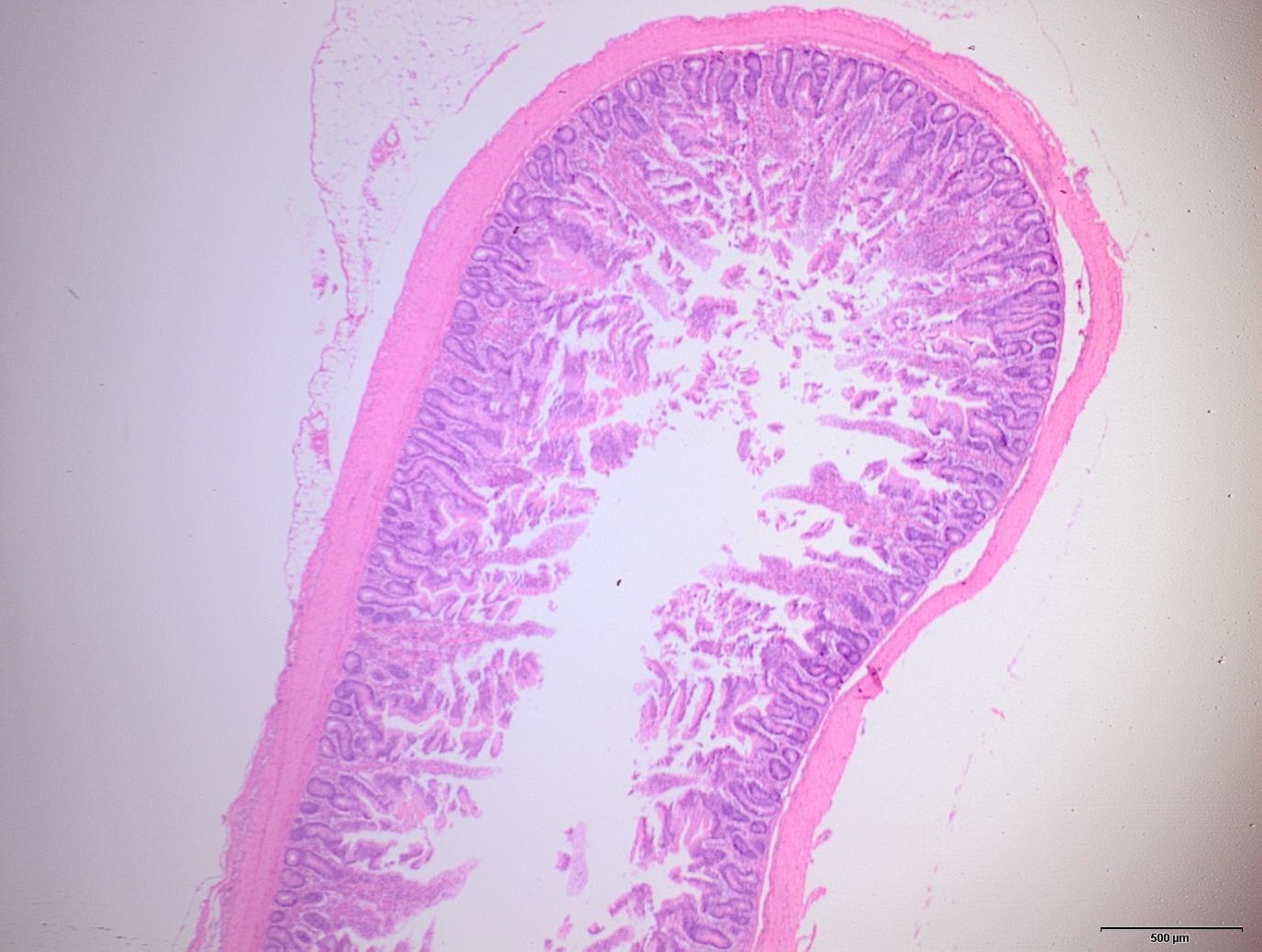

Supplement: Supplementary file 3 [file Data_Sheet_1.ZIP › Data sheet/Hematoxylin-eosin Staining/Jejunum/CON group/6.jpg]

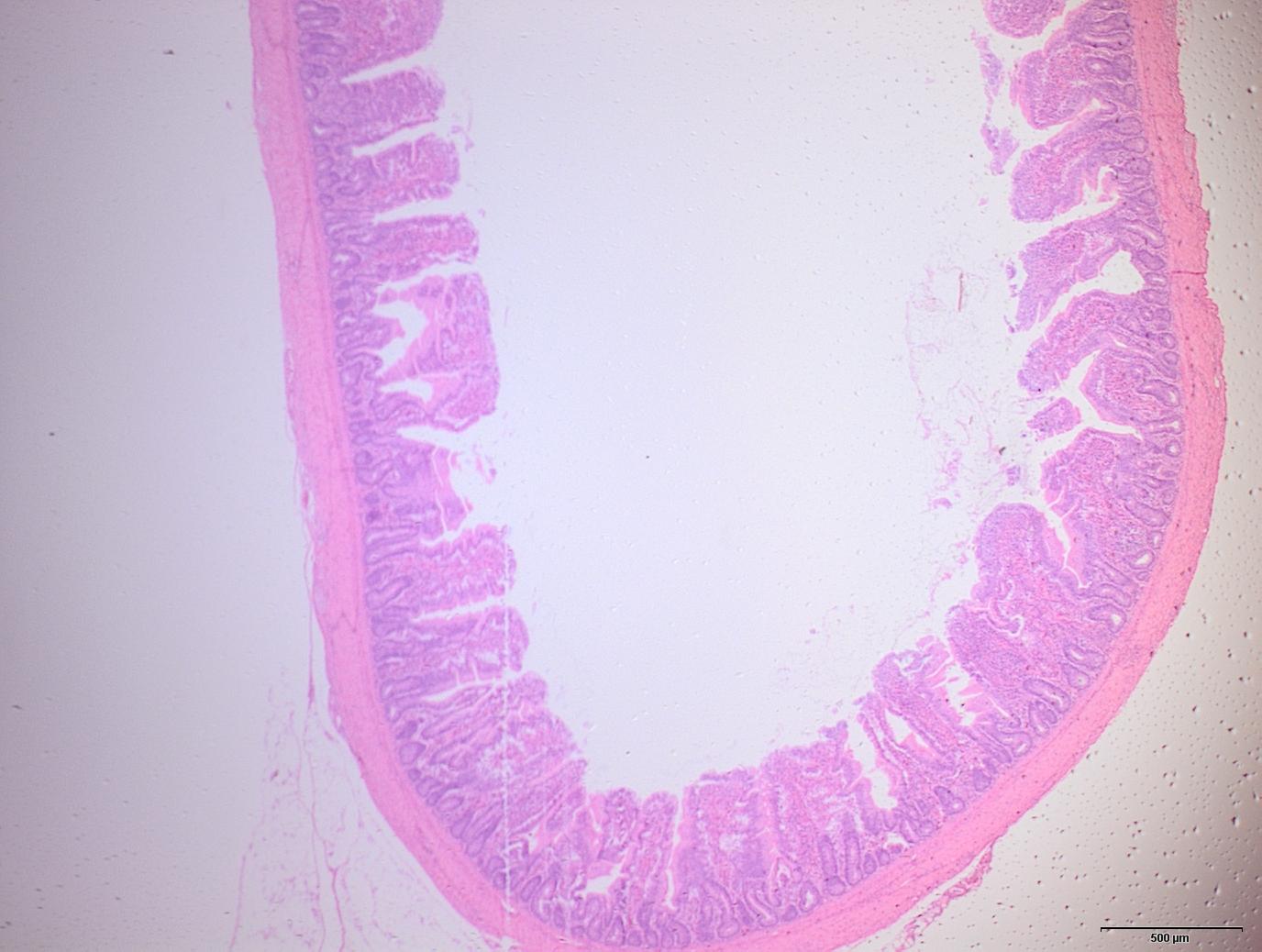

Supplement: Supplementary file 3 [file Data_Sheet_1.ZIP › Data sheet/Hematoxylin-eosin Staining/Jejunum/CON group/7.jpg]

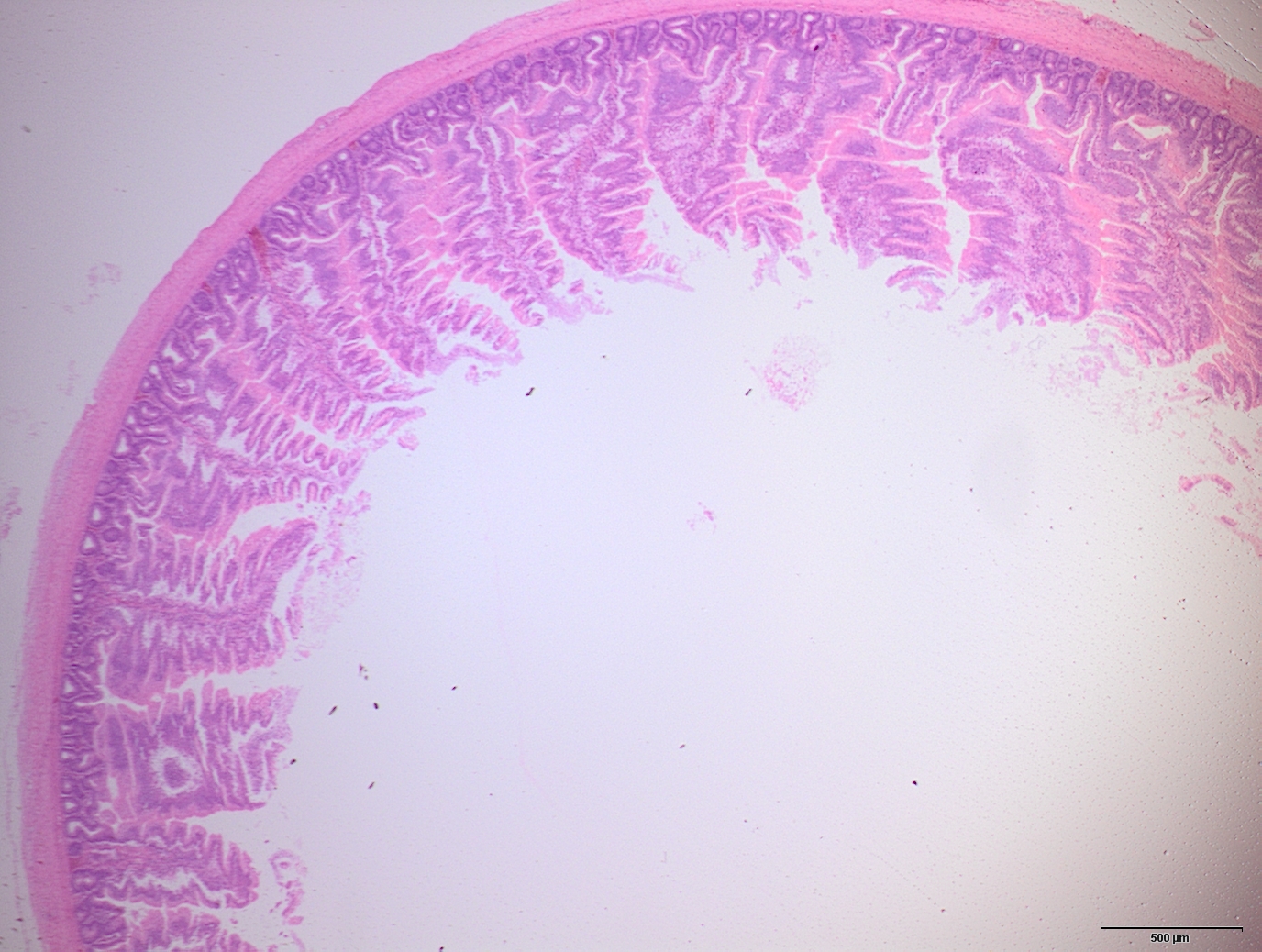

Supplement: Supplementary file 3 [file Data_Sheet_1.ZIP › Data sheet/Hematoxylin-eosin Staining/Jejunum/CON group/8.jpg]

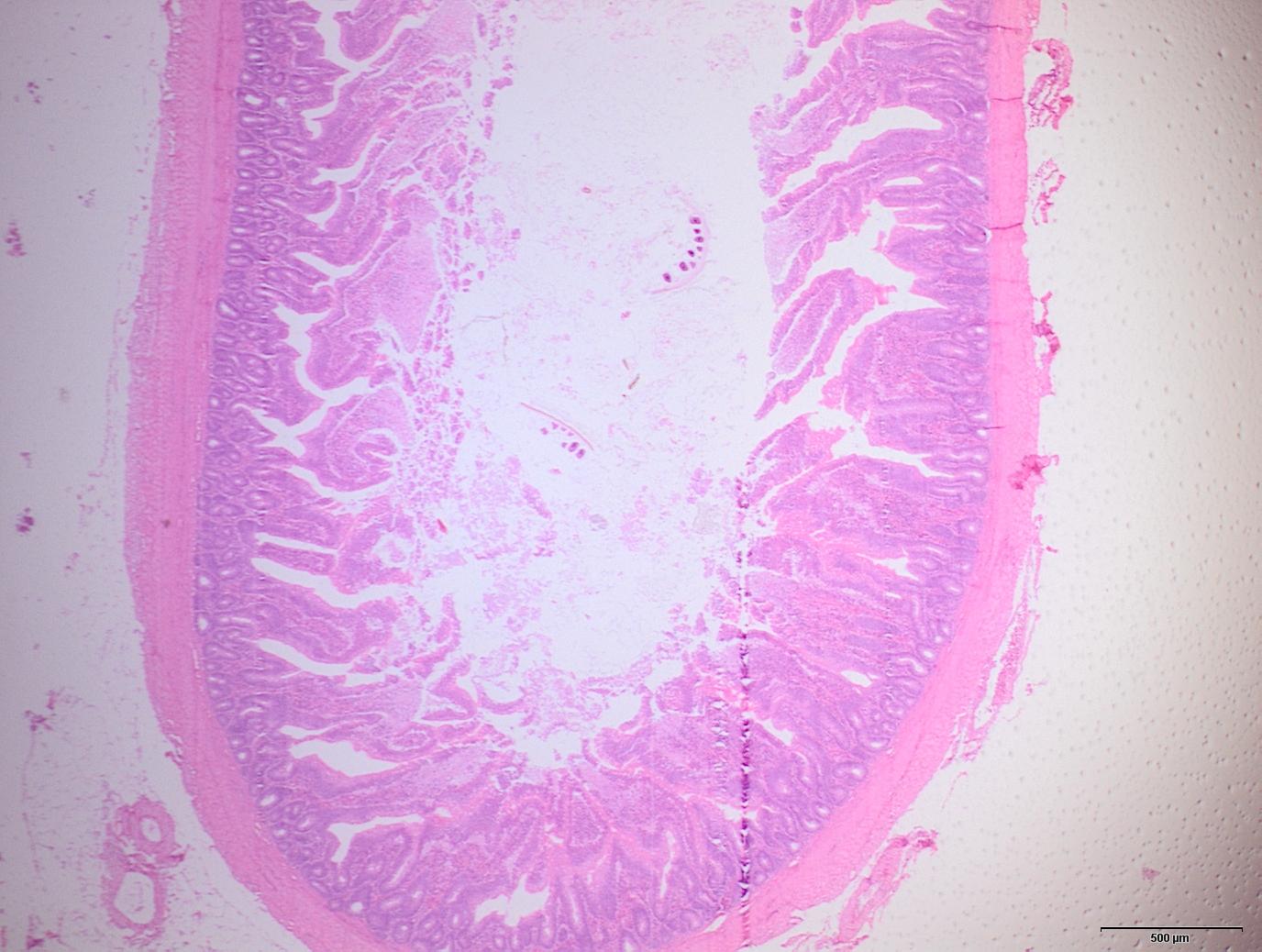

Supplement: Supplementary file 3 [file Data_Sheet_1.ZIP › Data sheet/Hematoxylin-eosin Staining/Jejunum/NE group/1.jpg]

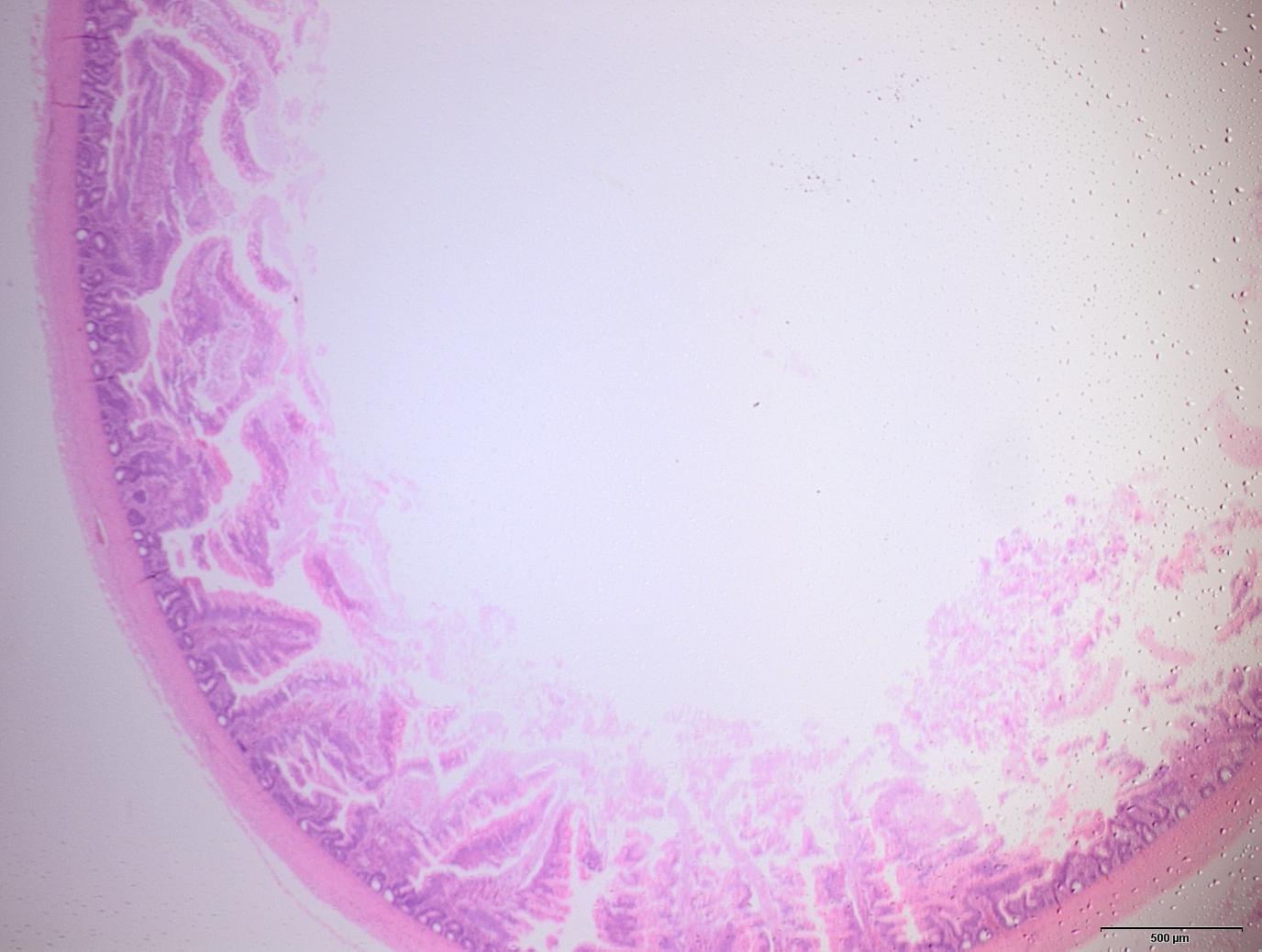

Supplement: Supplementary file 3 [file Data_Sheet_1.ZIP › Data sheet/Hematoxylin-eosin Staining/Jejunum/NE group/2.jpg]

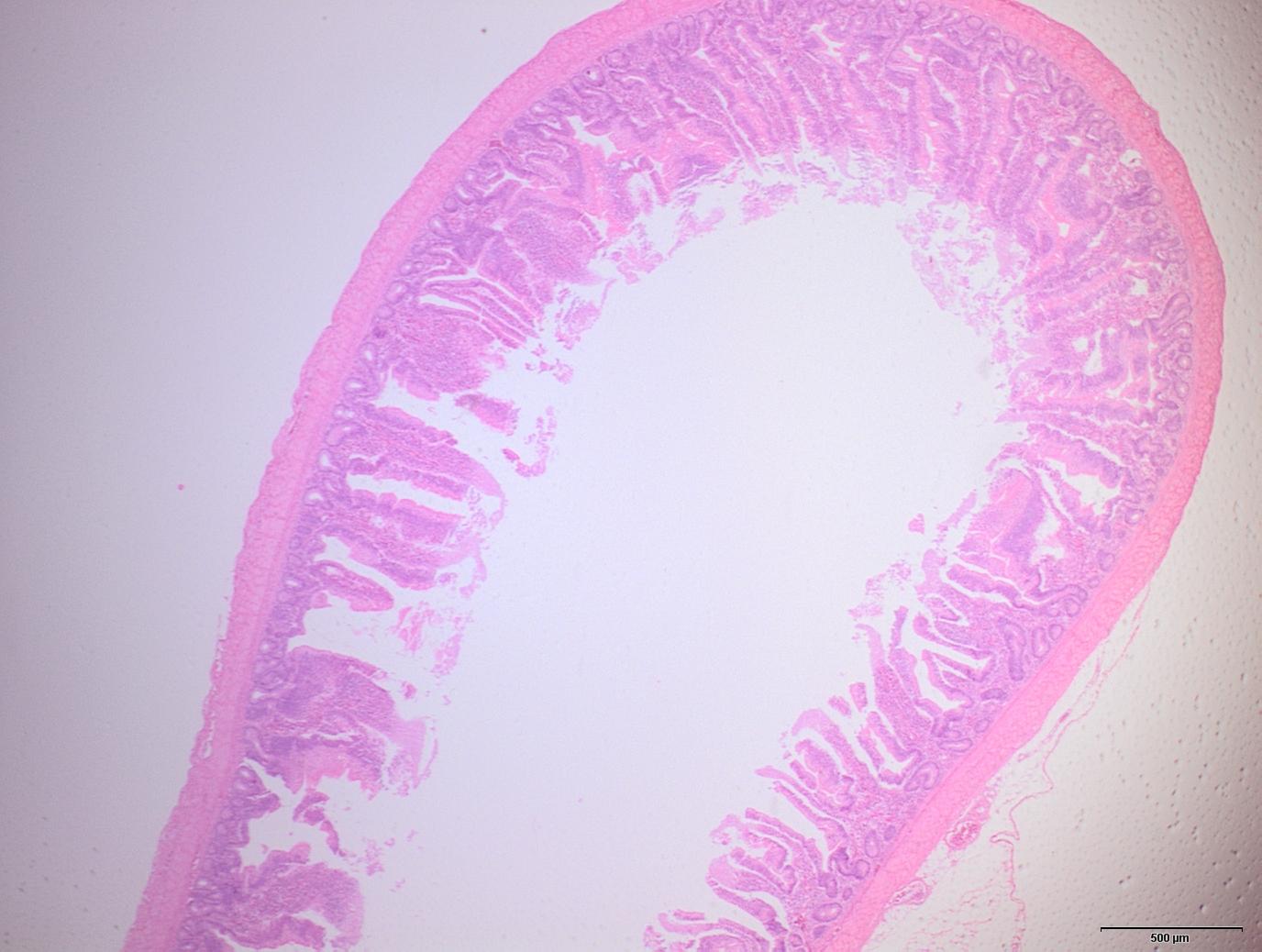

Supplement: Supplementary file 3 [file Data_Sheet_1.ZIP › Data sheet/Hematoxylin-eosin Staining/Jejunum/NE group/3.jpg]

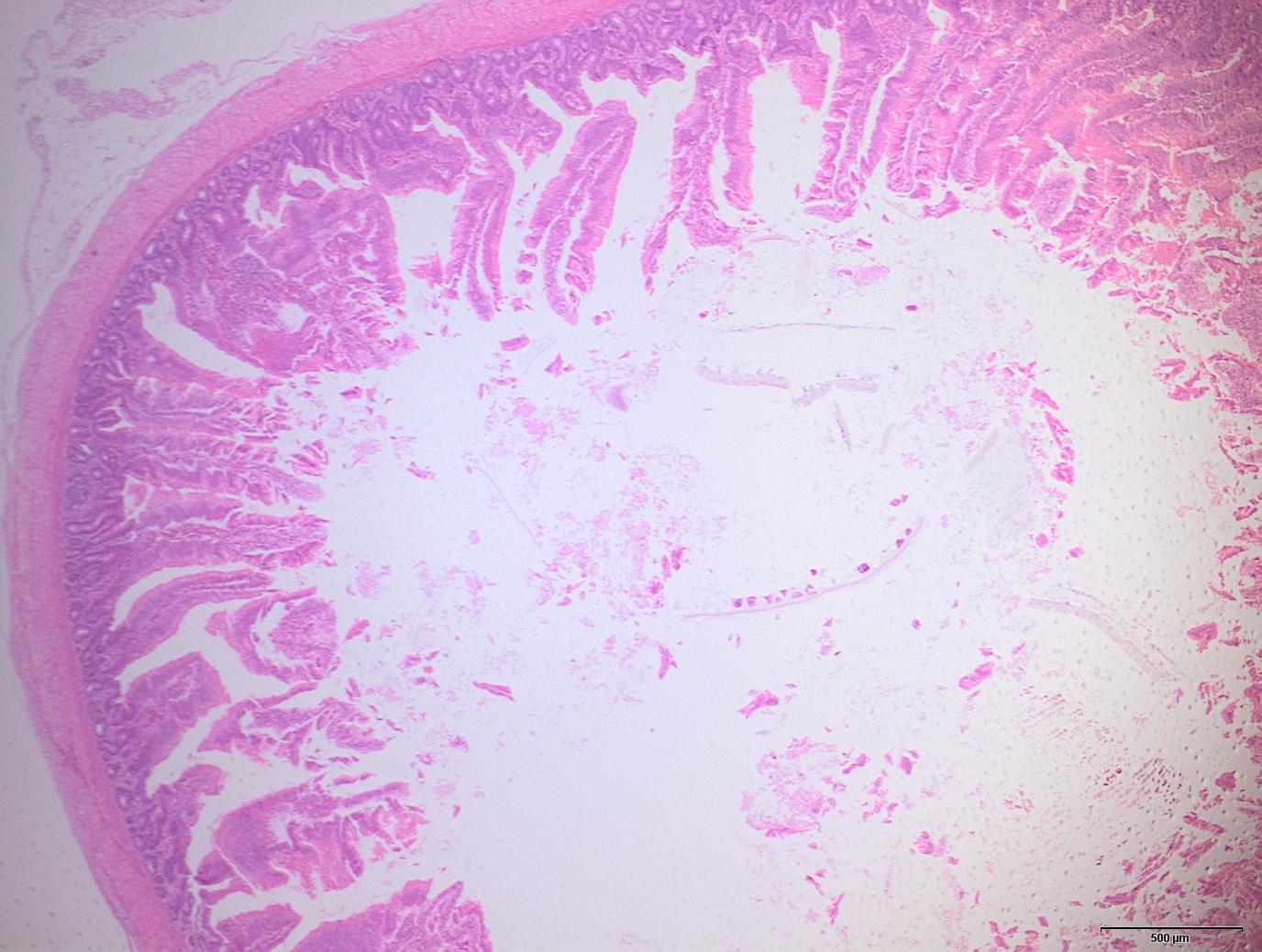

Supplement: Supplementary file 3 [file Data_Sheet_1.ZIP › Data sheet/Hematoxylin-eosin Staining/Jejunum/NE group/4.jpg]

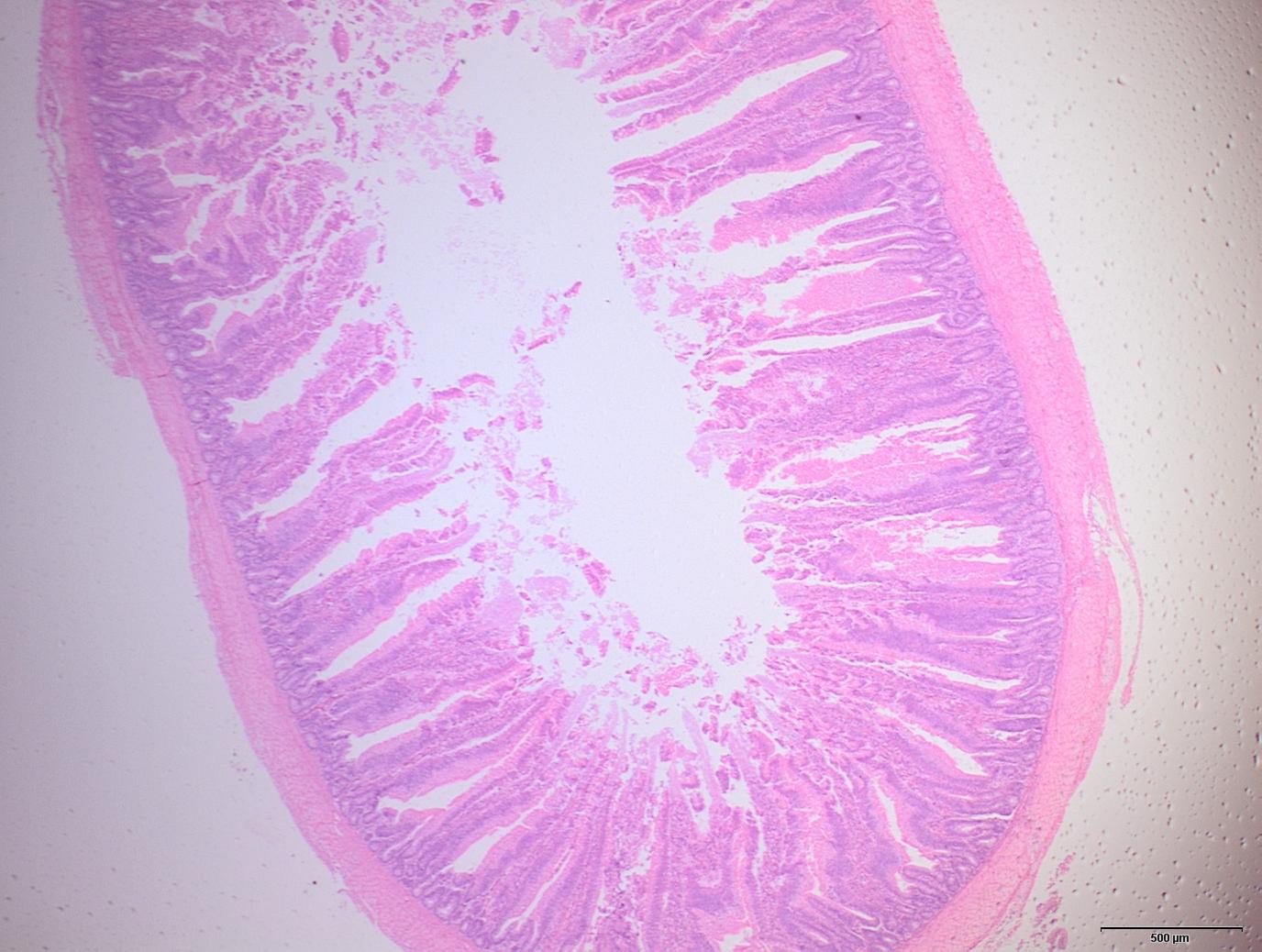

Supplement: Supplementary file 3 [file Data_Sheet_1.ZIP › Data sheet/Hematoxylin-eosin Staining/Jejunum/NE group/5.jpg]

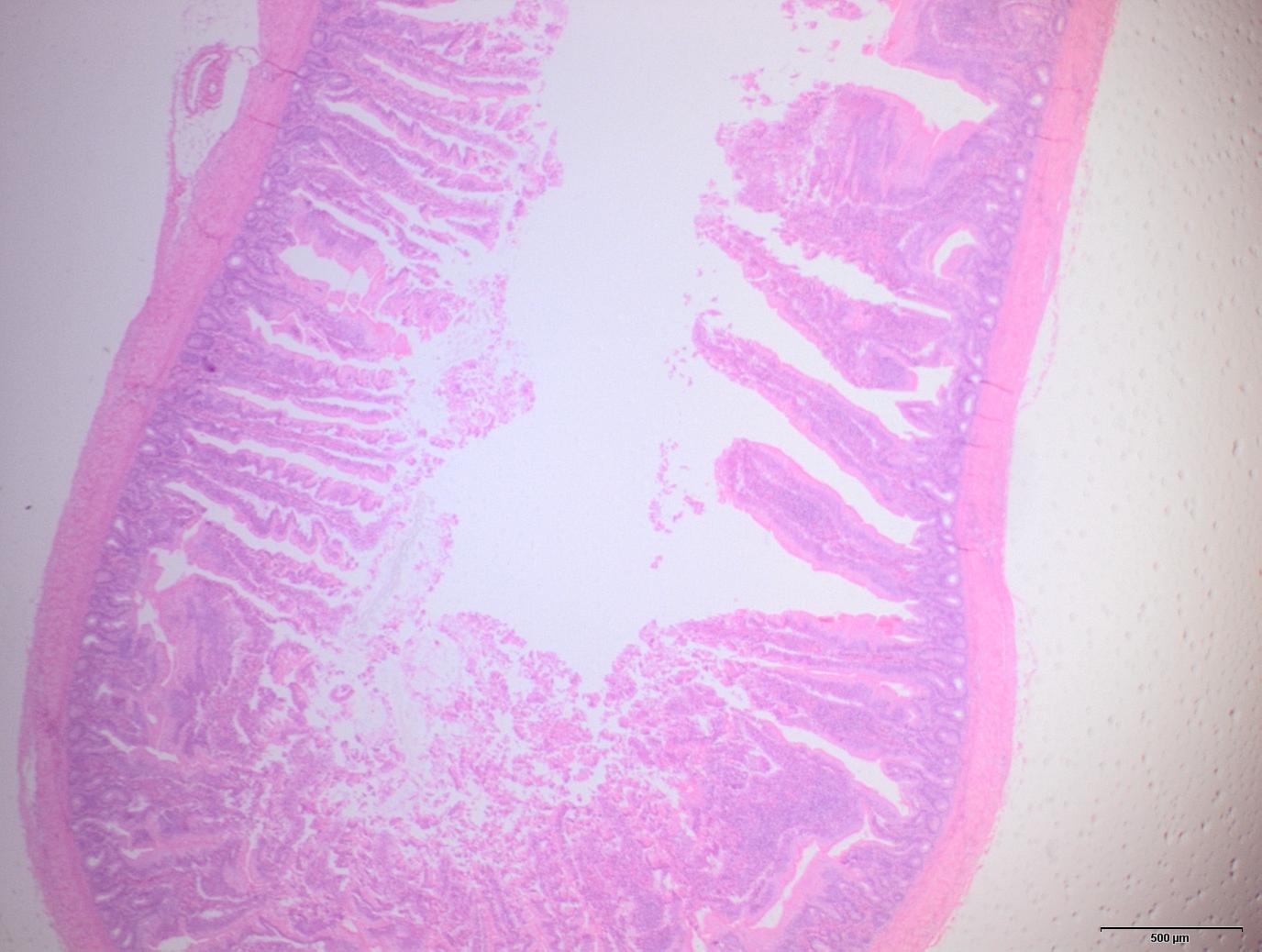

Supplement: Supplementary file 3 [file Data_Sheet_1.ZIP › Data sheet/Hematoxylin-eosin Staining/Jejunum/NE group/6.jpg]

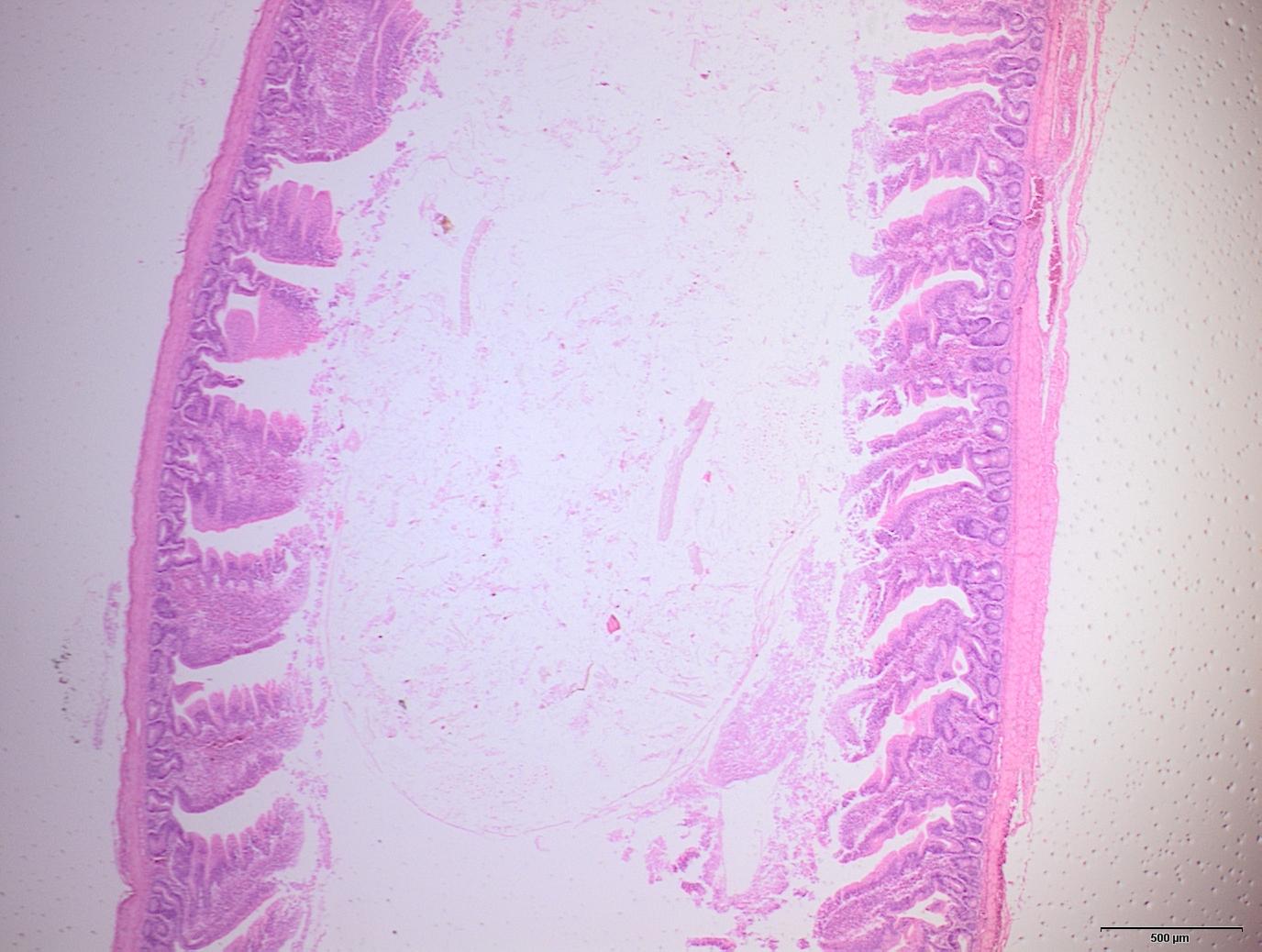

Supplement: Supplementary file 3 [file Data_Sheet_1.ZIP › Data sheet/Hematoxylin-eosin Staining/Jejunum/NE group/7.jpg]

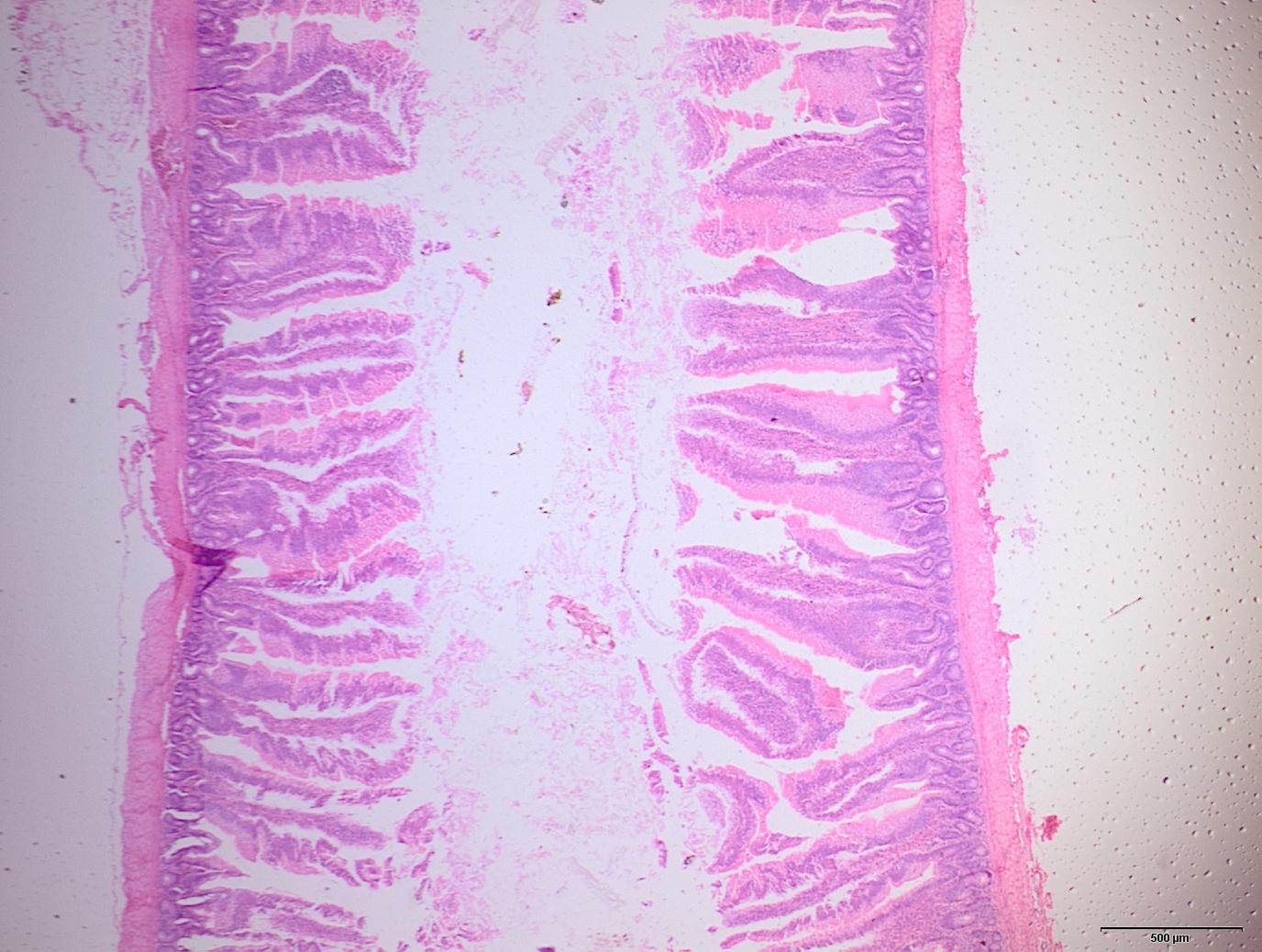

Supplement: Supplementary file 3 [file Data_Sheet_1.ZIP › Data sheet/Hematoxylin-eosin Staining/Jejunum/NE group/8.jpg]

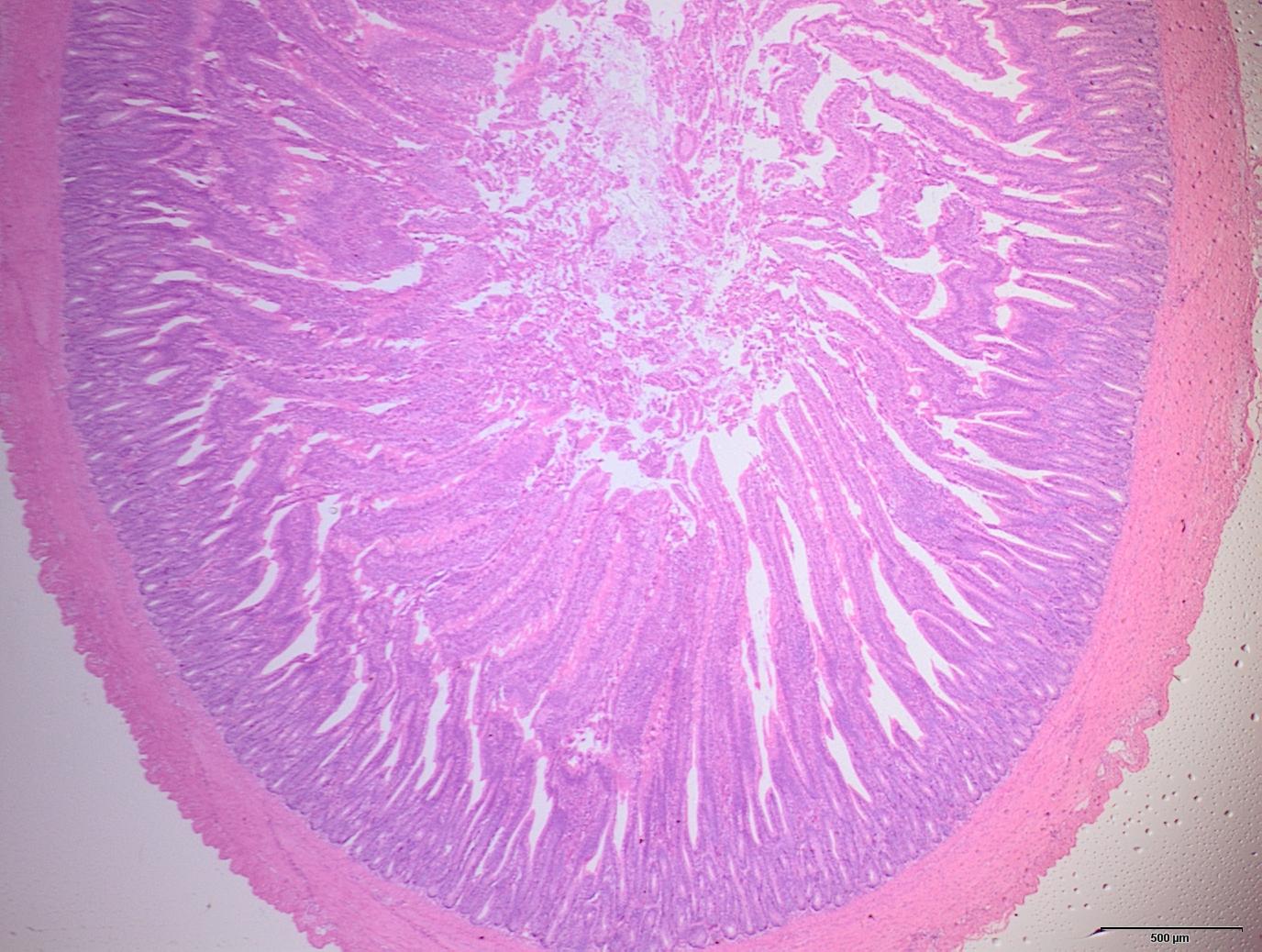

Supplement: Supplementary file 3 [file Data_Sheet_1.ZIP › Data sheet/Hematoxylin-eosin Staining/Jejunum/NE+TA600 group/1.jpg]

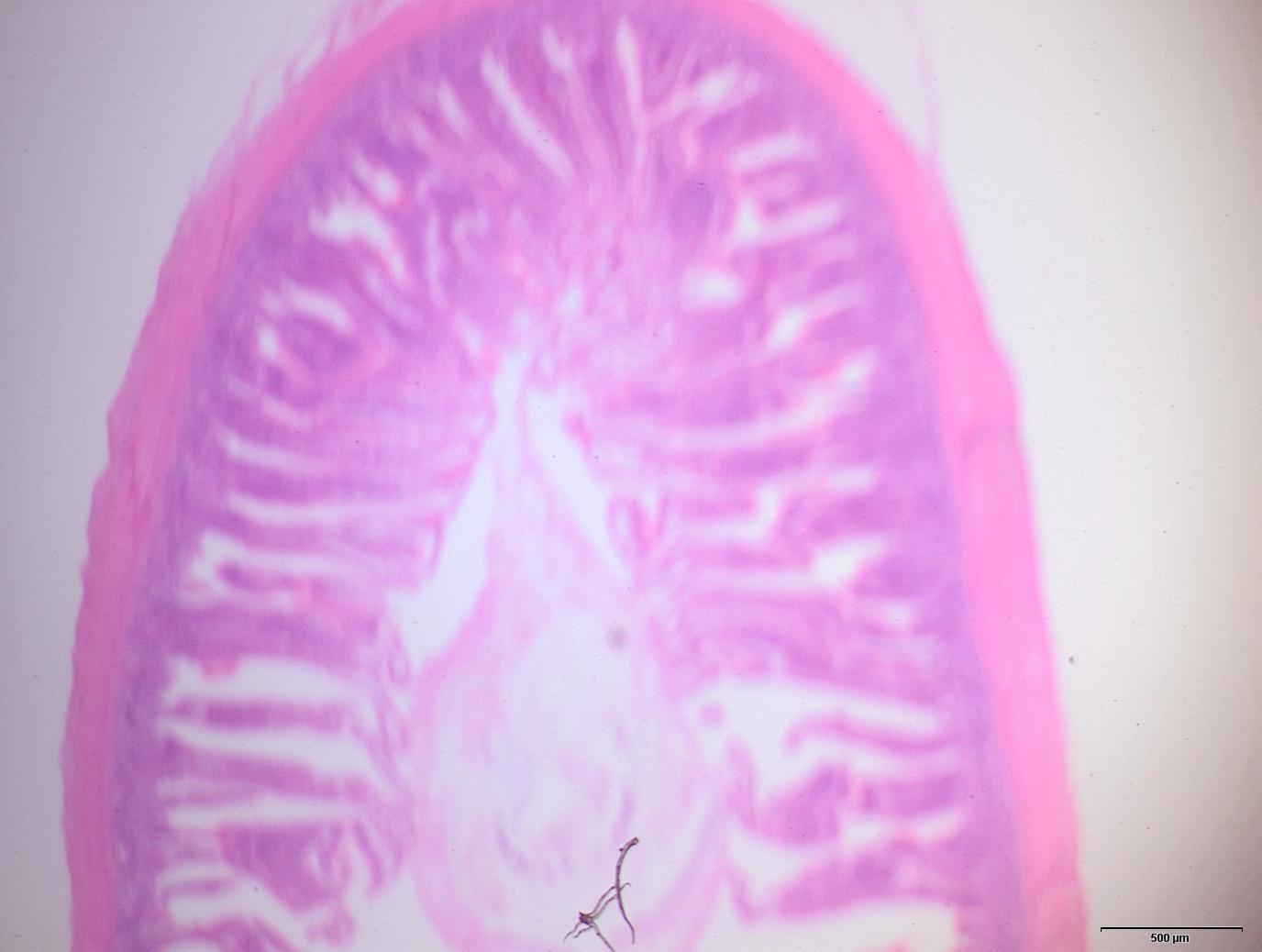

Supplement: Supplementary file 3 [file Data_Sheet_1.ZIP › Data sheet/Hematoxylin-eosin Staining/Jejunum/NE+TA600 group/2.jpg]

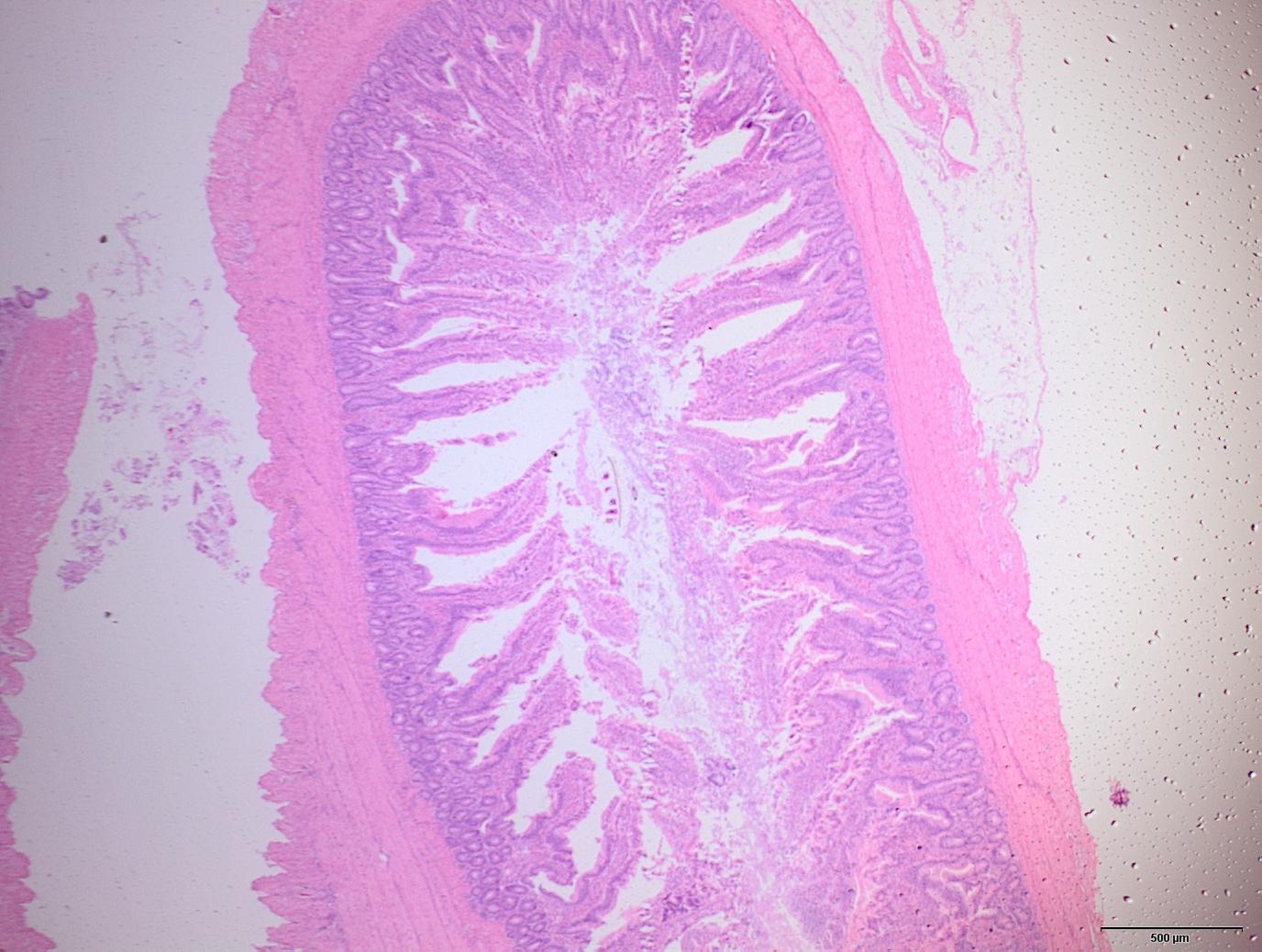

Supplement: Supplementary file 3 [file Data_Sheet_1.ZIP › Data sheet/Hematoxylin-eosin Staining/Jejunum/NE+TA600 group/3.jpg]

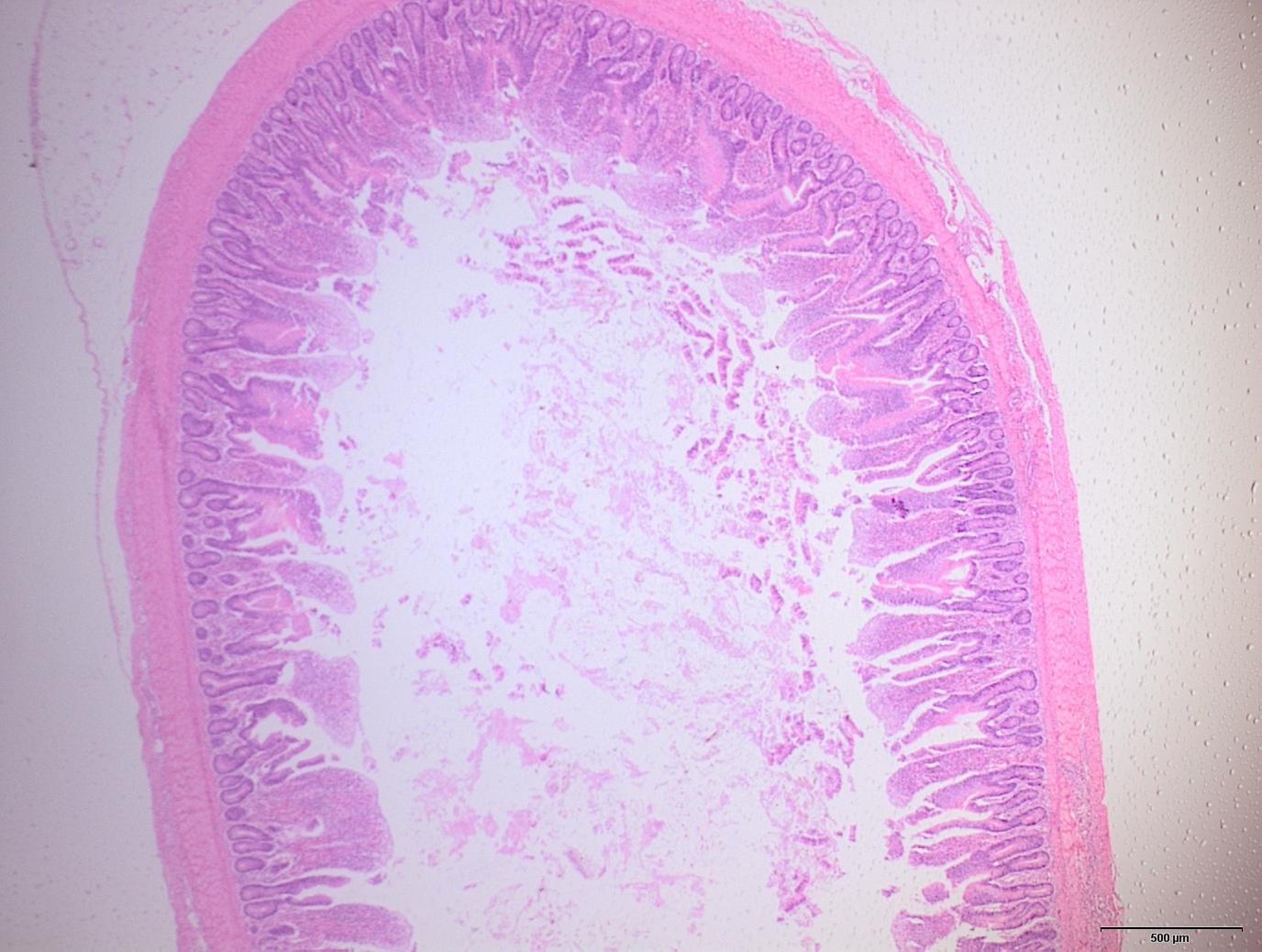

Supplement: Supplementary file 3 [file Data_Sheet_1.ZIP › Data sheet/Hematoxylin-eosin Staining/Jejunum/NE+TA600 group/4.jpg]

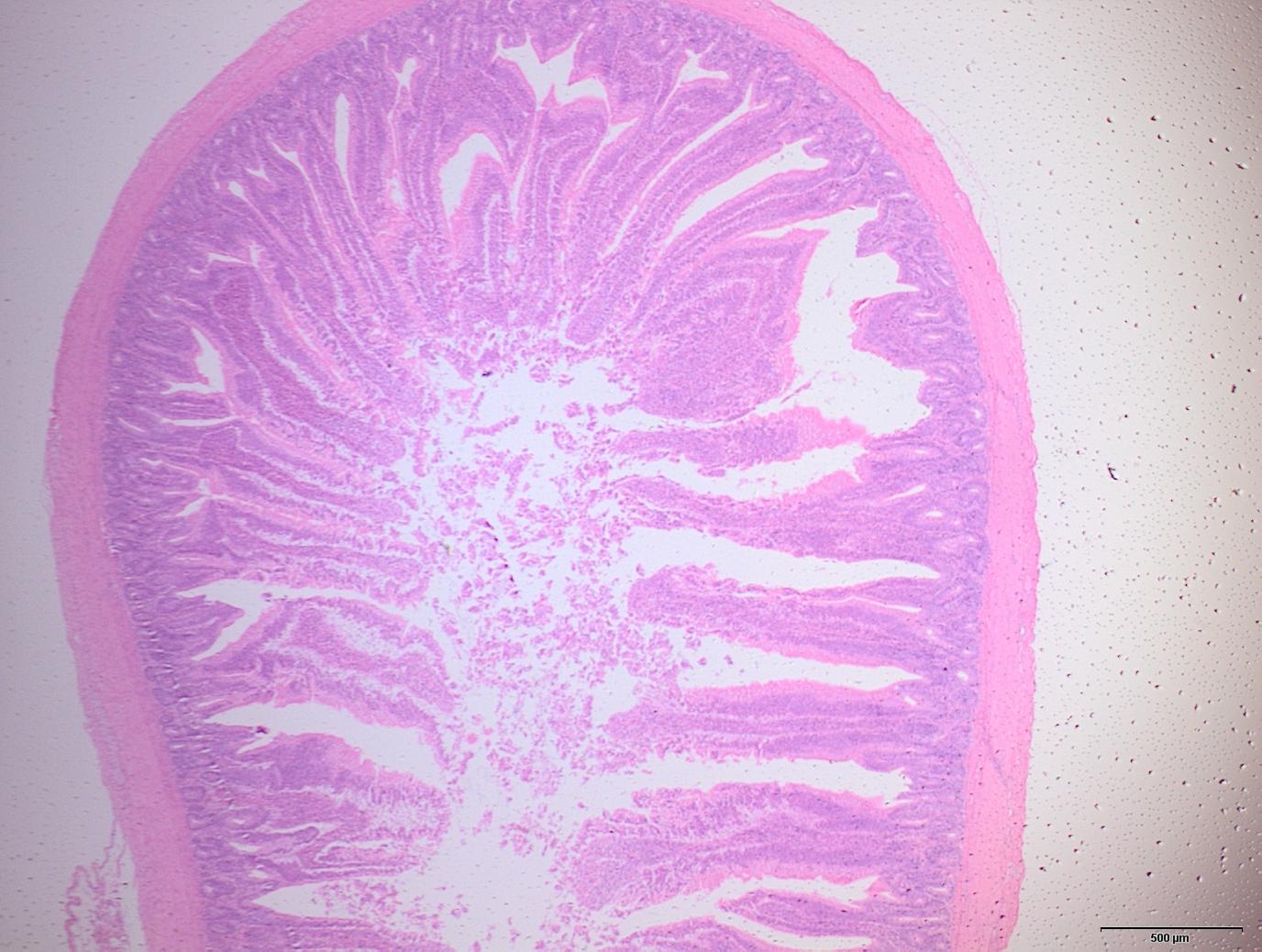

Supplement: Supplementary file 3 [file Data_Sheet_1.ZIP › Data sheet/Hematoxylin-eosin Staining/Jejunum/NE+TA600 group/5.jpg]

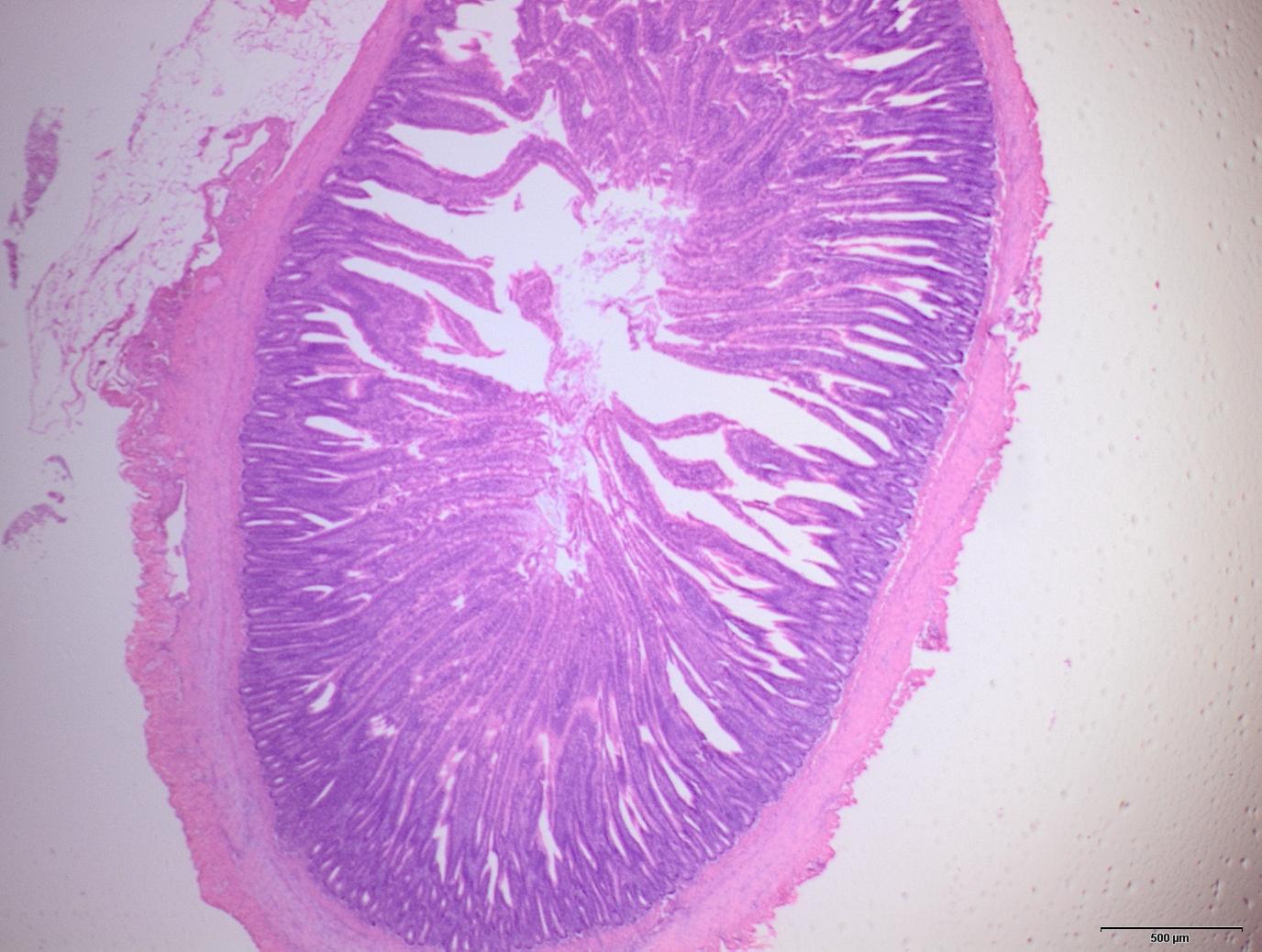

Supplement: Supplementary file 3 [file Data_Sheet_1.ZIP › Data sheet/Hematoxylin-eosin Staining/Jejunum/NE+TA600 group/6.jpg]

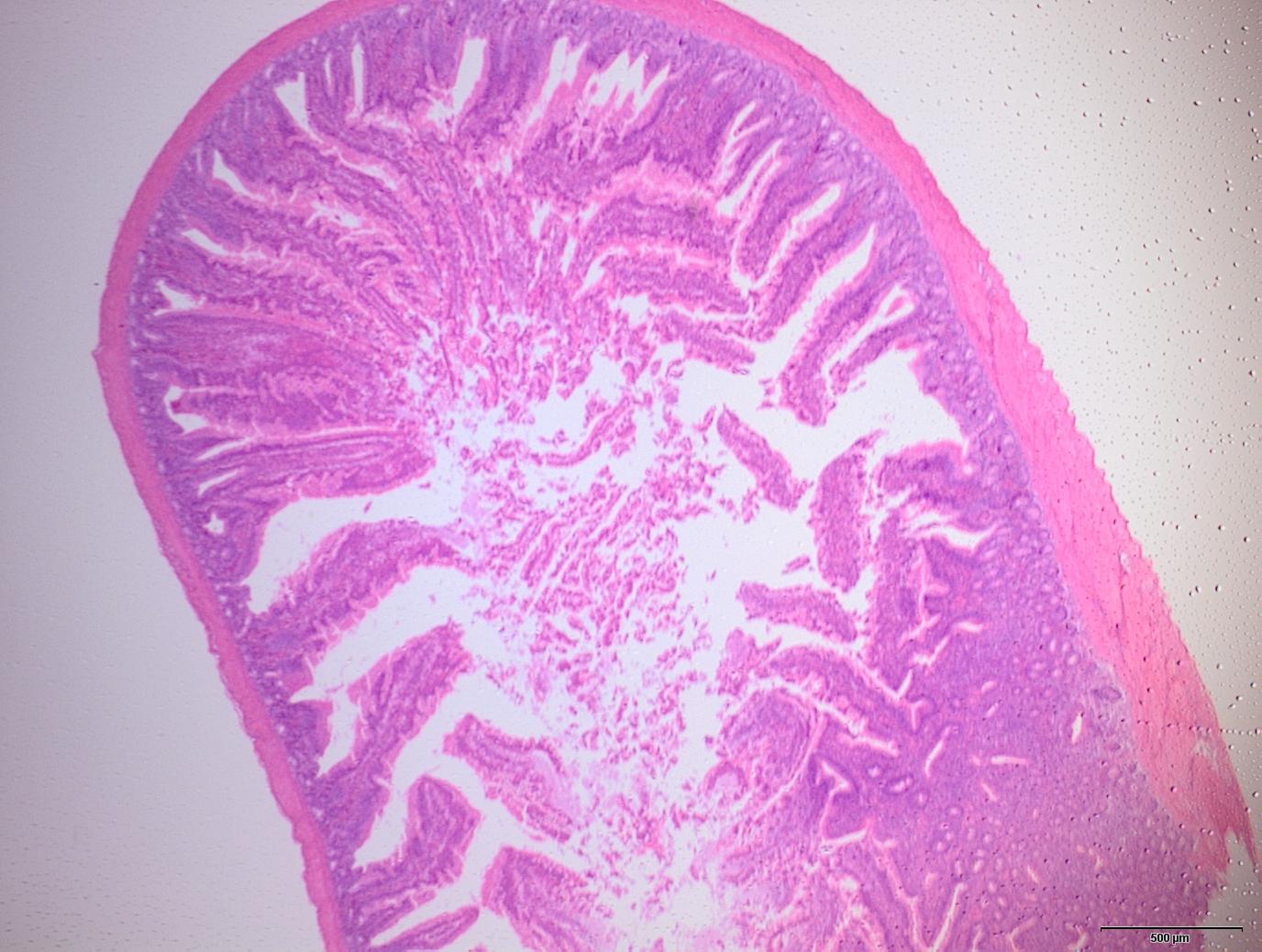

Supplement: Supplementary file 3 [file Data_Sheet_1.ZIP › Data sheet/Hematoxylin-eosin Staining/Jejunum/NE+TA600 group/7.jpg]

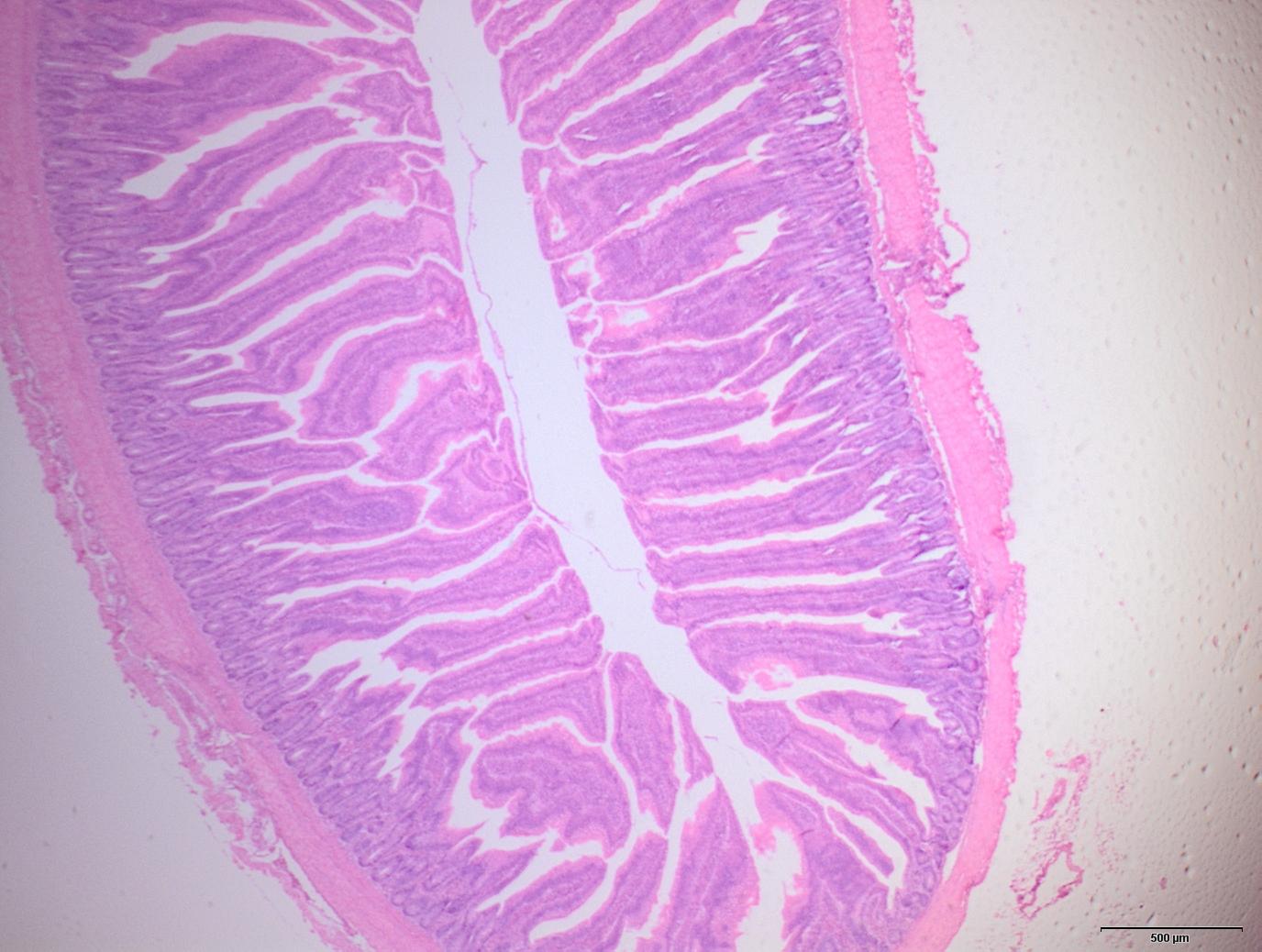

Supplement: Supplementary file 3 [file Data_Sheet_1.ZIP › Data sheet/Hematoxylin-eosin Staining/Jejunum/NE+TA600 group/8.jpg]

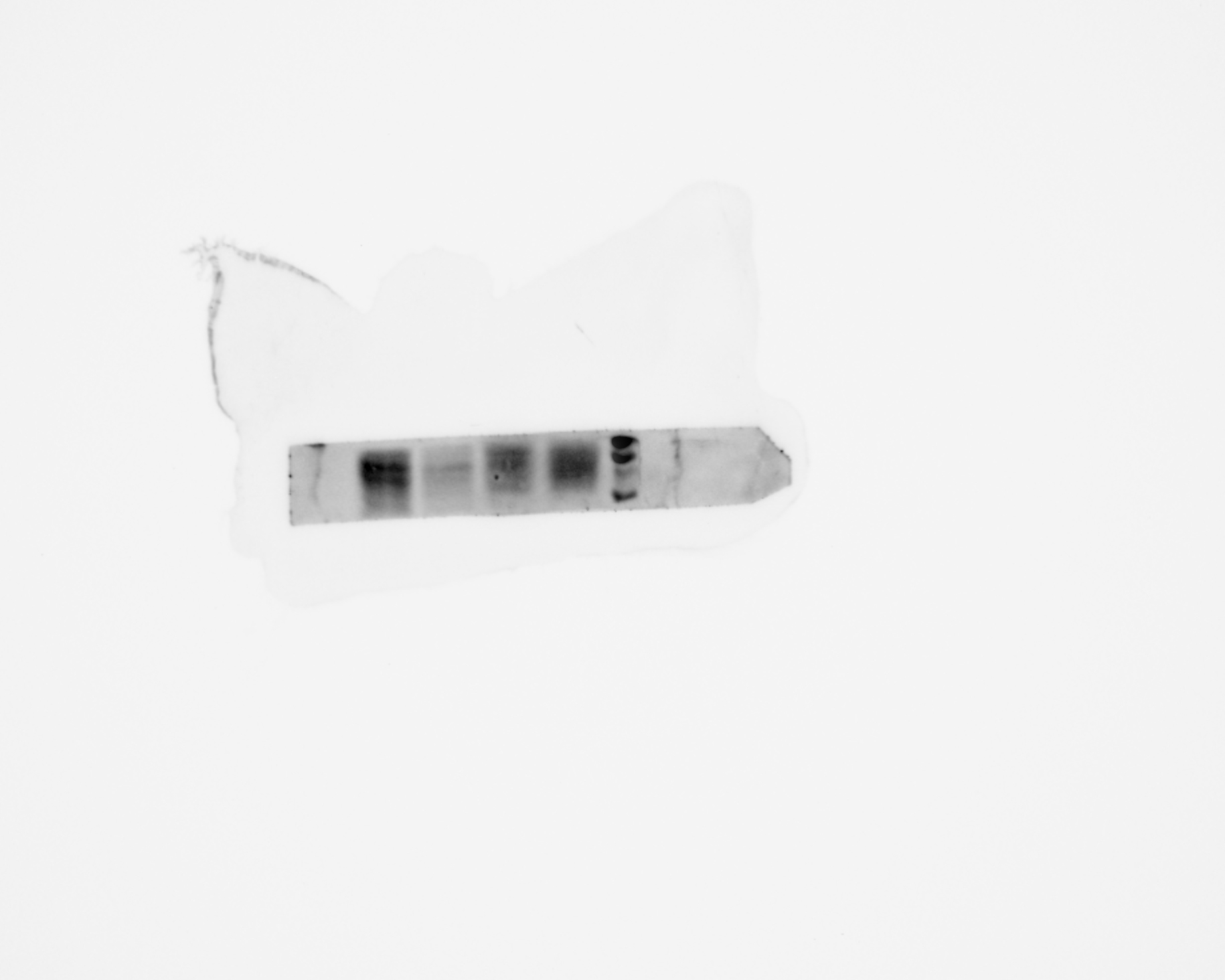

Supplement: Supplementary file 3 [file Data_Sheet_1.ZIP › Data sheet/Western Blot/HO1/Ileum/HO1-1.jpg]

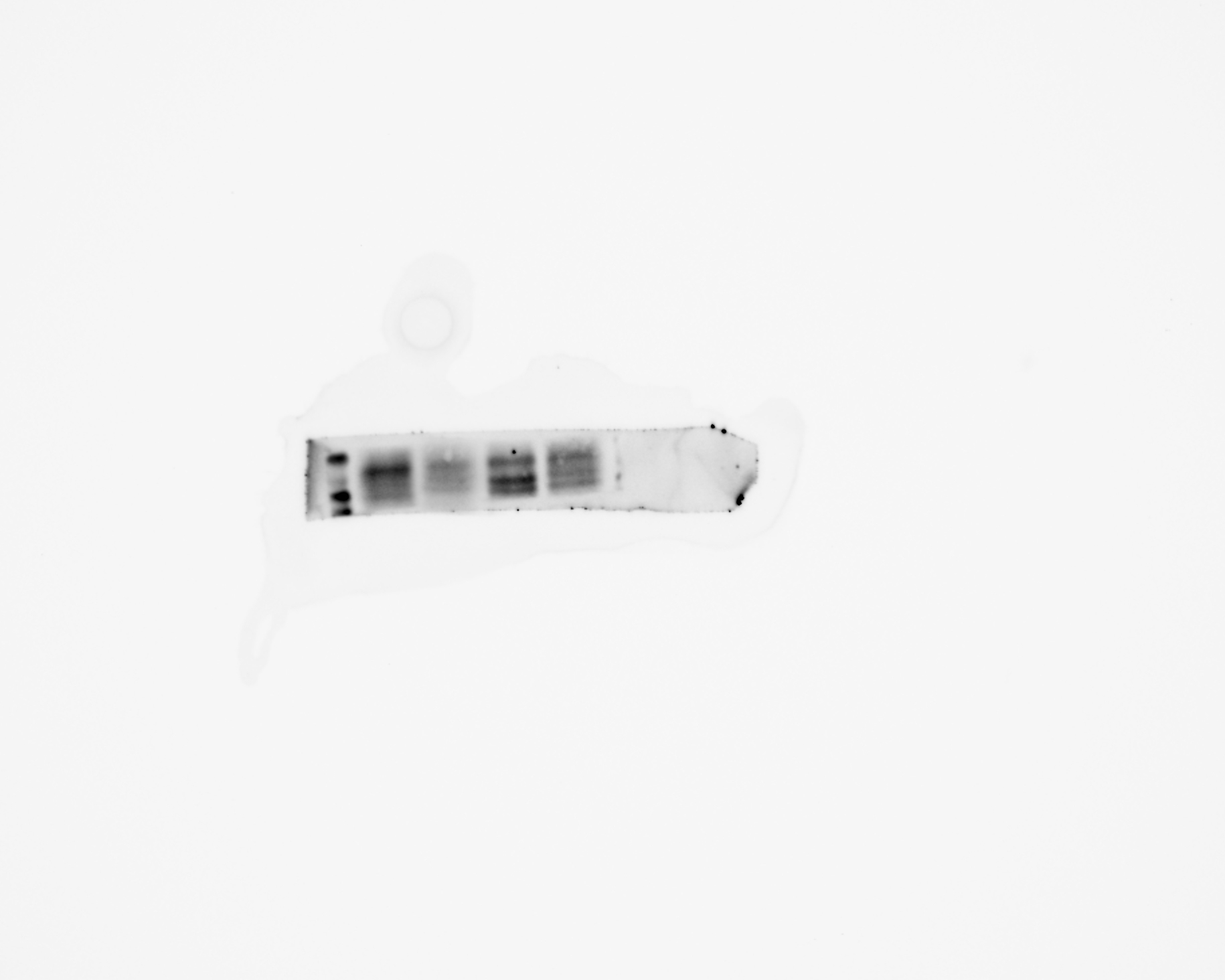

Supplement: Supplementary file 3 [file Data_Sheet_1.ZIP › Data sheet/Western Blot/HO1/Ileum/HO1-2.jpg]

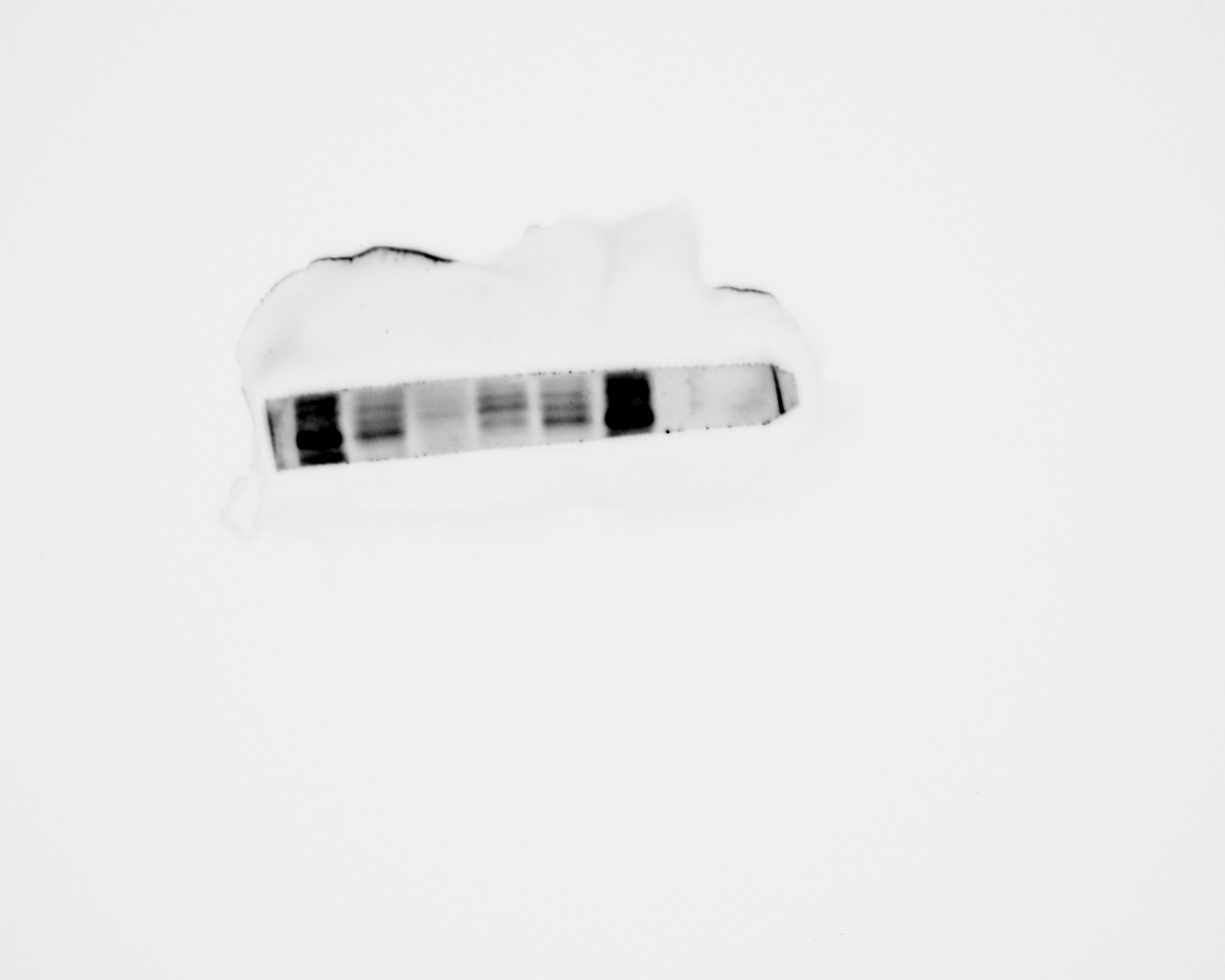

Supplement: Supplementary file 3 [file Data_Sheet_1.ZIP › Data sheet/Western Blot/HO1/Ileum/HO1-3.jpg]

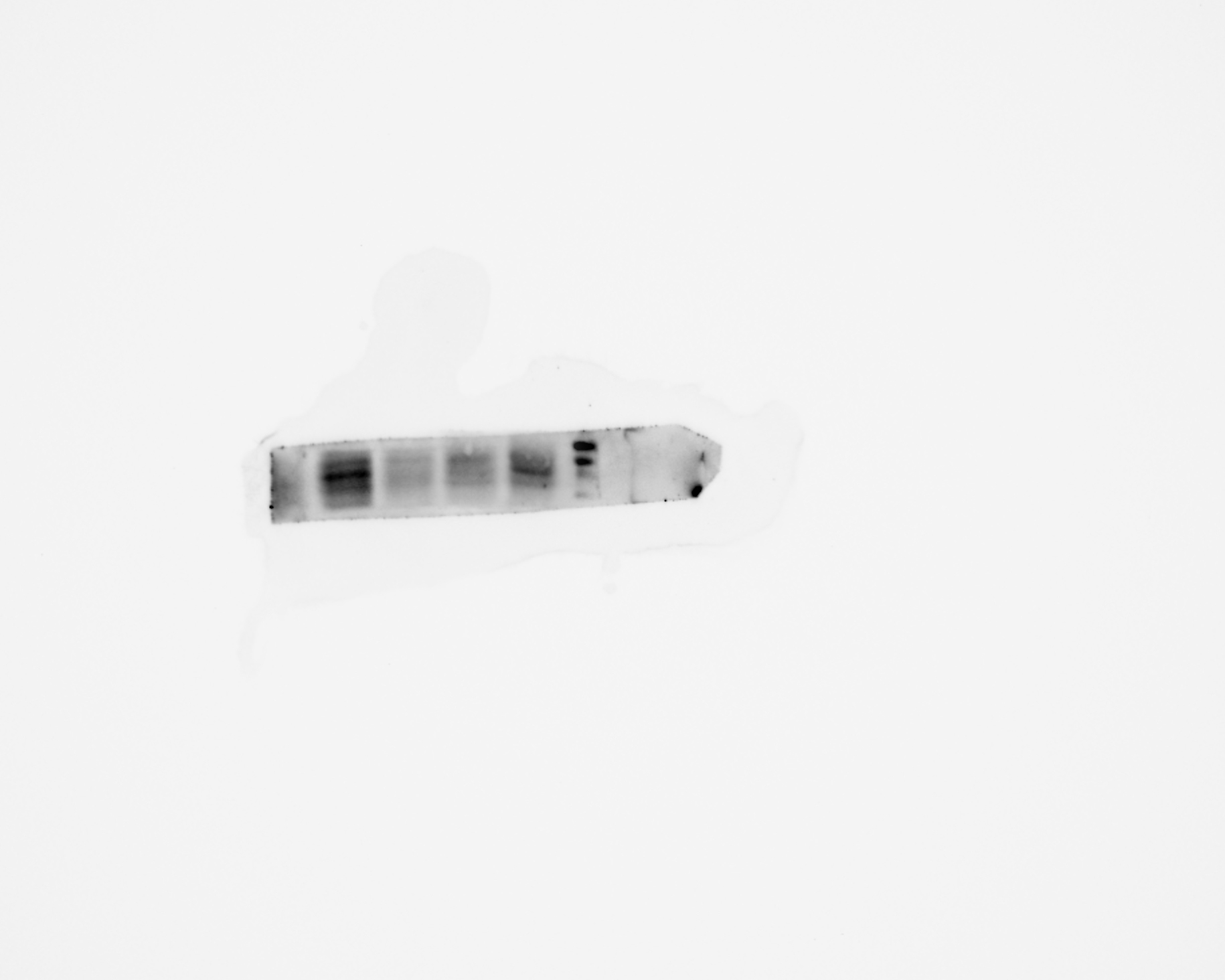

Supplement: Supplementary file 3 [file Data_Sheet_1.ZIP › Data sheet/Western Blot/HO1/Jejunum/HO1-1.jpg]

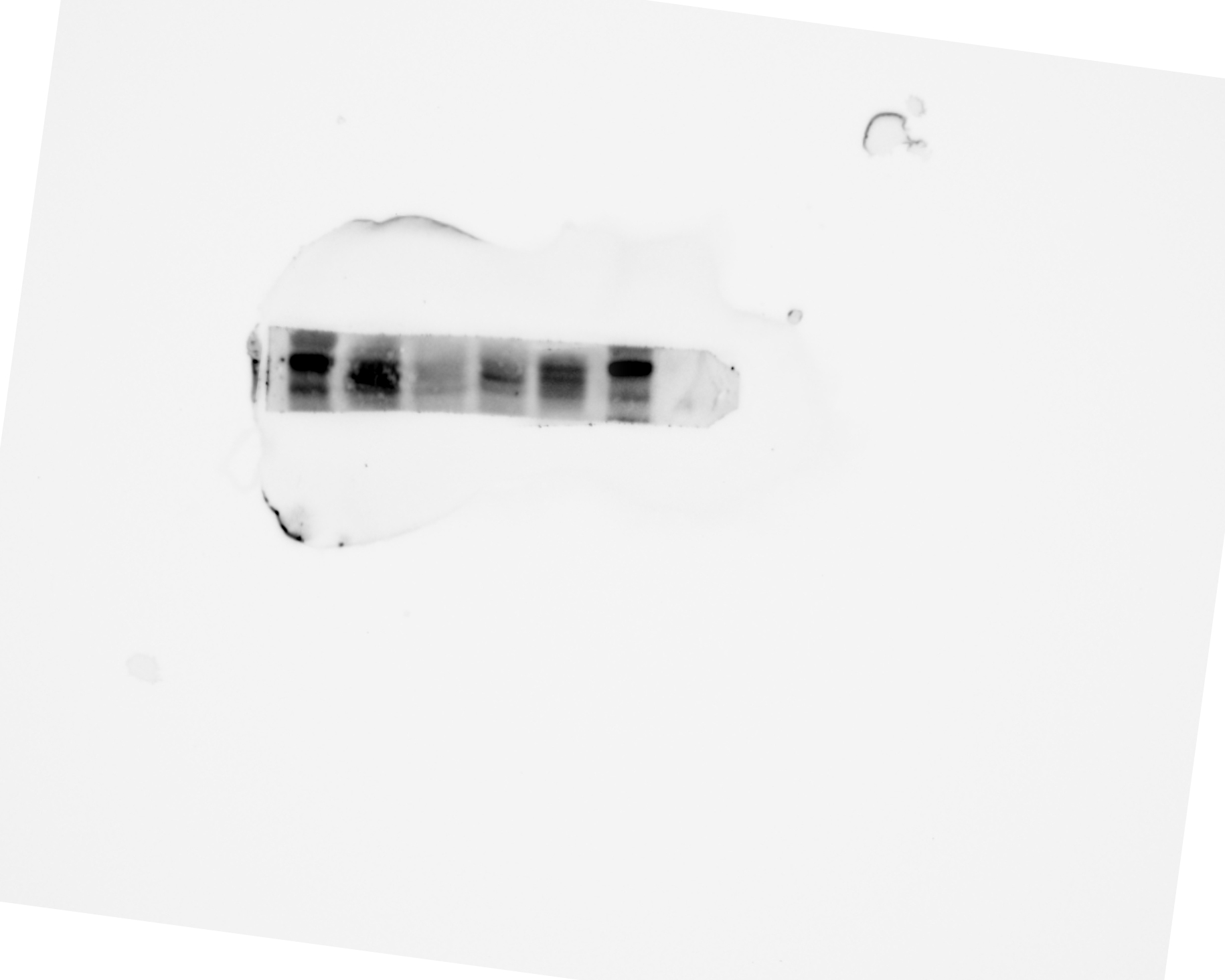

Supplement: Supplementary file 3 [file Data_Sheet_1.ZIP › Data sheet/Western Blot/HO1/Jejunum/HO1-2.jpg]

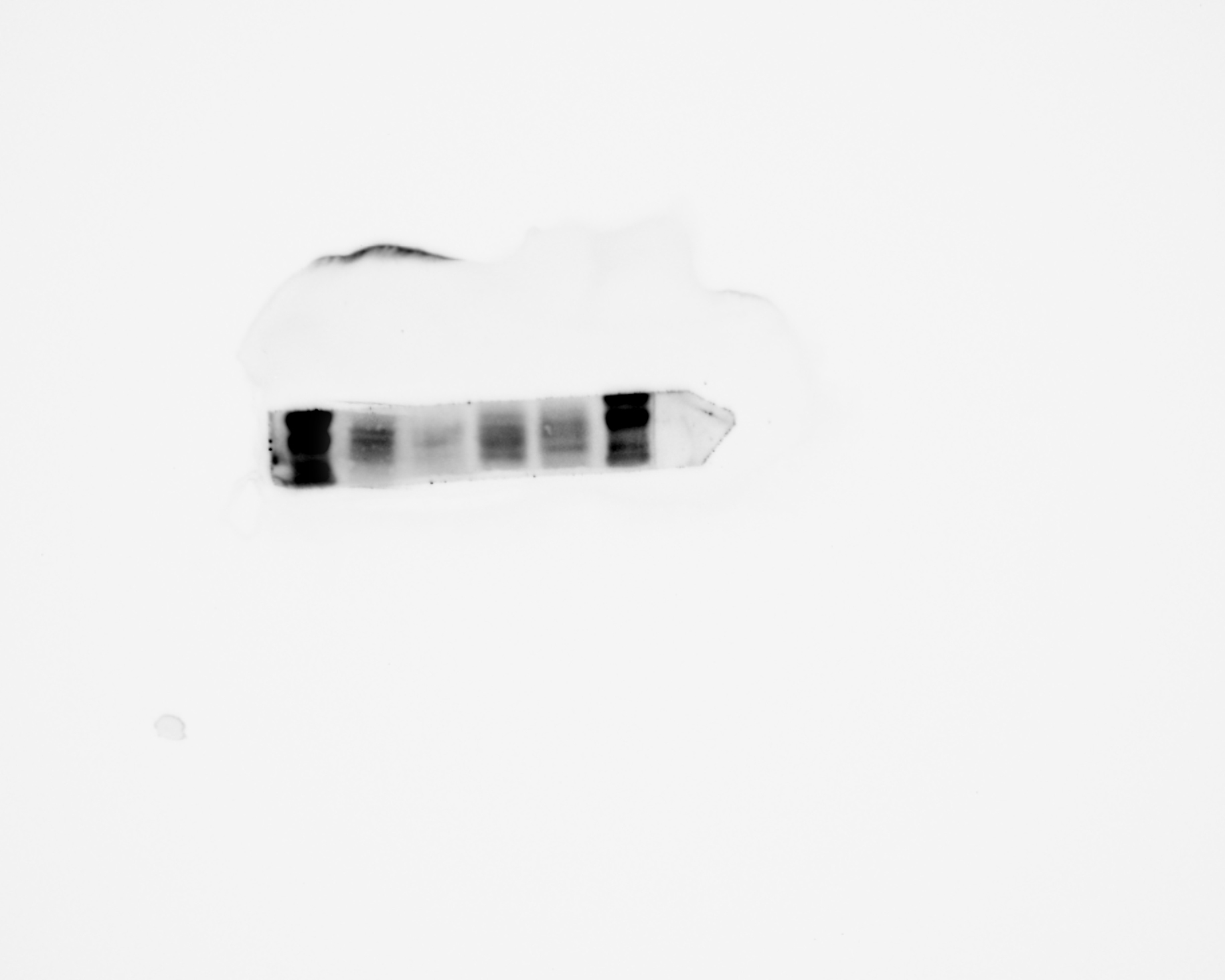

Supplement: Supplementary file 3 [file Data_Sheet_1.ZIP › Data sheet/Western Blot/HO1/Jejunum/HO1-3.jpg]

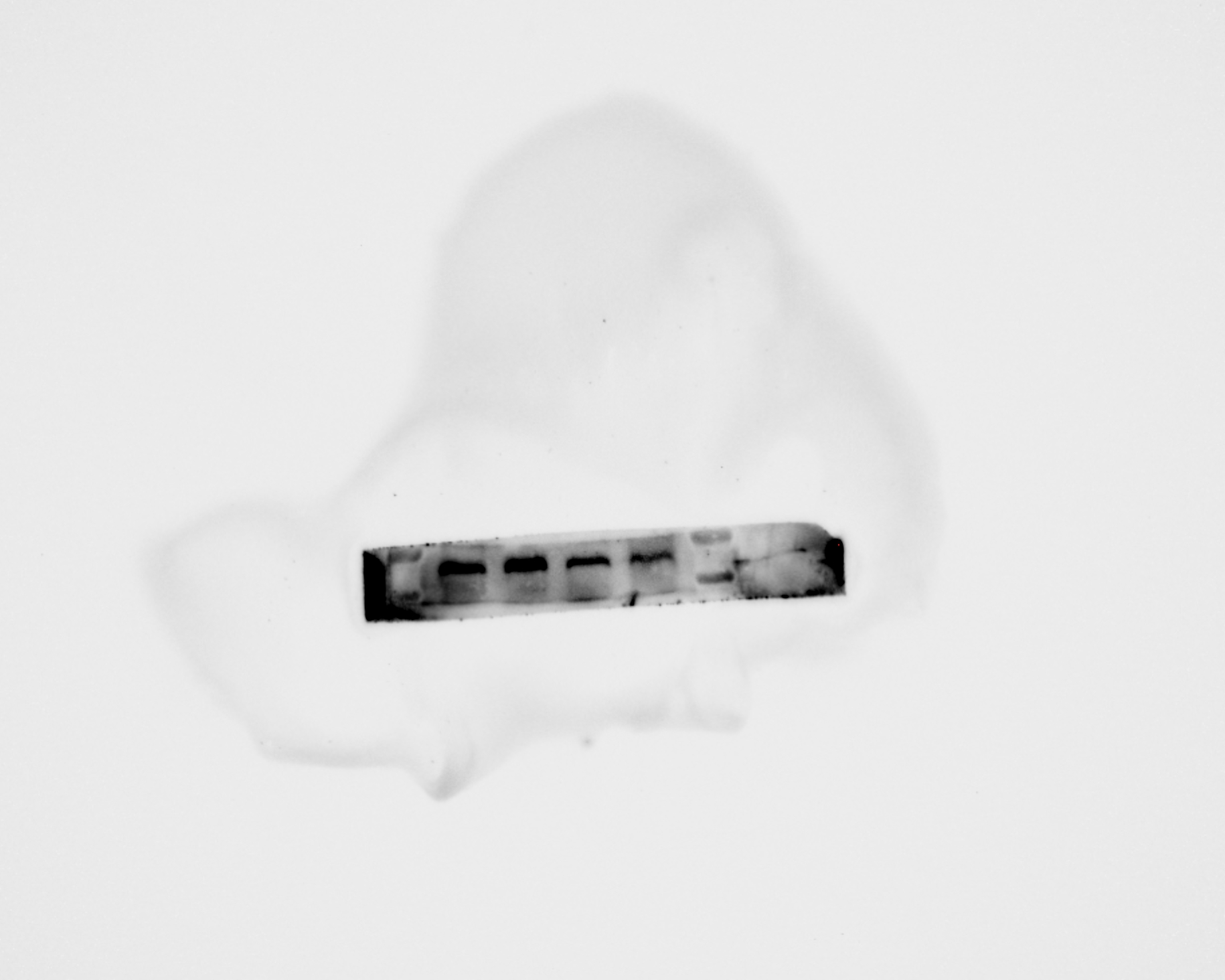

Supplement: Supplementary file 3 [file Data_Sheet_1.ZIP › Data sheet/Western Blot/Nrf2/Ileum/Nrf2-1.jpg]

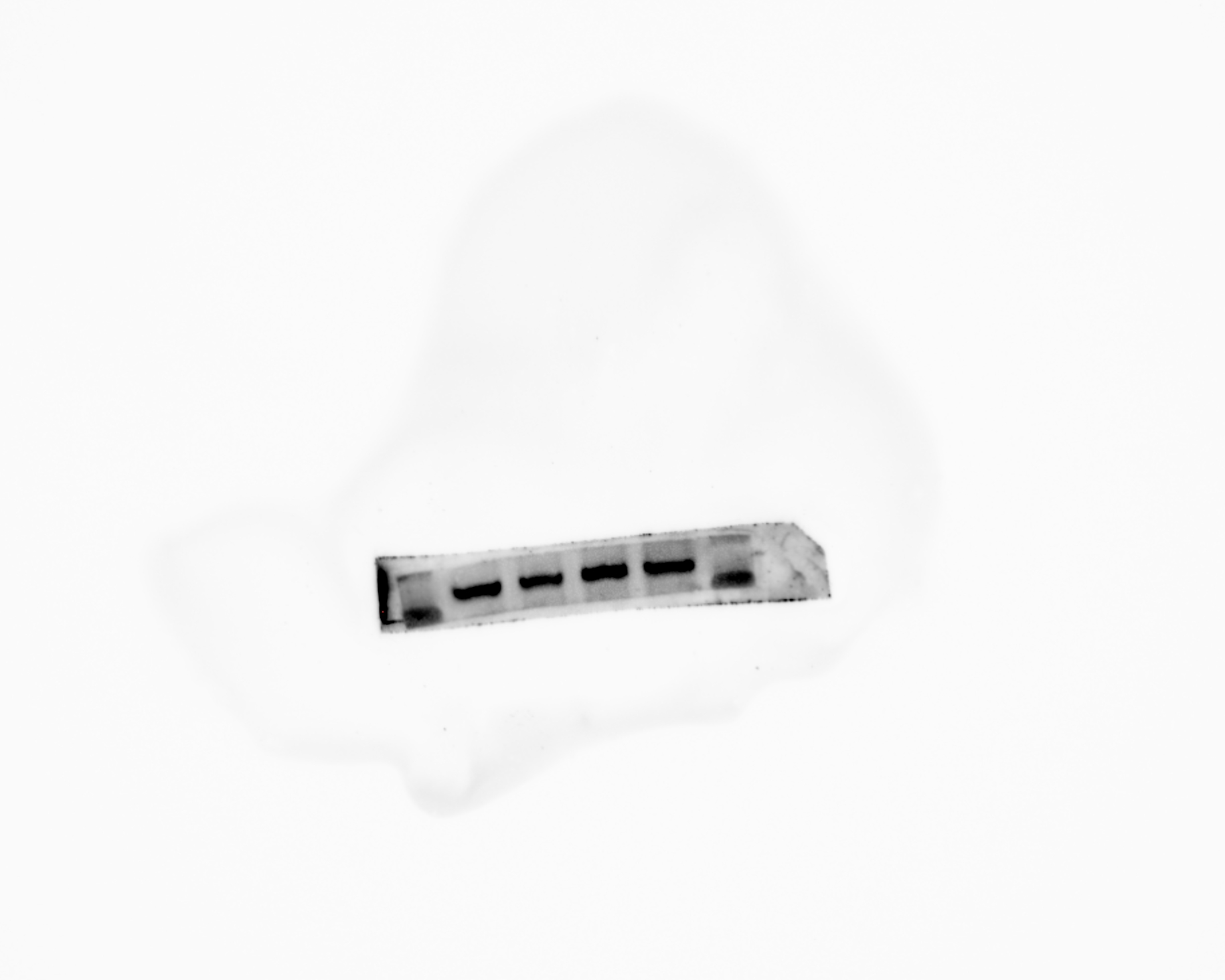

Supplement: Supplementary file 3 [file Data_Sheet_1.ZIP › Data sheet/Western Blot/Nrf2/Ileum/Nrf2-2.jpg]

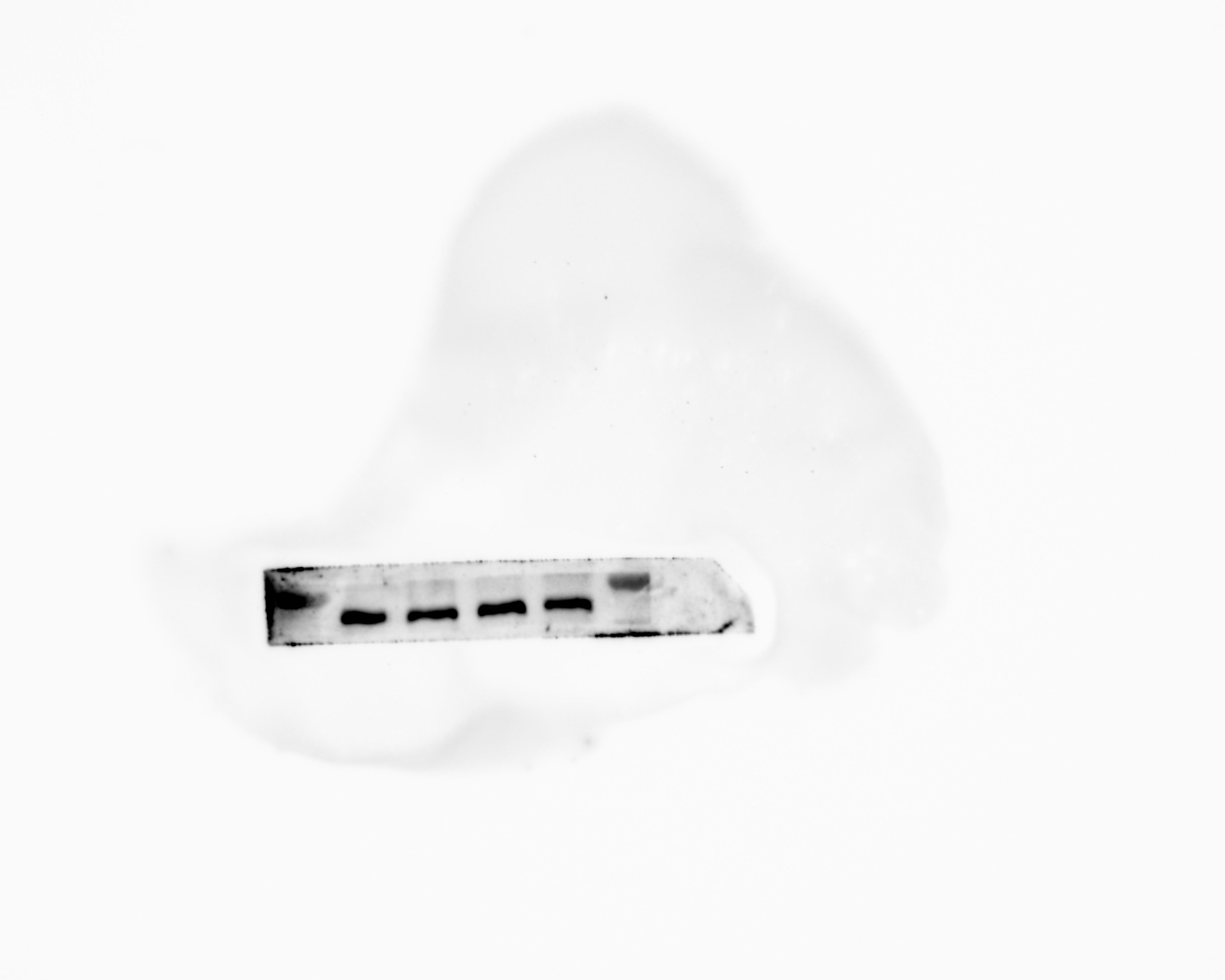

Supplement: Supplementary file 3 [file Data_Sheet_1.ZIP › Data sheet/Western Blot/Nrf2/Ileum/Nrf2-3.jpg]

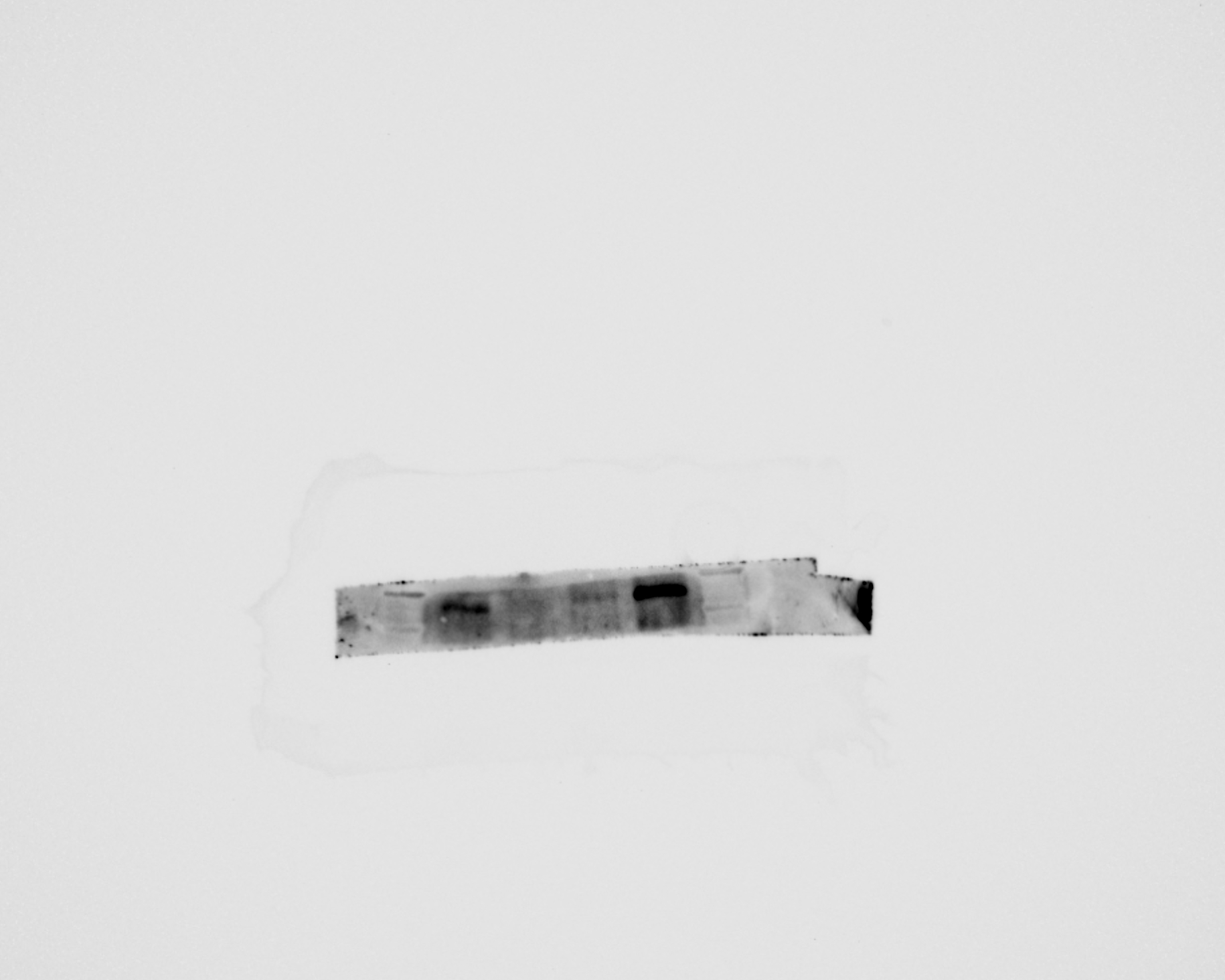

Supplement: Supplementary file 3 [file Data_Sheet_1.ZIP › Data sheet/Western Blot/Nrf2/Jejunum/Nrf2-1.jpg]

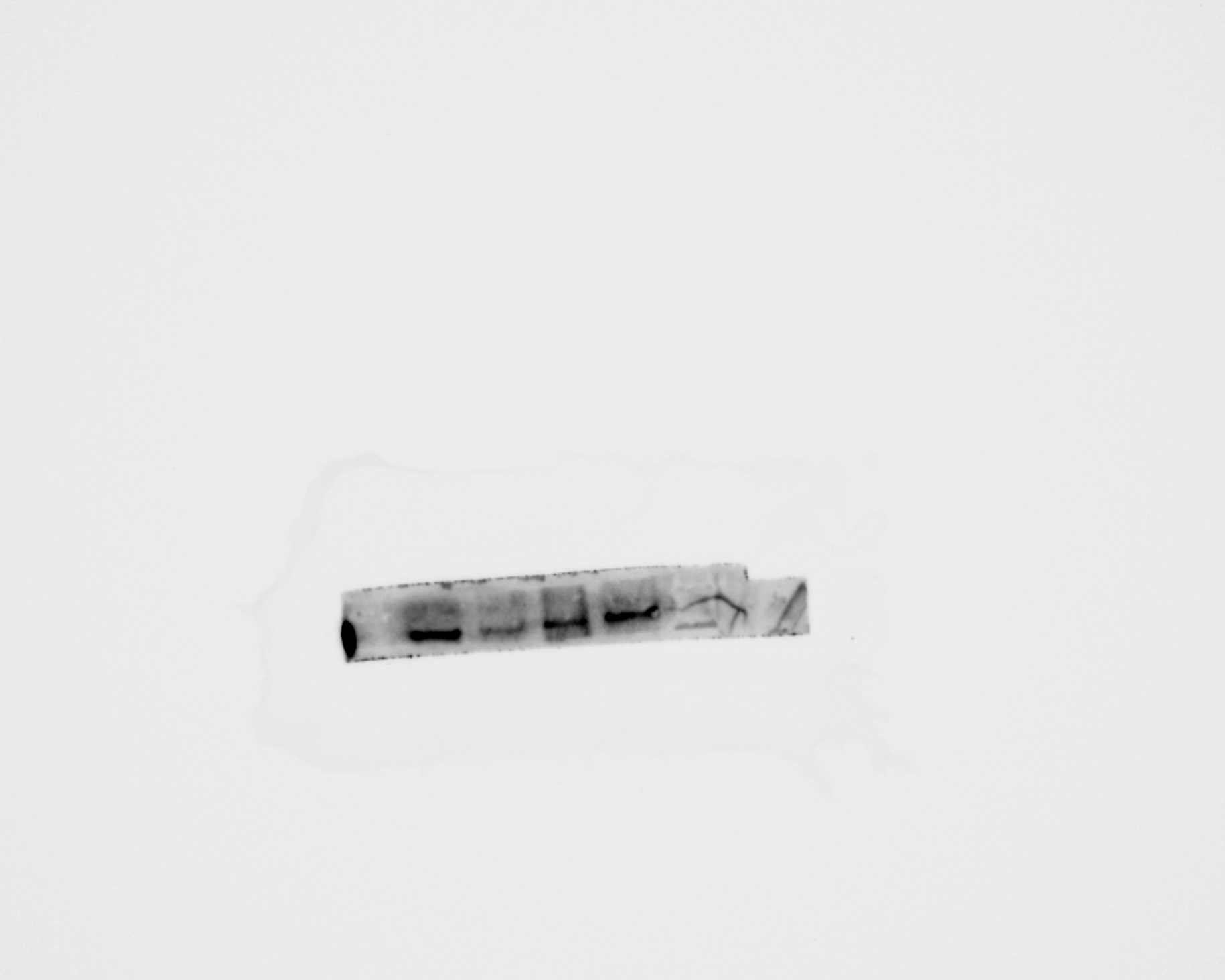

Supplement: Supplementary file 3 [file Data_Sheet_1.ZIP › Data sheet/Western Blot/Nrf2/Jejunum/Nrf2-2.jpg]

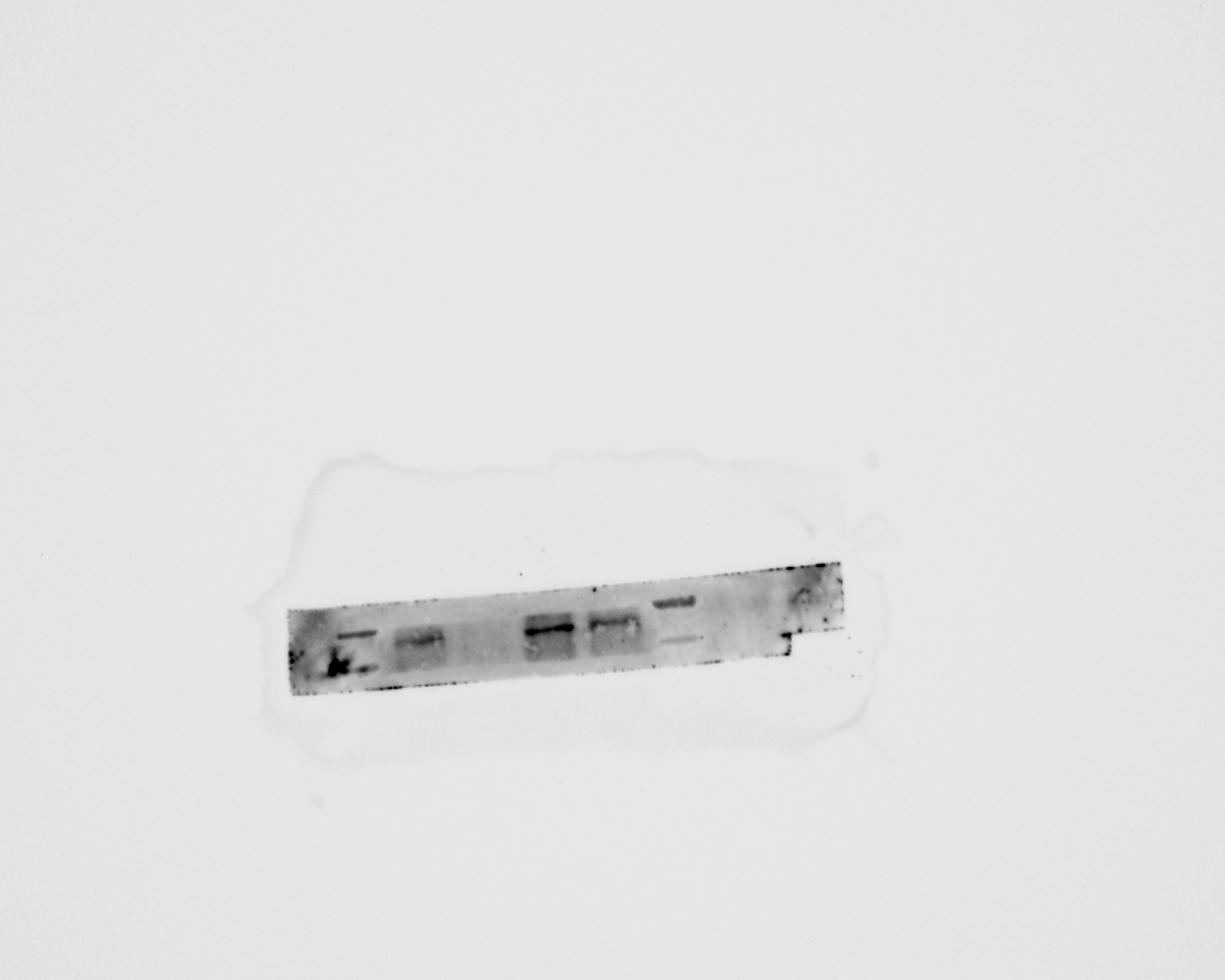

Supplement: Supplementary file 3 [file Data_Sheet_1.ZIP › Data sheet/Western Blot/Nrf2/Jejunum/Nrf2-3.jpg]

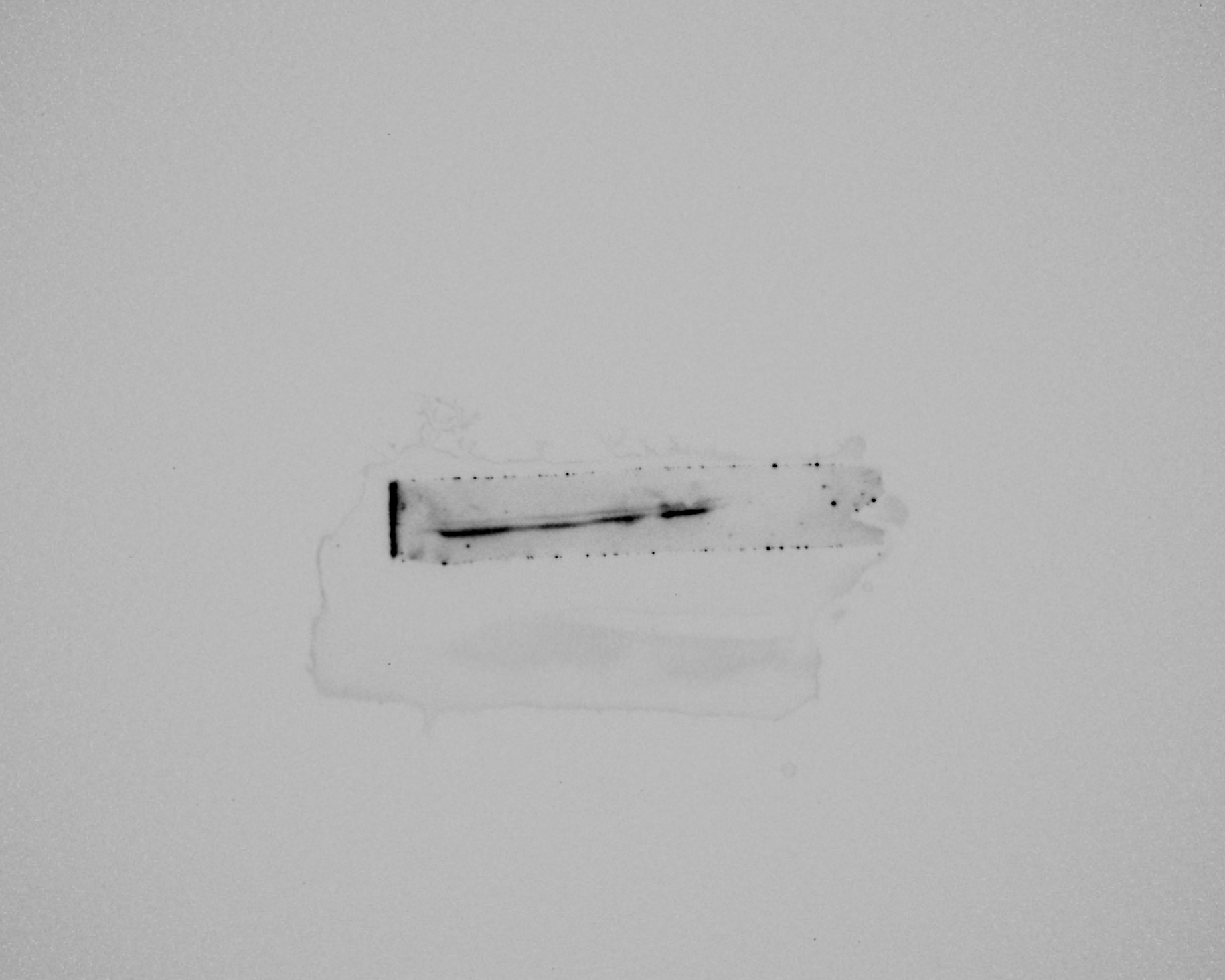

Supplement: Supplementary file 3 [file Data_Sheet_1.ZIP › Data sheet/Western Blot/SOD1/Ileum/SOD1-1.jpg]

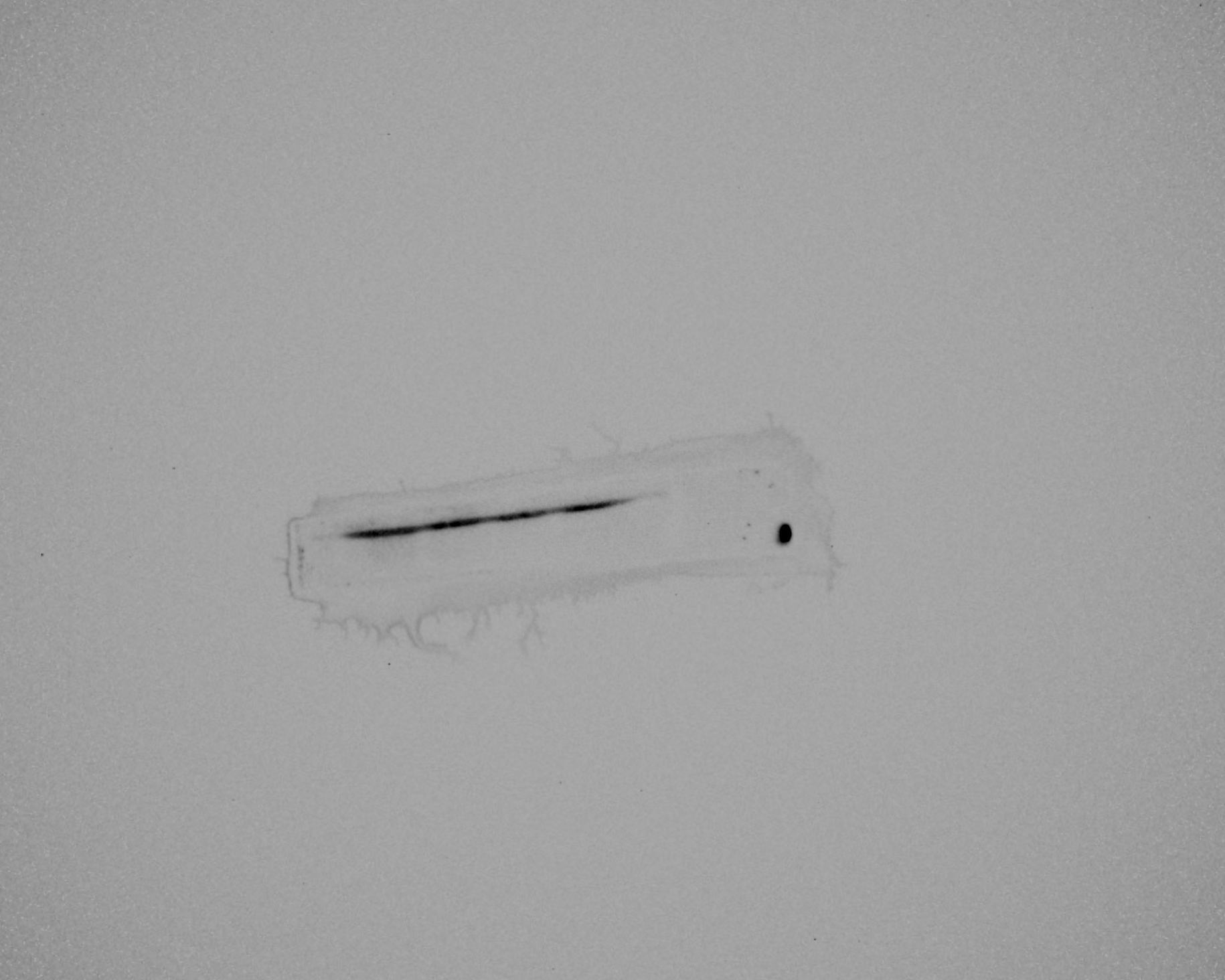

Supplement: Supplementary file 3 [file Data_Sheet_1.ZIP › Data sheet/Western Blot/SOD1/Ileum/SOD1-2.jpg]

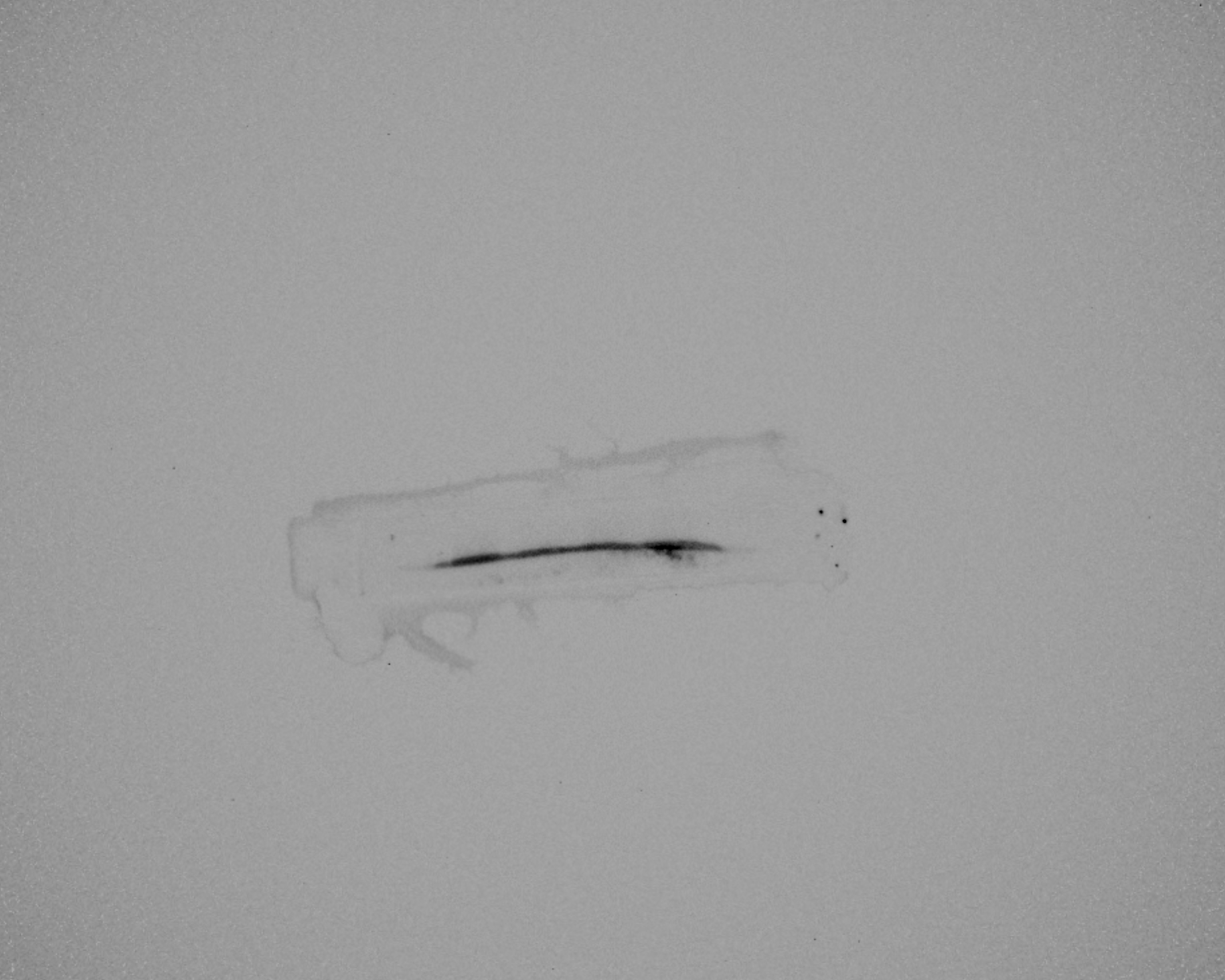

Supplement: Supplementary file 3 [file Data_Sheet_1.ZIP › Data sheet/Western Blot/SOD1/Ileum/SOD1-3.jpg]

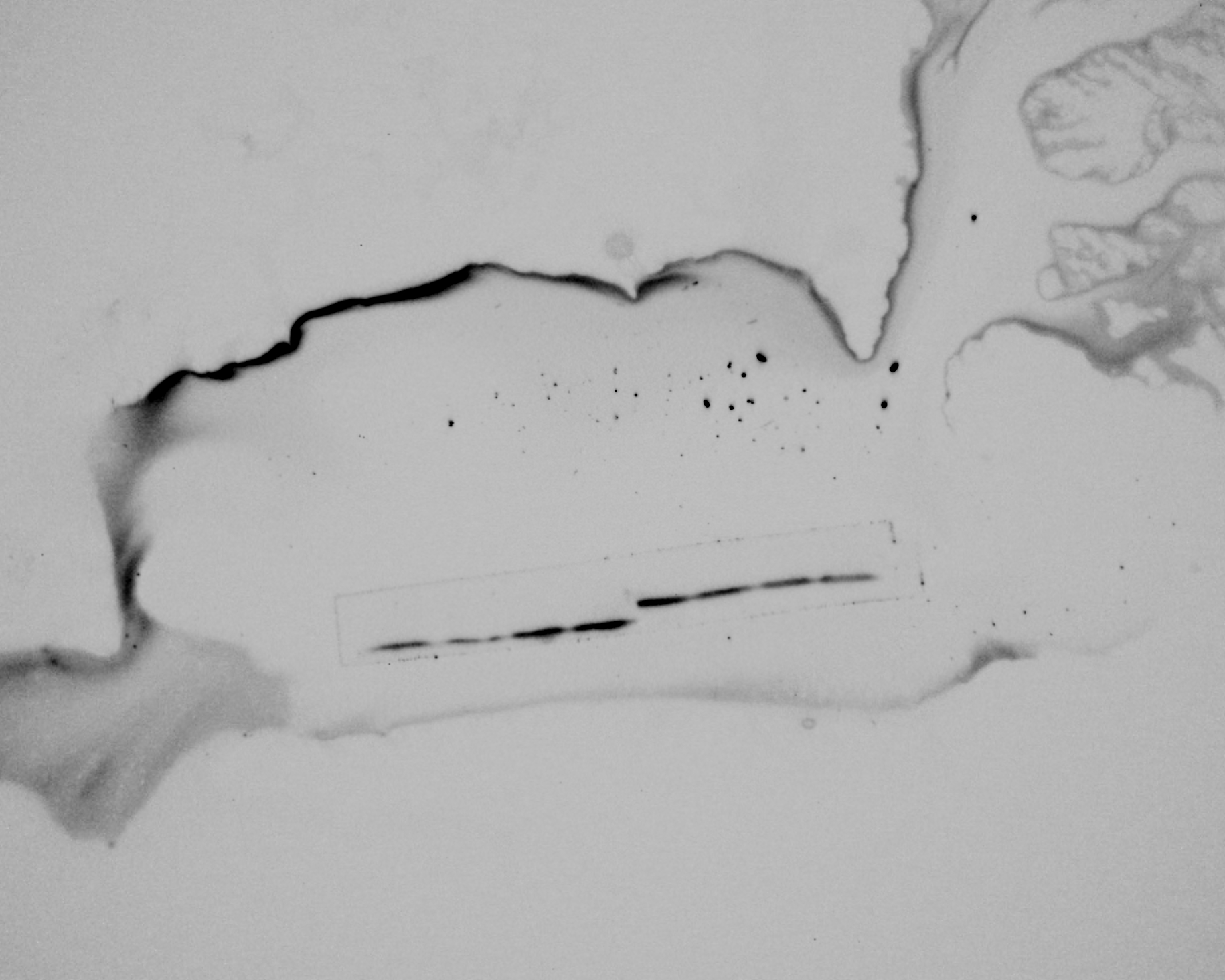

Supplement: Supplementary file 3 [file Data_Sheet_1.ZIP › Data sheet/Western Blot/SOD1/Jejunum/SOD1-1 2.jpg]

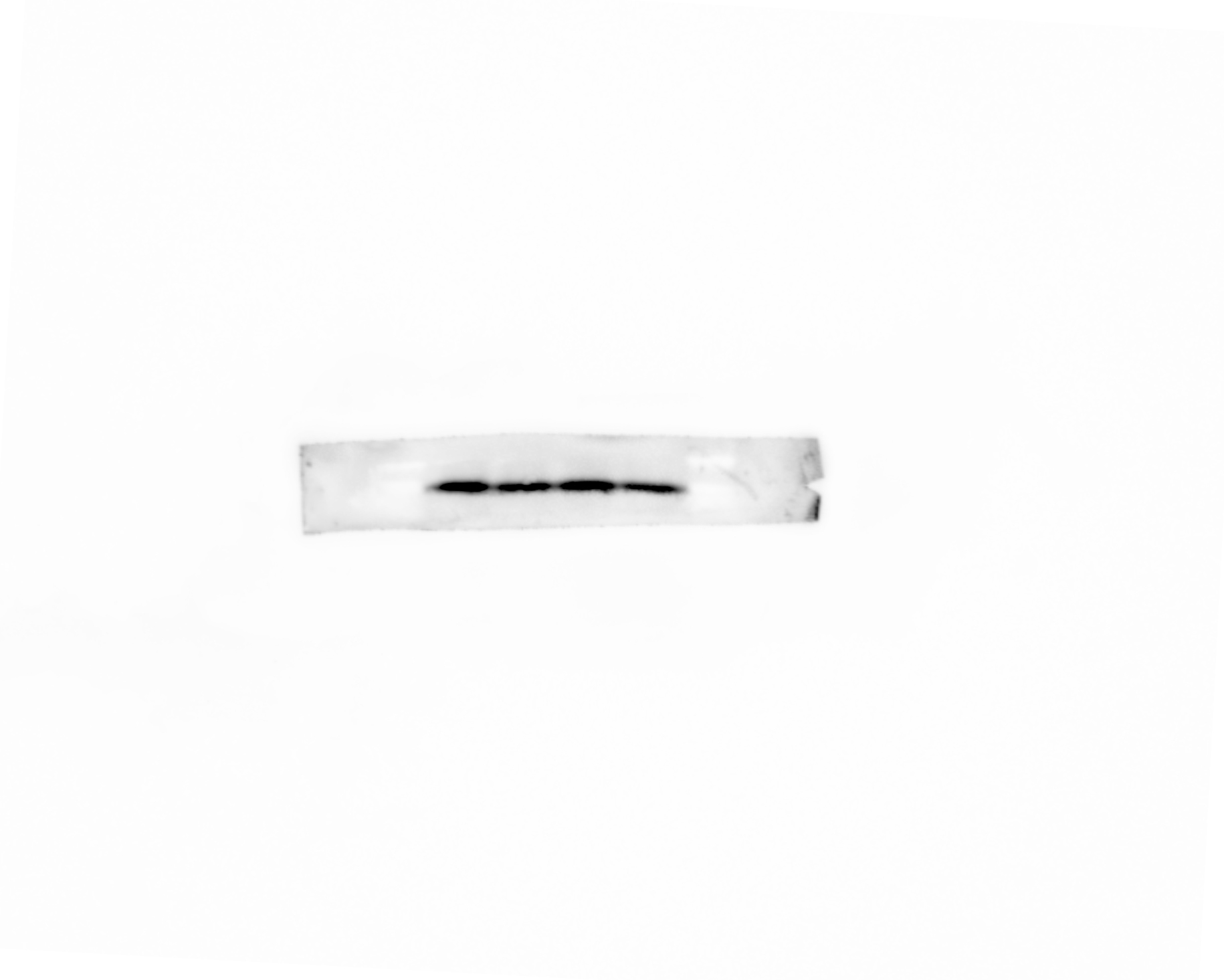

Supplement: Supplementary file 3 [file Data_Sheet_1.ZIP › Data sheet/Western Blot/SOD1/Jejunum/SOD1-3.jpg]

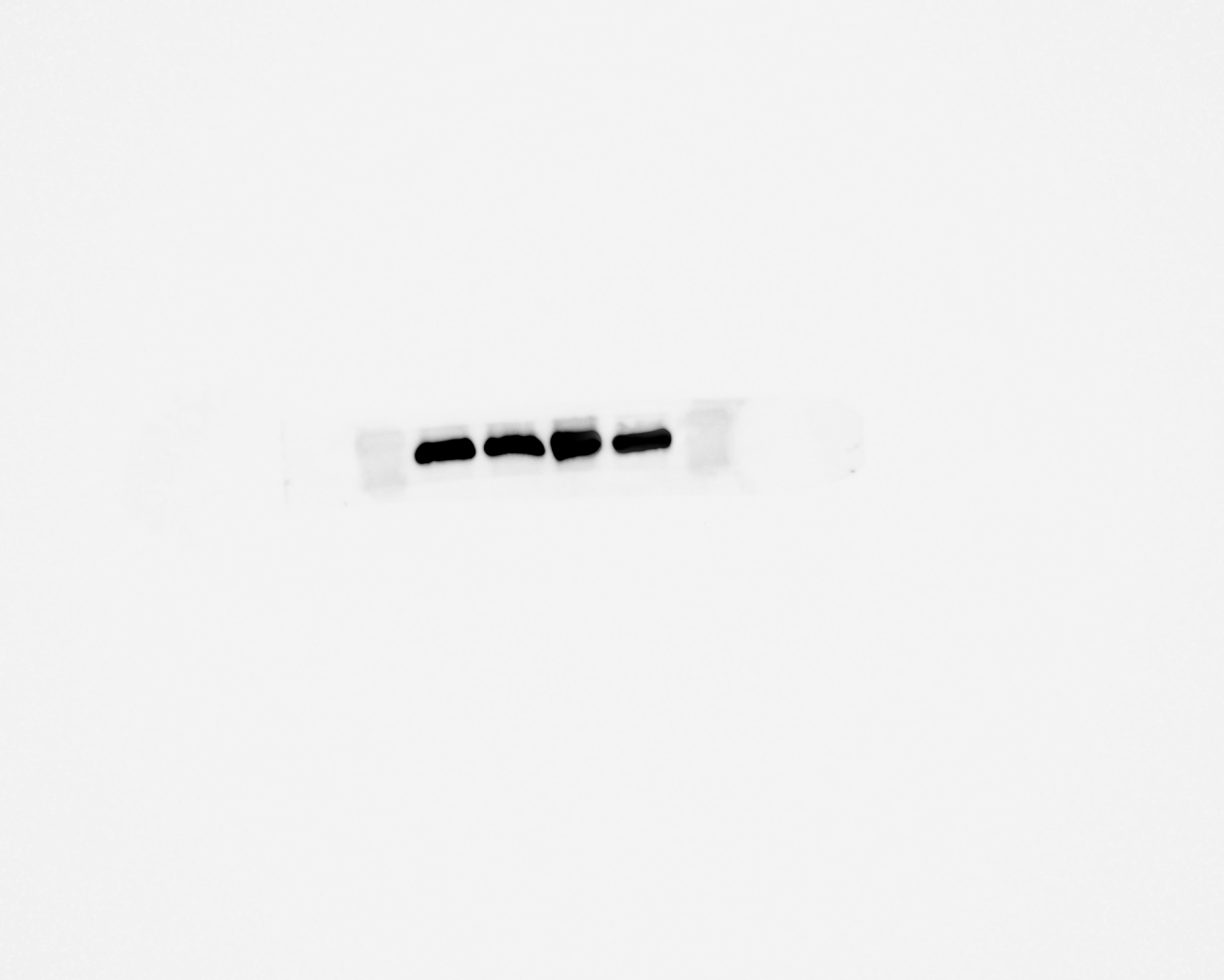

Supplement: Supplementary file 3 [file Data_Sheet_1.ZIP › Data sheet/Western Blot/a┬-actin/Ileum/a┬-actin 1.jpg]

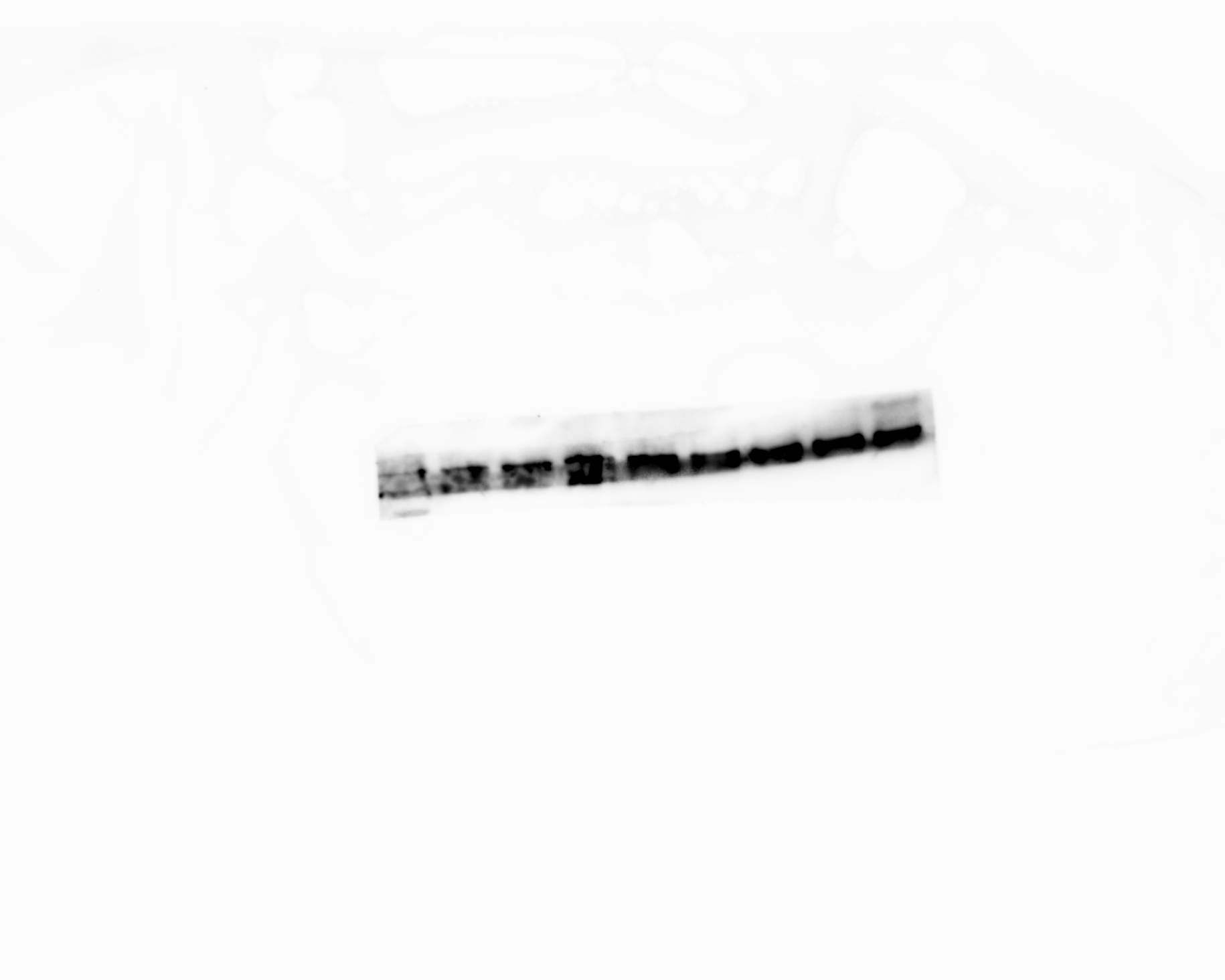

Supplement: Supplementary file 3 [file Data_Sheet_1.ZIP › Data sheet/Western Blot/a┬-actin/Ileum/a┬-actin 2.jpg]

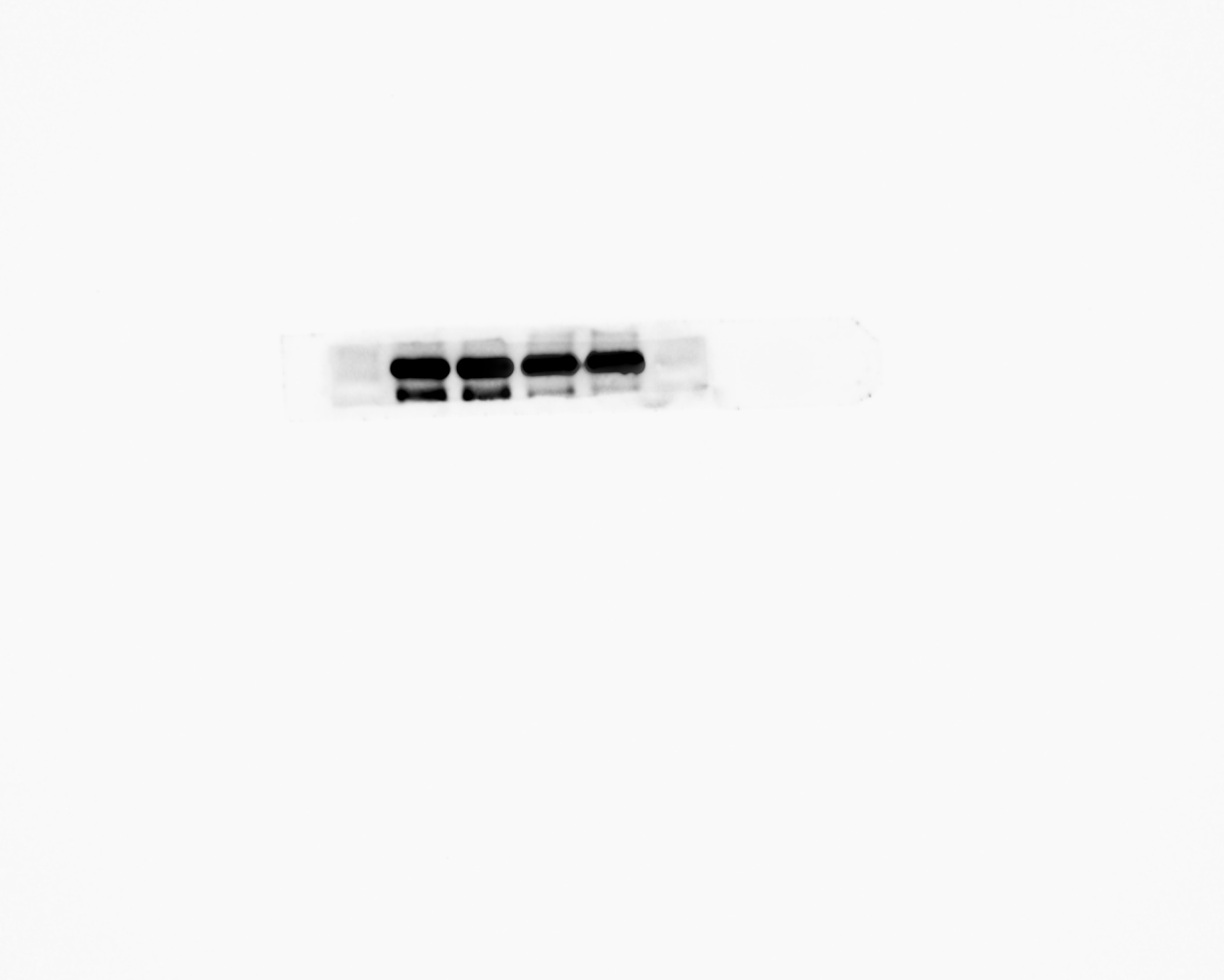

Supplement: Supplementary file 3 [file Data_Sheet_1.ZIP › Data sheet/Western Blot/a┬-actin/Jejunum/a┬-actin 1.jpg]

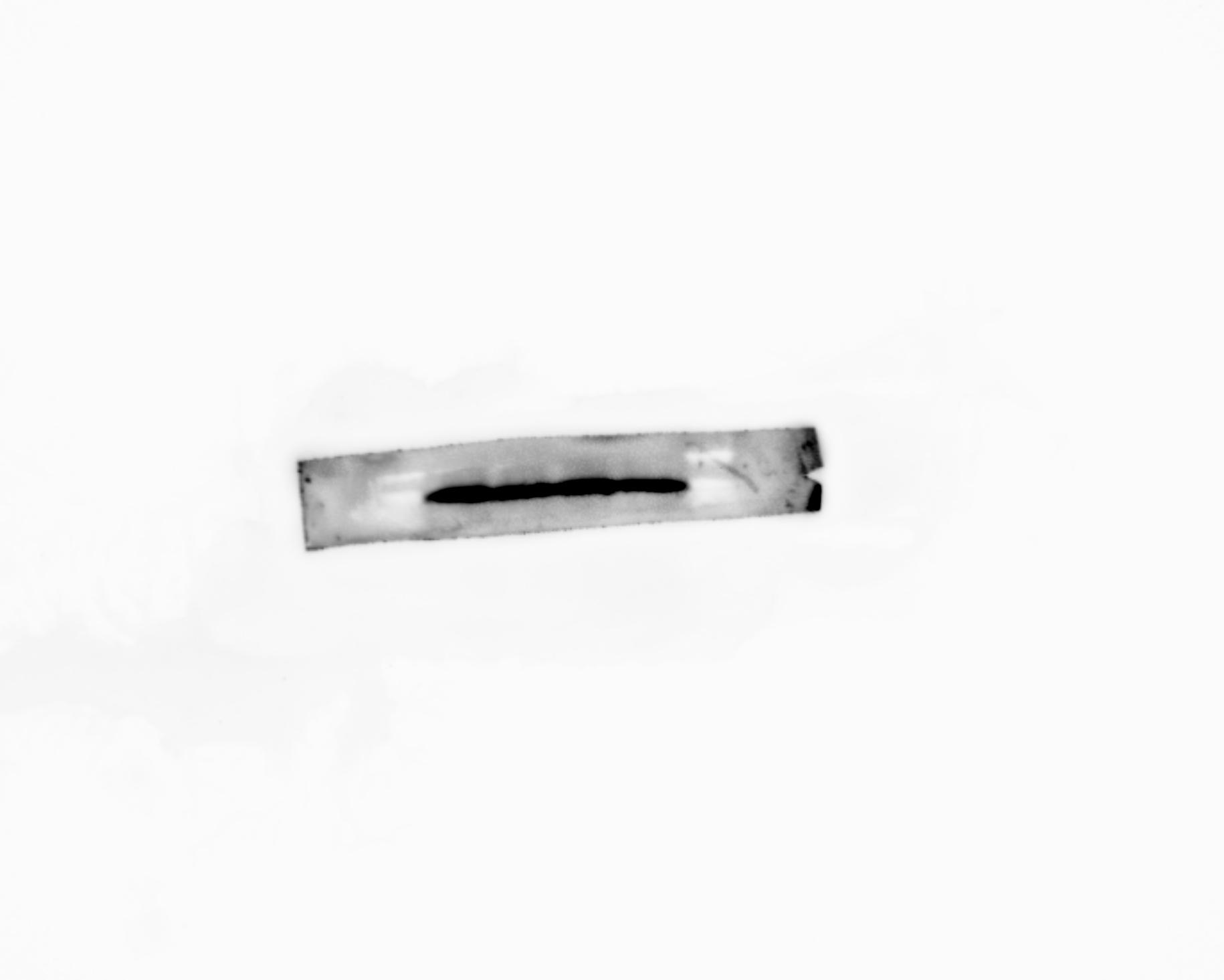

Supplement: Supplementary file 3 [file Data_Sheet_1.ZIP › Data sheet/Western Blot/a┬-actin/Jejunum/a┬-actin 2.jpg]
